# Application of a French cattle pangenome, from structural variant discovery to association studies on key phenotypes

**DOI:** 10.1186/s12711-025-01012-x

**Published:** 2025-10-23

**Authors:** Valentin Sorin, Maulana Mughitz Naji, Clément Birbes, Cécile Grohs, Clémentine Escouflaire, Sébastien Fritz, Camille Eché, Camille Marcuzzo, Amandine Suin, Cécile Donnadieu, Christine Gaspin, Carole Iampietro, Denis Milan, Laurence Drouilhet, Gwenola Tosser-Klopp, Didier Boichard, Christophe Klopp, Marie-Pierre Sanchez, Mekki Boussaha

**Affiliations:** 1 INRAE, AgroParisTech, GABI, Université Paris Saclay, 78350 Jouy en Josas, France; 2 BioInfoMics, MIAT UR875, Sigenae, INRAE, Genotoul Bioinfo, 31326 Castanet-Tolosan, France; 3 Eliance, 149 Rue de Bercy, 75012 Paris, France; 4 INRAE, US 1426, GeT-PlaGe, Genotoul, France Génomique, Université de Toulouse, 31326 Castanet-Tolosan, France; 5 GenPhySE, INRAE, ENVT, Université de Toulouse, 31326 Castanet-Tolosan, France

## Abstract

**Background:**

The current cattle reference genome assembly, a pseudo-linear sequence produced using sequences from a single Hereford cow, represents a limitation when performing genetic studies, especially when investigating the whole spectrum of genetic variations within the species. Detecting structural variations (SVs) poses significant challenges when relying solely on conventional methods of sequencing read mapping to the current bovine genome assembly.

**Results:**

In this study, we used long-reads (LR) and bioinformatic tools to construct a comprehensive bovine pangenome, using as a backbone the Hereford ARS-UCD1.2 reference genome assembly, and incorporating genetic diversity of 64 good quality de novo genome assemblies representing 14 French dairy and beef cattle breeds. Using a combination of complementary approaches, we explored the pangenome graph and identified 2.563 Gb of sequences common to all samples, and cumulated 0.295 Gb of variable sequences. Notably, we discovered 0.159 Gb of novel sequences not present in the current reference genome assembly. Our analysis also revealed 109,275 SVs, of which 84,612 were bi-allelic. These included 27,171 insertions and 24,592 deletions, while the remaining 32,849 SVs corresponded to alternate allele sequences defined as sequence substitutions between the reference genome and the sample sequence. Genome-wide association studies using SNPs and a panel of 221 SVs, shared between the pangenome and the EuroGMD chip, revealed well-known QTLs across the genome for the Holstein, Montbéliarde and Normande breeds. Among those, a QTL on chromosome 11 presents an SV with a highly significant effect on stature in the Holstein breed. This SV is a 6.2 kb deletion affecting the 5’UTR, first exon and part of the first intron of the *MATN3* gene, suggesting a potential regulatory and coding effect.

**Conclusions:**

Our study provides new insights into the genetic diversity of 14 French dairy and beef breeds and highlights the utility of pangenome graphs in capturing structural variation. The identified SV associated with stature highlights the importance of integrating SVs into GWAS for a more comprehensive understanding of complex traits.

**Supplementary Information:**

The online version contains supplementary material available at 10.1186/s12711-025-01012-x.

## Background

In many species, genetic analyses have traditionally relied on a single linear reference assembly, often used for sequence alignments, identification of genomic variations, and genome annotation. The current ARS-UCD1.2 bovine reference genome assembly (*Bos taurus taurus*) was initially constructed using whole-genome sequencing derived from an inbred Hereford cow (L1 Dominette 01449), and subsequently upgraded to long read-based assembly in 2020 [1, 2]. However, this reference assembly is still incomplete and imperfect. Indeed, several studies have pointed out substantial gaps, including a 3.2 Gb Charolais PacBio HiFi assembly [3] and a 3.14 Gb near complete Wagyu assembly that as a few telomere-to-telomere chromosomes [4], which have uncovered over 400 Mb of genomic sequences absent from the ARS-UCD1.2 cattle reference assembly. Beyond these missing sequences, a single reference assembly does not effectively represent the genetic diversity that can be encompassed within the species, which may lead to biases in genomic studies. Over the years, improvements of sequencing technologies, combined with the development of bioinformatic methods for genome assembly, have greatly improved genome contiguity and accuracy, thereby facilitating the characterization of a wide range of genomic variations. However, the current linear reference genome assembly still contains hundreds of gaps [2], including stretches of missing sequences spanning hundreds of megabases (Mb) (~ 4.6 Mb of N-stretch), and only represents one haplotypic reference of the species [5, 6].

Recent advances in long-read (LR) sequencing technologies now enable the production of more continuous genome sequences, with accuracy comparable to those obtained with short-read (SR) sequencing [7]. SR sequencing is especially valuable for the identification and characterization of single nucleotide polymorphisms (SNPs) as well as small insertions and deletions (InDels) in non-repeated parts of the assemblies. In contrast, LR sequencing is more effective in detecting complex SVs, which includes large deletions, insertions, inversions, duplications, and translocations. Moreover, high-quality LR sequencing also enable the detection of small variant in repeated part of the assemblies. Additionally, LR sequences facilitates the creation of high-quality de novo genome assemblies, which serve as the foundation for multi-assembly graphs, commonly referred to as pangenomes [8–10].

Pangenomes are constructed as graphs that integrate assemblies from multiple individuals [11, 12]. Their structure consists of nodes representing genomic sequences and edges that link consecutive sequences without any overlap. Nodes can be classified either as part of the core genome when sequences are shared by all individuals used to construct the graph, or as part of the flexible genome when genomic sequences are found in only a subset of individuals. These pangenome graphs are of particular interest as they provide a more comprehensive representation of the genetic diversity within a species, hence facilitating a more detailed identification of mutations that contribute to phenotypic variations. However, the extent of genetic diversity represented by a pangenome graph is influenced by both the completeness of the input genome assemblies as well as the effectiveness of the tools used to construct the graph. For instance, highly repetitive regions such as centromeres and telomeres are often excluded from pangenome analyses, since tools such as Minigraph struggle to precisely anchor sequences within these regions [13, 14].

In recent years, several computational tools have been developed for the construction of pangenome graphs, each with different specificities. The Minigraph software [15] efficiently detects large structural variants (≥ 50 bp) using a reference genome as a backbone but it is not well-suited for studying small genomic variations. The Minigraph-Cactus tool [16] extends the previous approach by incorporating smaller DNA variations, such as SNPs and InDels. Both Minigraph and Minigraph-Cactus allow the iterative addition of assemblies based on their phylogenetic proximity to the reference genome. Alternatively, the pangenome graph builder (pggb) uses a reference-free approach, thus reducing bias in graph construction [17]. Although computationally demanding, pggb enables fine-scale genetic variant detection across entire chromosomes, similar to Minigraph-Cactus.

In the present study, we used Minigraph to construct a whole-genome pangenome graph using the ARS-UCD1.2 reference genome assembly as a backbone and 64 de novo genome assemblies representing 14 French cattle breeds. Using this graph, we carried out a comprehensive analysis of SVs, assessing their contribution to population structure based on genotypes, and investigating their associations with key phenotypes in the three main French dairy cattle breeds.

## Methods

### De novo genomes assemblies processing

In this study we used 64 new de novo genome assemblies from animals corresponding to 14 French dairy and beef breeds. The number of individuals per breed ranged from two (Rouge Flamande) to eight (Holstein) (Table 1, see Additional file 1, Table S1). Our panel of de novo genome assemblies contains both widely used breeds (*e.g.* Holstein, Normande and Montbéliarde) and more rustic and local French breeds (*e.g.* Vosgienne, Tarentaise and Abondance).

**Table 1:** Distribution of assemblies per breed

Breed	Breed abbreviation	Number of assemblies
Abondance	ABO	5
Aubrac	AUB	7
Blonde d’Aquitaine	BAQ	4
Brown Swiss	BSW	5
Charolaise	CHA	4
Holstein	HOL	8
Limousine	LIM	2
Montbéliarde	MON	5
Normande	NMD	7
Parthenaise	PAR	3
Rouge Flamande	RDC	2
Simmental	SIM	3
Tarentaise	TAR	5
Vosgienne	VOS	4
Total		64

The 64 de novo genomes were produced by assembling PacBio CLR (Continuous Long Reads) using the wtdbg2 version 2.5 de novo genome assembler [18] and default parameters as described in the study of Eché et al. [3]. As PacBio CLR technology produces long noisy reads, a multi-step polishing process was applied using for each sample both the CLR long and Illumina short reads in order to improve the quality of the genome assembly sequences. Firstly, raw CLR data were aligned to the primary assembly contigs using pbmm2, a tool from the SMRTLink v12.0 workflow manager [19], and GCpp, a PacBio tool to compute a genomic consensus from these alignments [20]. Secondly, two additional rounds of polishing were applied using high-quality Illumina SR data with the Pilon software (v1.24) [21], enabling the correction of small-scale sequence errors across the assemblies. Finally, contigs were scaffolded to chromosome-level assemblies using the RagTag software (v2.1.0) [22], with the ARS-UCD1.2 bovine reference genome as the backbone [2].

We assessed the quality of the genome assemblies using three main metrics: total assembly length, N50 score, and completeness assessment. Completeness of the assemblies was assessed using the BUSCO score (v5.4.7) [23] with the mammalian single-copy orthologous gene dataset (mammalia_odb10. 2024-01-08) as a reference.

### Construction of a pangenome graph

The pangenome graph was constructed using the latest ARS-UCD1.2 bovine reference assembly as backbone, with the 64 de novo genome assemblies being aligned iteratively based on their phylogenetic relationships. Firstly, we assessed the phylogenetic distance between samples using the Mash software (v2.3) [24]. Parameters were set to their default values. However, given the large size of cattle genomes, we established a sketch size of 100,000,000 using the “-s” option. The resulting distance matrix was used to build a phylogenetic tree with the ape library [25] in R (4.3.1), and the tree was visualized with the plot.phylo function [26].

Subsequently, we constructed whole-genome pangenome graph using Minigraph (v0.21), with the “-cxggs” parameter to perform base-alignment. The constructed pangenome graph consisted of a series of bubbles, with the reference genome serving as the primary path. Finally, we visualized the pangenome graph through BandageNG (v2022.09) [27].

### SV calling

Pangenome graph SVs are depicted as bubbles, where each bubble represents sequence variations across genome assemblies. We identified the allele present in each bubble for all samples by individually realigning the 64 assemblies to the pangenome graph using the “-cxasm --call” option of Minigraph. Node labelling was then generated along each genome’s alignment path, and the allele information for each sample was stored in a corresponding bed file. The 64 individual bed files were subsequently combined into a single VCF file following the Minigraph-cookbook-v1 guidelines [28]. Briefly, we first used the k8 tool alongside with the mgutils.js script to create a detailed bed file that encompassed all identified alleles across individuals. Subsequently, the bed file was converted into VCF format using the mgutils-es6.js script with the merge2vcf -r0 option.

Each SV was subsequently classified based on two distinct criteria: number of alleles and SV type. Firstly, SVs were considered as biallelic if the bubble contained exactly two paths (reference and alternative), and as multi-allelic if more than two paths were present. Secondly, SVs were classified as insertions when the reference path contained no sequence (length = 0) and the alternative path contained an insertion sequence of at least 50 nucleotides, and as deletions when the alternative path contained no sequence while the reference path retained a sequence of at least 50 nucleotides. All remaining SVs, where both the reference and the alternative (non-reference) paths contained genomic sequences, have been considered as substitutions.

### Extraction of non-reference unique insertion sequences (NRUIs)

Finally, we extracted non-reference sequences (NRSs) by applying the following three filters: (1) all nodes that did not successfully occur in any of the individual paths were classified as nested nodes and were excluded from further analysis—this is due to the observation that the realignment of assemblies onto the Minigraph pangenome graph can sometimes fail to efficiently determine the nodes that were originally constructed from their own respective sequences, particularly within complex bubbles containing a large number of nodes, these observations have been previously reported by Leonard et al*.* [13] and Miao et al*.* [29]; (2) all nodes that occurred in the reference path were also excluded; and (3) all nodes that passed the first 2 filters and have a sequence length higher than 50 bases where selected and where considered as non-reference sequences. We also extracted NRUIs by identifying only true insertion-type SVs (representing sequences absent from the ARS-UCD1.2 reference genome). Nodes corresponding to these insertions were selected, and sequences exceeding 50 bp in length were retained and classified as NRUI sequences.

### Validation of SVs by high-throughput genotyping

To evaluate the efficiency and population-level relevance of our SV detection approach, we applied a previously developed high-throughput genotyping strategy based on the bovine Illumina EuroGMD SNP array [30]. In our study, we focused only on deletions and we applied our previously reported method to convert deletions into virtual SNPs and add these to the SNP chip [31]. Briefly, the predicted deletions were converted into “virtual SNPs” by testing the base change at the SV breakpoints as follows: if the first nucleotide of the deleted region was different from the first nucleotide which was located immediately after the SV 3’ breakpoint, then the reference allele of the “virtual SNP” corresponds to the first nucleotide of the deleted region and the alternative allele corresponded to the first nucleotide immediately after the SV 3’ breakpoint. This genotyping can be confirmed by performing a complementary test on the opposite strand of the DNA. This cost-effective method enables the genotyping of multiple SVs across large populations and, using this approach, we compiled the genotyping database used for genomic selection for 230 deletions, relative to the Hereford reference genome assembly, across 2,838,235 animals from 21 French dairy and beef cattle breeds (Table 2).

**Table 2:** Number of animals genotyped for the 230 structural variants

Breed	Number of genotyped animals	Effective size (Cervantes method) [32, 33]
Abondance	26,970	56
Aubrac	11,580	448
Bazadaise	133	150
Blonde d’Aquitaine	33,658	241
Bretonne Pie Noire	57	81
Brown Swiss	78,632	95
Charolaise	80,880	658
Holstein	1,593,213	95
Inra 95	549	–*
Jersey	12,179	118
Limousine	18,742	674
Montbéliarde	715,854	86
Normande	218,455	93
Parthenaise	5630	271
Rouge des Prés	560	268
Rouge Flamande	345	78
Salers	5472	393
Simmental	15,653	170
Tarentaise	13,891	69
Vosgienne	5747	68
Other cattle breeds	35	–*
Total	2,838,235	–

^*^Effective sizes were not available for these breeds

As this panel of 230 deletions was initially identified from SR sequencing data [31], we used the SV catalog generated from the pangenome approach to validate their genomic positions. Additionally, we aimed to highlight the relevance of including SVs in GWAS analyses. While pangenomes are powerful tools for building SV panels, they are typically constructed from a limited number of assemblies and often lack associated phenotypic data. By combining SV genotyping with SNPs arrays and GWAS, we were able to impute these 230 deletions in a large population and associate them with phenotypic traits.

Genotyping data for these 230 deletions were used to estimated their allele frequencies both at the global population level and within each breed (see Additional file 2, Table S2). We also evaluated two standard population genetic metrics to assess the informativeness of these deletions. Firstly, we calculated the heterozygosity ratio (He), defined as He = 2pq, where *p* and *q* are the frequencies of the two alleles. Secondly, we computed the Polymorphic Information Content (PIC) score, which is given by the formula PIC = He−2p^2^q^2^ [34]. The PIC score measures the ability of a marker in detecting genetic polymorphisms of interest in linkage analysis.

### Analysis of population structure

To characterize the distribution of SVs and NRUIs across cattle breeds, we firstly conducted a population structure analysis based on the presence/absence variation (PAV) of SVs and NRUIs. For this purpose, we generated two binary PAV-matrices, each recording the presence or absence of either SV or NRUI for all individuals. Hierarchical clustering was then performed on the SV and NRUI PAV-matrices using the HCLUST function in R [35], enabling the visualization of breed clustering patterns based on SVs and NRUIs.

To further evaluate the reliability of population structure inferred from SVs, we used the genotyping data of the 230 SVs to carry out a Principal Components Analysis (PCA) using the “dudi.pca” function implemented in the R package ade4 [36]. This PCA was based on validated SVs genotypes from a panel of 1500 bulls from the three main dairy breeds (Holstein (HOL), Montbéliarde (MON) and Normande (NMD)) used in our GWAS studies. These validated SVs correspond to the SVs shared between the pangenome and EuroGMD genotyping array. The population structure inferred from SVs was then compared to previously reported results based on 50 k SNP data [37], allowing us to assess the consistency of SV-based clustering.

### Genome-wide association analyses

To assess the potential effects of the identified SVs on dairy cow performances, we conducted genome-wide association studies (GWAS) using the EuroGMD genotyping chip, which included the panel of 230 SVs detected through our pangenome-based approach.

The phenotypes used in GWAS were daughter yield deviations (DYD), defined as the average value of daughters’ performances, adjusted for systematic environmental effects and for the breeding value of their mates [38]. Bulls with records from at least 20 daughters with available phenotypes were included in the analysis.

#### Imputation of structural variants genotypes

**Fig. 1:**
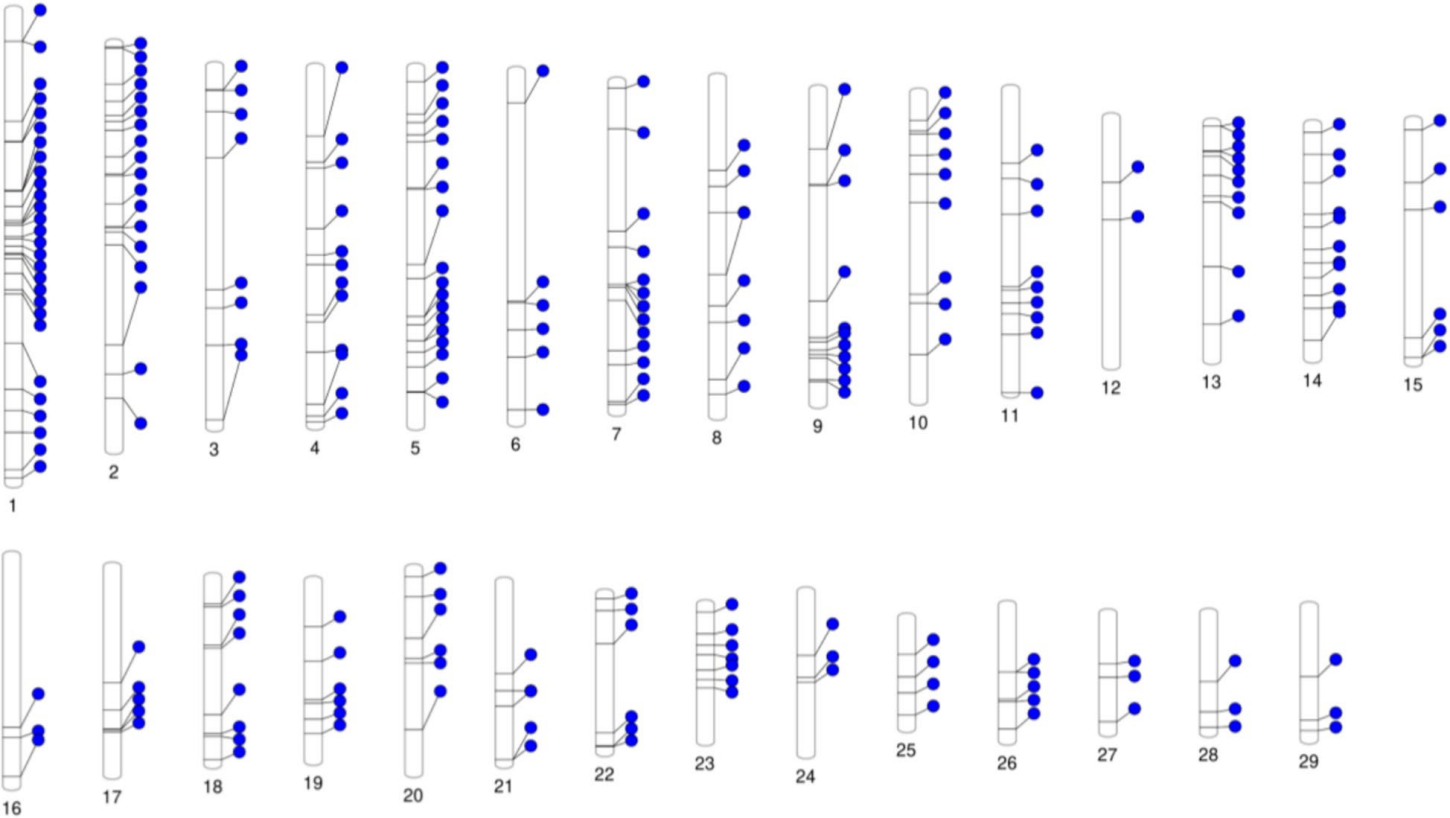
Ideogram of the distribution of the 230 structural variants present on the EuroGMD chip across all bovine autosomes. *SVs* Structural variants

SVs have been included since version 2 of the EuroGMD array. Specifically, 182 SVs are present in both v2 and v3, with an additional 48 included in v4, bringing the total to 230 SVs. These SVs are distributed across the entire genome but in a non-uniform manner, with only 2 SVs on BTA12 (*Bos taurus* autosome) and 28 SVs on BTA1 (Fig. 1). As the bulls had been genotyped using different versions of the EuroGMD chip, some of which did not initially contain the 230 SVs, we imputed the missing SV genotypes, as described below, across the study population before conducting the association analyses. Following imputation, GWAS analyses were performed to investigate the associations between SV genotypes and key production and functional traits in dairy cattle.

Genotypes for the 230 SVs were available for a subset of animals genotyped using different versions of the EuroGMD that included these SVs. These data were used to establish breed-specific reference populations for the three main French breeds under study (HOL, MON and NMD). To maximise the accuracy of the imputed genotypes, the selection of animals for the reference populations was guided by two main criteria: (i) the inclusion of widely used artificial insemination (AI) bulls in France, and (ii) the selection of animals closely related to the bulls targeted for imputation based on pedigree data for the HOL, MON and NMD breeds.

Imputation was performed using the FImpute software [39]. Each reference population was designed to include approximately 20,000 animals. To refine the selection, filters were applied based on the number of SVs genotyped per animal. Depending on breed-specific availability, a threshold was set at 180 out of 182 SVs and at 228 out of 230 SVs for EuroGMD v2, v3, and EuroGMD v4, respectively, in MON and HOL. For the NMD breed, the thresholds were set at 170 out of 182 SVs and 220 out of 230 SVs for EuroGMD v2, v3, and EuroGMD v4, respectively. After applying these filters, sires and dams were selected for all chip versions. Where possible, at least one descendant per bull from the GWAS population was added to the reference population to ensure broad genetic diversity, which was progressively expanded to approximately 20,000 animals.

#### Genome wide association study

We evaluated the effect of the 230 SVs panel in the three main dairy breeds (HOL, MON and NMD) for the following 13 traits:

- Five fertility traits: heifer conception rate (HCR), cow conception rate (CCR), calving—first artificial insemination interval (ICAI1), heifer non-return rate (HNRR) and cow non-return rate (CNRR);
- Five milk production traits: milk yield (MY), fat content (FC), protein content (PC), fat yield (FY) and protein yield (PY);
- Two udder health traits: somatic cell score (SCS)—was defined as SCS = 3 + log_2_(SCC/100,000) and calculated as the average of monthly records within each lactation, where SCC corresponds to the somatic cell count (cells/mL of milk)—and clinical mastitis (CM)—was defined within each lactation as a binary trait (0/1), with 1 indicating the occurrence of at least one clinical case before 150 days in milk;
- One morphology trait: height at sacrum (HS)

Comprehensive definitions and trait characteristics can be found on the Interbull website [40]. Depending on the trait, between 3096 and 3672 bulls, 2417 and 2986 bulls, and 9822 and 11,428 bulls with DYDs were used for the association analyses (Table 3) for the MON, NMD, and HOL breeds, respectively.

**Table 3:** Number of MON, NMD, and HOL bulls with daughters’ performance for each trait

Type of trait		MON	NMD	HOL
Fertility	Heifer conception rate (HCR)	3394	2764	10,950
	Cow conception rate (CCR)	3445	2882	10,978
	Calving-First insemination Interval (days) (ICAI1)	3508	2889	11,075
	Heifer non-return rate (HNRR)	3424	2771	10,924
	Cow non-return rate (CNRR)	3489	2892	10,953
Milk production	Milk yield (kg) (MY)	3672	2979	11,423
	Fat content (g/kg) (FC)	3672	2979	11,422
	Protein content (g/kg) (PC)	3672	2979	11,422
	Fat yield (kg) (FY)	3672	2979	11,420
	Protein yield (kg) (PY)	3672	2979	11,420
Udder health	Somatic cell score (SCS)	3620	2986	11,448
	Clinical mastitis (CM)	3096	2417	9822
Morphology	Height at sacrum (HS)	3599	2720	10,609

We performed GWAS with 58,191 genomic variants, including 230 SVs, for each breed separately, analysing one trait at a time. We used the *mlma* (Mixed Linear Model Analysis) approach implemented in the GCTA software. This method applies a mixed linear model including the variant to be tested presented in Eq. (1).

1 $$\mathbf{y}=1\upmu +\mathbf{x}\upbeta +\mathbf{u}+\mathbf{e}$$

where **y** represents the vector of DYD; μ is the overall mean; β is the fixed additive effect of the variant being tested for association; **x** is the vector of imputed genotypes, coded as the number of copies of the tested allele (0/1/2); $\mathbf{u} \sim N\left(0, \mathbf{G}{\upsigma }_{\text{u}}^{2}\right)$ is the vector of random polygenic effects, where **G** is the genomic relationship matrix (GRM) derived from 50 k SNP genotypes, and ${\upsigma }_{\text{u}}^{2}$ is the polygenic variance, estimated from the null model $\left(\mathbf{y}= 1\upmu +\mathbf{u}+\mathbf{e}\right)$, and then held fixed while testing the association between each variant and the trait of interest; finally, $\mathbf{e} \sim N\left(0,{\mathbf{D}\upsigma }_{\text{e}}^{2}\right)$ is the vector of random residual effects, with ${\upsigma }_{\text{e}}^{2}$ the residual variance. **D** is a diagonal matrix with inverse weights for DYD to account for heterogeneous accuracy.

A genome-wide significance threshold was applied using the Bonferroni correction, calculated as *P*-value = 0.05 / 58,191, where 0.05 is the nominal significance level and 58,191 is the number of variants analysed. This resulted in a significance threshold of *P*-value = 8.59 × 10^–7^, corresponding to −log_10_(*P*-value) = 6.07, rounded to 6.1.

#### QTL identification

To determine the number of QTL regions associated with each phenotype, we applied an iterative procedure as described in Sanchez et al*.* [41], for each breed separately. Briefly, this procedure aims at (i) defining confidence intervals (CIs) for QTL based on LD between SNPs and (ii) identifying putative multiple QTLs within a given region. The procedure was applied in 10 Mb windows, grouping SNPs into the same QTL region if they exhibited LD (R^2^) greater than 0.7 with the most significant SNP.

In the identified QTL regions, most of the significant SVs were selected to precisely determine their genomic localization. Therefore, breakpoint coordinates of each SV were extracted and mapped its position onto the reference genome. To functionally annotate these SVs, whether they directly affected a gene or were located near gene regulatory regions was checked, using the Ensembl [42] and UCSC [43] databases. This approach follows the methodology described by Boussaha et al*.* [31] and Letaief et al*.* [44].

### Visualisation of SVs with the pangenome graph

In QTL regions where an SV showed a significant effect on the phenotype, we validated the functional impact of the SV by constructing a local pangenome over a 2 Mb region surrounding the locus. To achieve this, the sequences of each animal from the panel of 64 CLR assemblies corresponding to the ARS-UCD1.2 reference genome coordinates were extracted following three main steps: (i) aligning genomes against the reference using minimap2 (v2-2.28) [45], (ii) extracting the region of interest using IMplicite Pangenome Graph (IMPG) (v0.2.1) [46], and (iii) obtaining the corresponding FASTA sequences using samtools faidx (v1.20) [47]. Finally, a pangenome was built using Minigraph as described previously and visualized the region on the graph using BandageNG [27].

### Validation by PCR and Sanger sequencing of SVs

**Fig. 2:**
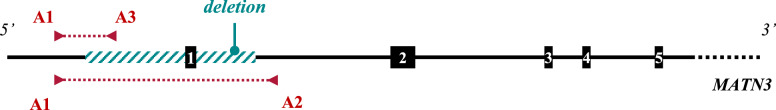
PCR-based validation of *MATN3* deletion. Structure of the *MATN3* gene. Black boxes and lines represent first 5 exons and introns, respectively. Primers pairs A1-A2, A1-A3 are indicated in red. The blue hatched box represents the deletion

Targeted SV breakpoints were validated by PCR amplification and Sanger sequencing. In this study, we focused only on the validation of a deletion located within the *MATN3* gene, which was found to be significantly associated with stature in Holstein. Six animals were used in the validation step: two heterozygotes, two homozygotes for the deletion, and two non-carrier animals. Primers were produced by Eurofins Genomics (Ebersberg, Germany). We designed these primers to amplify two distinct amplicons (Table 4), allowing the detection of presence or absence of the deletion. The first amplicon, which was 422 bp in size, was amplified using the A1-A3 primer pair and identified homozygous individuals for the reference allele (*i.e.*, non-carriers of the deletion). The second amplicon, obtained with the A1-A2 primers flanking the deletion breakpoints, measured 580 bp and identified individuals carrying the deletion, corresponding to the alternative allele (Fig. 2). The size difference between these two fragments allowed the distinction of heterozygous individuals, which displayed both amplicons at the same time.

**Table 4:** Features of PCR primers

Name	Orientation	Sequence (5′ 3′)	Amplicon size (nucleotides)
A1	Forward	TGCTTGAGCTTGGGGTACTTT	580
A2	Reverse	GTTACAGGGGTGTAGTGGGC	
A1	Forward	TGCTTGAGCTTGGGGTACTTT	422
A3	Reverse	CTGCTGGGGTGGGAAATCTG	

See Fig. 2 for details about primer pairs usage

PCR was performed using 100 ng of DNA in a 35 µL reaction mixture consisting of 1X GoTaq Flexi Buffer [48], 2.5 mM MgCl_2_, 800 µM dNTPs, 0.875 UI GoTaq DNA polymerase [48], and 0.5 µM of each primer (A1, A2, and A3). The PCR program consisted of an initial denaturation step at 94 °C for 3 min, followed by 35 three-step cycles: (i) denaturation at 94 °C for 30 s, (ii) annealing at 67 °C for 30 s, and (iii) extension at 72 °C for 1 min, and a final extension step at 72 °C for 5 min before cooling to 15 °C. PCR products were visualized by gel electrophoresis on a 2% agarose gel. Additionally, PCR products were sequenced by Eurofins Genomics (Ebersberg, Germany) to determine the precise breakpoint sequences of the studied SVs.

## Results

### Quality of the 64 de novo genome assemblies

The current ARS-UCD1.2 reference assembly metrics are: 2,759,153,975 bases total size including unmapped contigs, 25.9 Mb N50 contig and 95.8% BUSCO scores. To assess the quality of our final panel of 64 polished de novo assemblies, we computed the genome length, N50 contig metrics, and BUSCO scores (see Additional file 1, Table S1). The 64 de novo genome assemblies presented an average genome length of 2,636,580,836 bases, ranging from 2,612,227,180 to 2,688,017,017 bases. The average N50 contig length was approximately 12 Mb, with values ranging from 4.1 to 26.2 Mb and an average L50 scaffold count of around 12. These N50 and low L50 scores suggested that the genome assemblies were contiguous and exhibit minimal fragmentation. The average BUSCO score was 95.1%, with individual scores ranging from 93.9 to 95.7%. These high BUSCO values reflected high genome completeness. Furthermore, alignment of these de novo assemblies with D-Genies [49] (see Additional file 3, Figures S1 to S14) showed a high degree of similarity and concordance with the chromosomes of the ARS-UCD1.2 reference genome assembly.

Collectively, these quality metrics confirm that all 64 genome assemblies were of sufficient quality and suitable for constructing a cattle pangenome graph.

### Characterization of the cattle pangenome graph

**Fig. 3:**
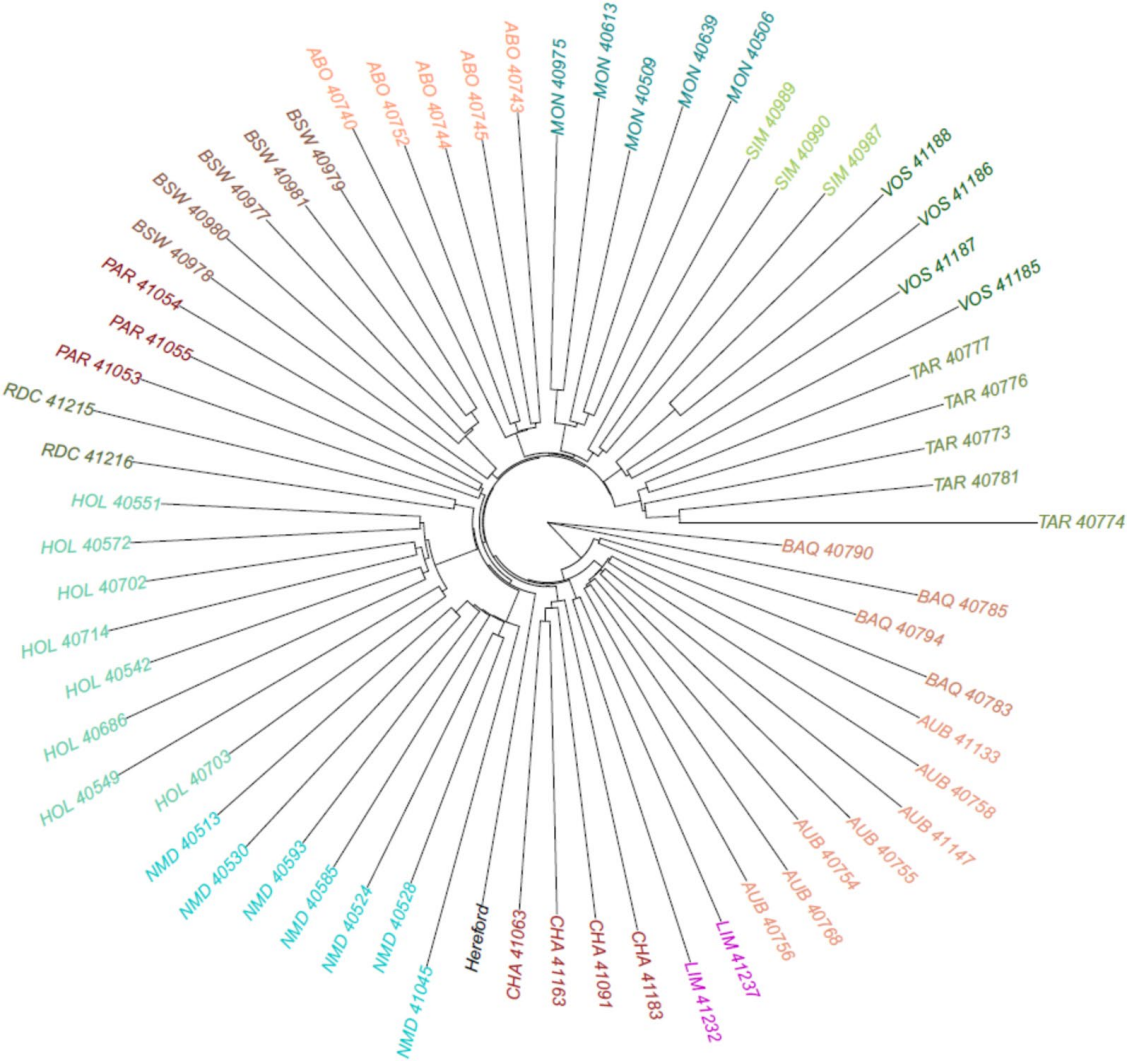
Phylogenetic distance between the 64 genome assemblies and ARS-UCD1.2. Phylogenetic tree derived from 64 bovine assemblies and the current Hereford reference genome assembly

We constructed a cattle pangenome graph using the 64 de novo assemblies, with the ARS-UCD1.2 reference genome sequence used as backbone. Firstly, we estimated phylogenetic distances between assemblies using Mash and clustered samples into 14 groups according to their breed of origin (Fig. 3). These distances were subsequently used to construct a phylogenetic tree. The resulting pangenome graph consisted of 521,756 nodes connected by 735,034 edges, representing a total sequence length of 2,933,608,906 bases. Notably, 5.95% of the graph (174,454,931 bases) corresponded to sequences missing from the ARS-UCD1.2 bovine reference genome assembly. To ensure the accuracy of node labelling, we realigned each individual genome assembly to the graph and traced the corresponding path for each sample. A total of 507,822 out of the 521,756 nodes were successfully linked into the paths, covering 2,858,048,002 bases. The remaining 13,935 nodes, representing 75,560,904 bases, did not occur in any genome. They were considered as nested nodes and were therefore excluded from further analysis.

**Fig. 4:**
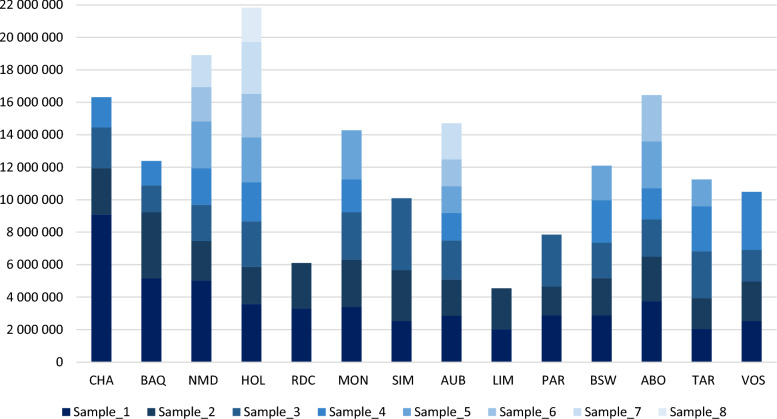
Number of bases added in the flexible genome per sample and per breed. The number of samples per breed ranged from 2 to 8

We subsequently assessed the contribution of novel sequences from each assembly to the construction of the pangenome graph (Fig. 4). Overall, the first individual used to construct the graph tends to provide the largest amount of novel sequence within each breed. Subsequently, the first individual from each breed, in the order of integration, generally contribute decreasing amounts of new sequence to the pangenome. Five out of the 14 breeds (*i.e*. PAR, BSW, ABO, TAR, VOS) used in this study, provided more diversity. This can be explained by the fact that those 5 breeds are genetically more distant from the other ones.

The core pangenome (nodes shared by the 64 assemblies and Hereford reference genome assembly) contained 112,525 nodes, corresponding to 2,562,959,040 bases (90% of the graph). On the other hand, flexible pangenome regions contained 395,298 nodes, with a cumulative sequence length of 295,088,962 bases (10%). Within the flexible regions, 83,050 nodes containing 99,187,939 bases were identified as breed-specific (Table 5). Additionally, we identified 151,771 nodes with a cumulated sequence length of 158,701,735 bases that passed our filtering criteria and were therefore classified as non-reference nodes. We further analysed the 158.7 Mb of novel sequences (referred as NRSs) by focusing only on true insertions that originated from bubbles with no reference nodes. This analysis revealed 27,550 non-reference nodes, representing a total sequence length of 25,470,897 bases of true and good quality NRUIs. Notably, 9915 of these nodes, containing 13,344,651 bases, were identified as breed-specific, appearing exclusively in one breed and absent in all the other breeds (a node could be carried by just one animal within that breed) (Table 5).

**Table 5:** Distribution per breed of flexible sequences and non-reference unique insertions

Breed	Number of assemblies	Number of flexible nodes	Total length of flexible sequences (nucleotides)	Number of breed-specific NRUIs nodes	Total length of breed-specific NRUIs (nucleotides)
Hereford	1	1719	8,747,369	0	0
Abondance	5	7388	8,930,960	717	790,073
Aubrac	7	7143	8,249,769	1064	1,559,693
Blonde d’Aquitaine	4	3847	4,228,801	528	866,336
Brown Swiss	5	6819	7,693,269	839	1,158,611
Charolaise	4	4349	4,947,248	640	948,814
Holstein	8	9137	10,685,671	1168	1,732,578
Limousine	2	2232	2,255,173	334	419,269
Montbéliarde	5	6353	7,332,347	739	921,838
Normande	7	8088	8,851,454	1060	1,240,542
Parthenaise	3	4638	4,765,273	557	648,038
Rouge Flamande	2	1838	1,780,683	4260	615,828
Simmental	3	5560	6,472,286	434	659,700
Tarentaise	5	5495	6,854,827	724	1,084,256
Vosgienne	4	8444	7,392,809	691	699,075
Total	65	83,050	99,187,939	9,915	13,344,651

**Fig. 5:**
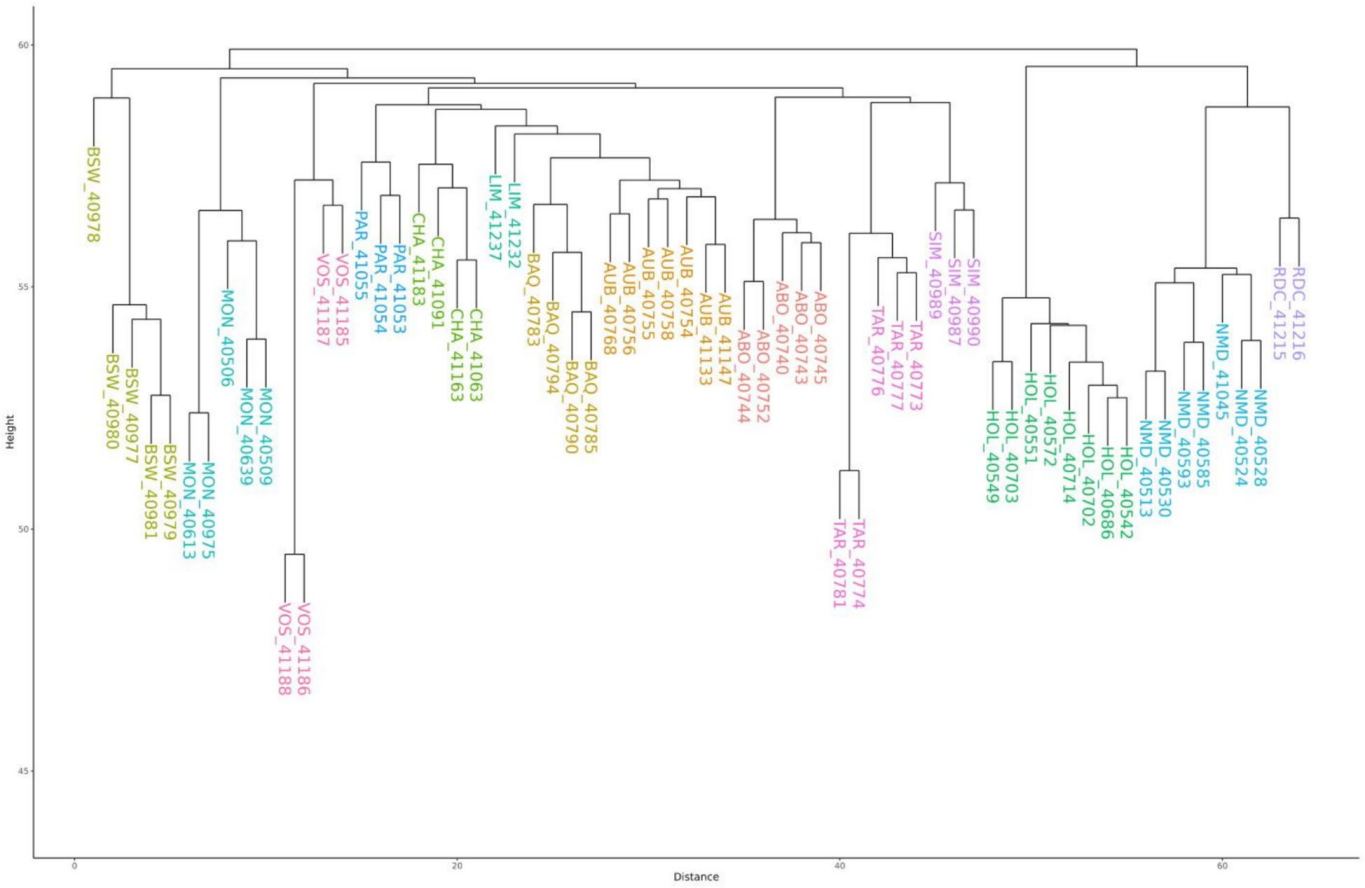
Hierarchical clustering of the 64 assemblies based on the NRUI PAV-matrix. *PAV* presence/absence variation; *NRUIs* non-reference unique insertions; Diagram showing the clustering of the 64 assemblies according to the 14 breeds for the 9247 NRUIs

Hierarchical clustering based on presence/absence variation (PAV) matrix of NRUIs (Fig. 5) successfully grouped samples according to their breed of origin, highlighting the strong association between these unique insertions and breed structure.

### Display SVs identified from the graph

In total, we identified 109,275 SVs (Table 6). Out of these, 77.4% (84,612 SVs) were classified as biallelic. Among the biallelic SVs, 61.2% were further categorized into 27,171 insertions (52.5%) and 24,592 deletions (47.5%). The remaining SVs corresponded to bubble that contained sequences in both the reference and non-reference paths, and were therefore classified as sequence substitutions. We analysed the length distribution of biallelic insertions and deletions (see Additional file 3, Figures S15 to S28) and observed a symmetric distribution between the sizes of SVs classified as insertions and those classified as deletions. Moreover, we identified several notable peaks at 150 bp, 250 bp, 5.5 kb and 8.6 kb, which likely correspond to structural variations associated with different families of transposable elements (SINE, LTR, LINE).

**Table 6:** Distribution of SVs by allele count and SV type

Mutations	Bi-allelic count		Multi-allelic count	Total
Insertions	27,171	21,840*	1997	29,168
		5331**		
Deletions	24,592	21,340*	3435	28,027
		3252**		
Others mutations***	32,849		19,231	52,080
Total	84,612		24,663	109,275

^*^Alternative/reference allele length = 0; ** alternative/reference allele length comprised between 1 and 5; ***Others mutations corresponded to inversions, duplications, alternate insertions (both reference and non-reference sequences were present but non-reference allele is longer) and alternate deletions (same as for insertions, but non-reference allele is shorter)

**Fig. 6:**
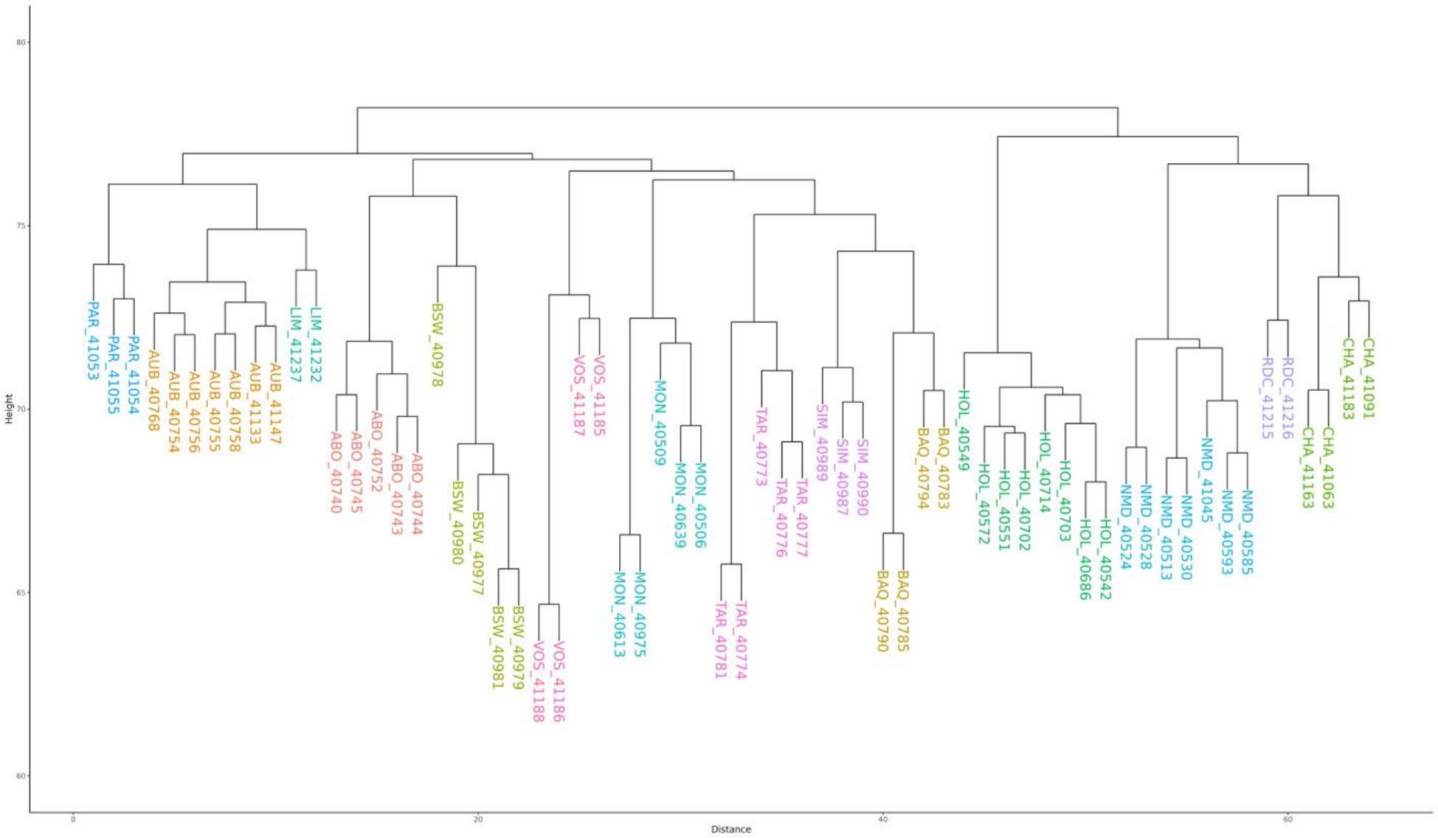
Hierarchical clustering of the 64 assemblies based on the SV PAV-matrix. *PAV* presence/absence variation; *SVs* structural variants; Diagram showing the clustering of the 64 assemblies according to the 14 breeds for the 14,929 SVs

Similar to results observed with NRUIs, hierarchical clustering based on PAV-matrix of SVs (Fig. 6) correctly assigned all samples to their corresponding breed of origin.

### Validation of SVs by SNP chip genotyping

Relevance of pangenome-derived SVs was assessed using the EuroGMD SNP chip genotyping data of 230 selected SVs obtained for a large population of animals representing 21 cattle breeds (see Additional file 5, Table S3). All SVs were successfully genotyped, of which 221 were polymorphic. The 9 monomorphic SVs were likely to be either false positives or SVs specific to the Hereford reference genome assembly.

Mean observed minor allele frequency (MAF) across polymorphic SVs was 0.17 at the population level, ranging from 0.14 in Salers up to 0.21 in Bretonne Pie Noire (see Additional file 5, Table S3). The mean observed heterozygosity across loci was 0.24, ranging from 0.20 in Salers to 0.29 in Bretonne Pie Noire. The mean PIC (Polymorphic Information Content) was 0.19 and varied from 0.17 in Salers to 0.23 in Bretonne Pie Noire (see Additional file 5, Table S3). Heterozygosity and PIC are key parameters to assess the informativeness of genetic markers. Based on the values observed in our SV panel across the 21 breeds, these markers can be considered informative and are particularly suitable for population structure analysis and association studies. Indeed, given that these SVs were bi-allelic, the highest possible values for H_e_ and PIC are 0.500 and 0.375, respectively. Also, the mean MAF at the population level of our SV panel was 0.172 with observed H_e_ and PIC values of 0.242 and 0.198, respectively. Since SVs are individually less abundant than SNPs, we can hypothesize that SVs with a MAF higher than 10% are somewhat frequent at the population. Therefore, for SVs with a MAF ≥ 0.1, the observed H_e_ and PIC values were respectively 0.18 and 0.164 and can be considered very informative.

**Fig. 7:**
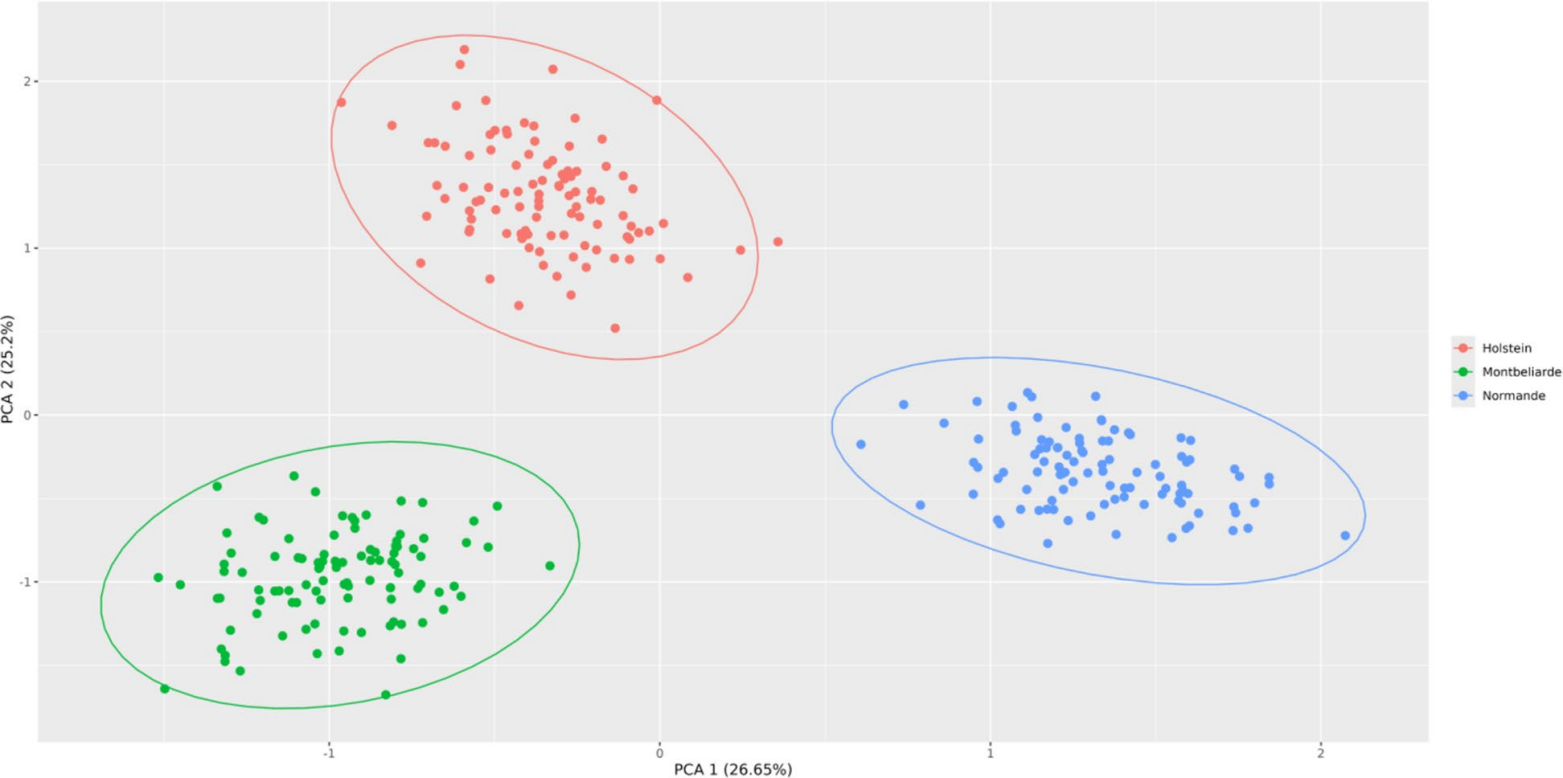
Results of PCA for the 3 mains French dairy breeds. Red dots correspond to Holstein animals, green dots correspond to Montbéliarde animals and blue dots correspond to Normande animals

To further assess the quality and informativeness of the validated SV panel, we investigated population structure using genotyping data from the three main French dairy breeds (MON, NMD and HOL). PCA accurately assigned all individuals to their breeds of origin (Fig. 7). These results provide additional statistical validation, complementing the validation of the SV panel.

### Genome wide association analyses

GWAS were performed for 13 traits related to milk production and composition, udder health, fertility, and stature, with 58,191 genomic variants, including 230 SVs. We detected a total of 49, 61, and 158 QTL with significant effects on various phenotypes (−log_10_(P) ≥ 6.1) in MON, NMD and HOL bulls’ panel, respectively (Table 7). Specifically, for MON, between 2 and 18 QTL were identified for milk production traits, 4 for stature, 2 for fertility traits and 1 for udder health traits. In the NMD breed, 3 to 19 QTL were found for milk production traits, 1 QTL for HCR, and 13 QTL for SH. Finally, in the HOL panel, from 1 to 48 QTL were found for each trait, except for CM. Overall, milk production traits exhibited the highest number of QTL across the three breeds, with 41, 47, and 130 QTL detected in MON, NMD, and HOL, respectively. In contrast, fewer QTL were found for traits related to fertility (3, 1 and 15, respectively) and udder health (1, 0 and 3, respectively) (see Additional file 6, Figures S29 to S41).

**Table 7:** Number of QTL identified per breed and phenotype, along with the corresponding number of significant SNPs and SVs

Type of trait		MON			NMD			HOL		
		#QTL	#SNP	#SV	#QTL	#SNP	#SV	#QTL	#SNP	#SV
Fertility	Heifer conception rate	0	–	–	1	5	–	3	4	–
	Cow conception rate	1	1	–	0	–	–	2	5	–
	Calving–1st AI Interval	2	8	–	0	–	–	8	29	–
	Heifer non–return rate	0	–	–	0	–	–	1	6	–
	Cow non–return rate	0	–	–	0	–	–	1	1	–
Milk production	Milk yield (kg)	3	17	–	4	9	–	14	94	–
	Fat content (%)	2	14	–	3	5	–	21	133	–
	Protein content (%)	2	2	–	4	8	–	5	8	–
	Fat yield (kg)	18	93	–	17	144	–	48	270	–
	Protein yield (kg)	16	70	–	19	120	–	42	213	–
Udder health	Somatic cell score	0	–	–	0	–	–	3	5	–
	Clinical mastitis	1	14	–	0	–	–	0	–	–
Morphology	Height at sacrum	4	31	–	13	68	–	10	28	1

*SNP* Single Nucleotide Polymorphism; *SV* Structural Variant; *MON* Montbéliarde; *NMD* Normande; *HOL* Holstein

QTL associated with milk production traits were identified on chromosomes 6 and 14 in all three breeds. Specifically, a peak was detected on BTA6 at approximately 85.5 Mb for PY (see Additional file 6, Figure S38). On BTA14, a peak was observed around 600 kb for MY and FC, and it was associated with the most significant effect for FY across the three breeds (see Additional file 6, Figures S34, S35, and S37). In contrast, QTL associated with fertility, udder health, and morphology traits were located on different chromosomes depending on the breed. For instance, for HCR, no QTL was detected in MON, but peaks were found on BTA19 in NMD, and on BTA6, 7, and 15 in HOL (see Additional file 6, Figure S29). Similarly, another fertility trait (*i.e.,* CCR), showed no significant association in NMD but displayed distinct peaks on BTA29 in MON and on BTA14 and 18 in HOL (see Additional file 6, Figure S30).

**Fig. 8:**
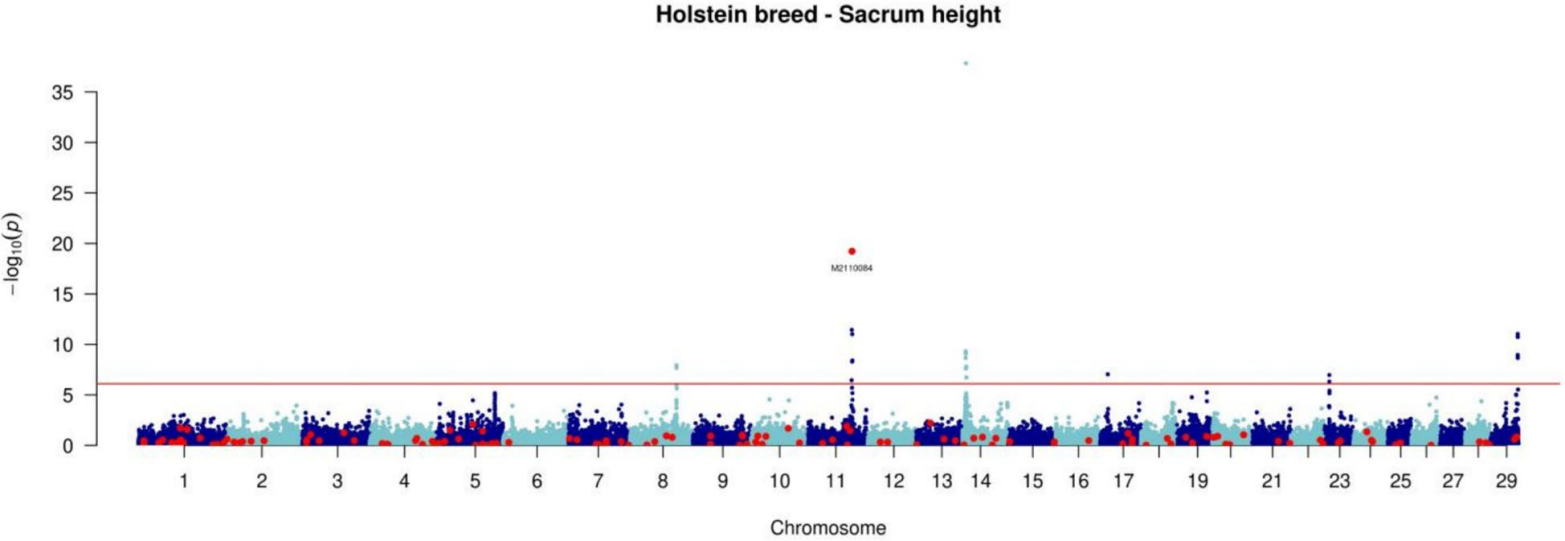
Manhattan plot of GWAS analysis: -log_10_(P) values plotted against the positions of *Bos taurus* autosomes for variants associated with height at sacrum in Holstein bulls. Red dots correspond to SVs alongside the genome, blue dots correspond to SNPs

Among genomic variants with significant effects on traits, we identified one SV presenting the second most significant association with height at sacrum in the HOL breed (−log_10_(P) = 19.22) (Fig. 8). This SV, specific to the Holstein breed, corresponds to a 6.2 kb deletion spanning the BTA11:78,819,207–78,825,389 region of the pangenome.

**Fig. 9:**
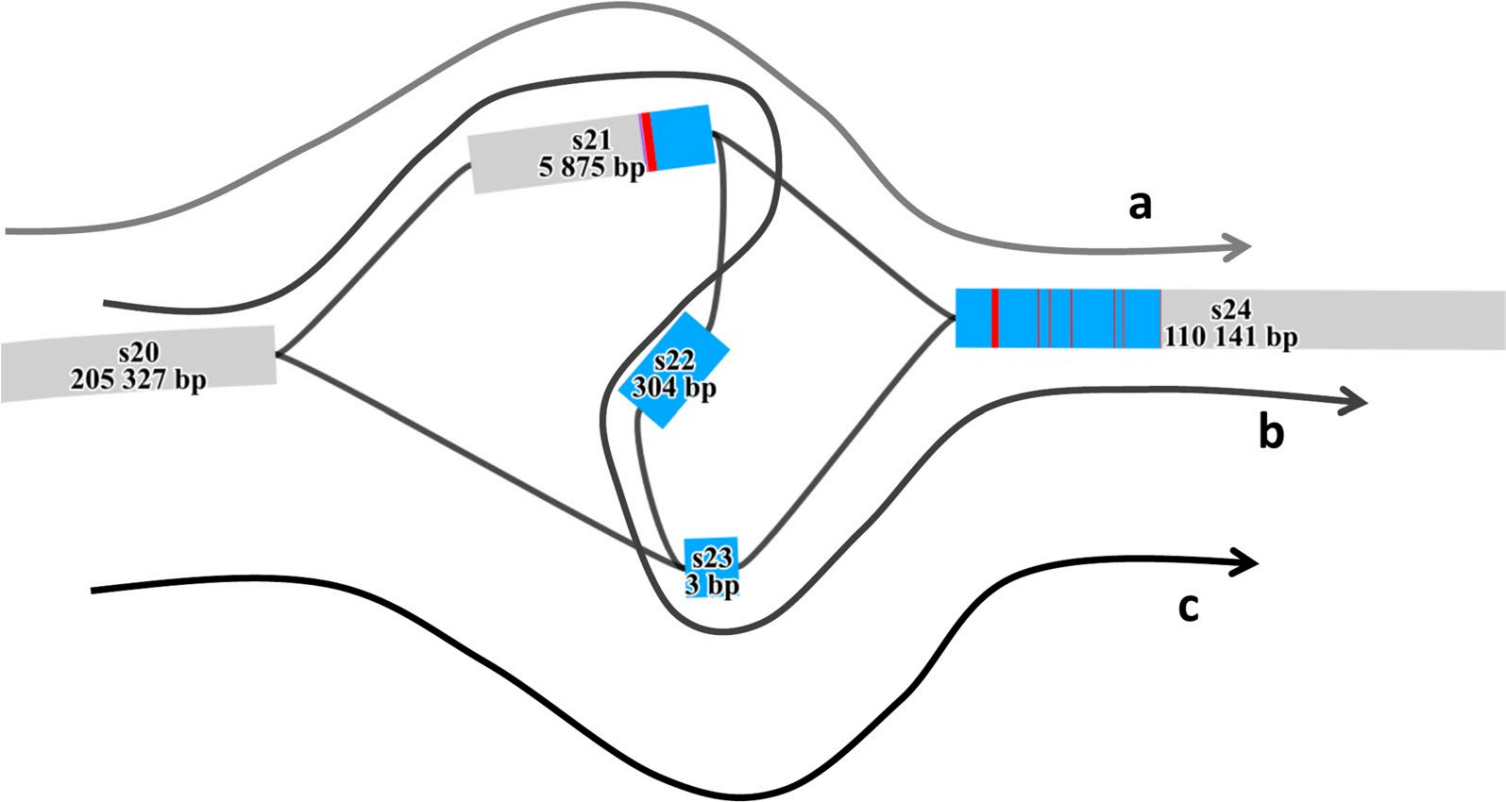
Local pangenome of the *MATN3* region. Alignment of *MATN3* exons (red bands) and introns (blue bands) on the local pangenome. Moreover, the three different paths identified in the assemblies are illustrated by the three arrows: **a** observed in one Holstein assembly, corresponding to an alternative 307 bp deletion; **b** corresponding to the reference allele, present for the ARS-UCD1.2 reference genome assembly and three Holstein individuals; and **c** observed in four Holstein individuals, corresponding to the 6.2 kb deletion

Analysis of a local pangenome graph within the 2 Mb region surrounding this structural variant revealed the presence of two major paths. The first corresponded to the reference allele, spanning nodes s20, s21, s22, s23, and s24 (Fig. 9a). The second path represented the alternative allele and included a 6.2 kb deletion, spanning nodes s20, s23, and s24 (Fig. 9b). This deletion overlaps with the *MATN3* gene, potentially disrupting its structure. Alignment of *MATN3* gene sequences revealed that the major part of the gene is located within the core pangenome (Fig. 9c). However, the first exon and part of the first intron of the gene are located within the nodes of the flexible genome, suggesting that the 6.2 kb deletion may affect the 5’ UTR regulatory region, as well as the first exon of the *MATN3* gene.

### MATN3 breakpoint validation

**Fig. 10:**
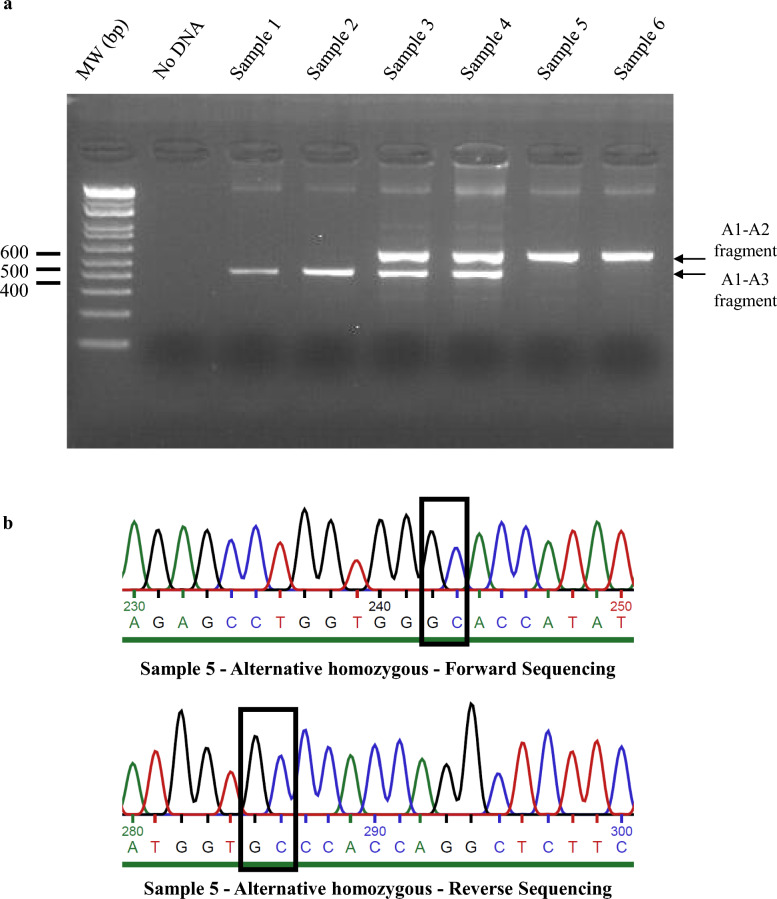
PCR validation of the *MATN3* deletion. **a** Gel electrophoresis of A1-A2-A3 PCR products obtained from six different samples. Samples 1 and 2 showed a product around 400 bp, indicating homozygosity for the reference allele. Samples 3 and 4 displayed two bands around 400 and 600 bp, indicating heterozygosity. Samples 5 and 6 presented a product around 600 bp, indicating homozygosity for the alternative allele. **b** Sanger sequencing results for sample 5 showing the breakpoints of the deletion (indicated by black boxes)

The *MATN3* deletion region, identified through GWAS and pangenome structural variant analysis, was amplified from genomic DNA in six individuals, *i.e.* two of each genotype. Gel electrophoresis confirmed the expected amplicon patterns: individuals 1 and 2 carried the reference allele (422 bp), individuals 5 and 6 carried the alternative allele (580 bp), and heterozygous individuals (samples 3 and 4) displayed both fragments (Fig. 10a). PCR products from one reference homozygote (sample 2) and the two alternative homozygotes were sequenced. Sanger sequencing validated the deletion breakpoints at positions 78,819,206 bp and 78,825,386 bp on chromosome 11 (based on ARS-UCD1.2 coordinates), occurring between nucleotides G and C (Fig. 10b).

## Discussion

The current ARS-UCD1.2 reference genome is a consensus assembly sequence from a single Hereford cow. It thus presents limitations to study the whole spectrum of cattle genetic diversity. In this study, we used 64 de novo genome assemblies for 14 dairy and beef cattle breeds to construct a cattle pangenome graph that more broadly represents the genetic diversity of French cattle breeds. To ensure the construction of a high-quality pangenome graph, we applied three extensive sequence polishing steps on both CLR long reads and high-quality Illumina short reads to correct for a large proportion of small assembly sequence errors. While reference-guided scaffolding may introduce potential biases in contig orientation and order, this concern is largely mitigated in this study by the high contiguity of the assemblies, with an average contig N50 of 12 Mb. Furthermore, current knowledge of bovine genome architecture suggests limited large-scale structural rearrangements, which supports the use of a reference-based approach in this context. While the total assembly sizes were slightly shorter than that of the ARS-UCD1.2 reference genome assembly, they remain highly comparable to previously published bovine genome assemblies [3]. This shorter size can be partially explained by the difficulty to assemble repetitive sequences with CLR sequencing, such as centromeric and telomeric repeats. Analyses using D-Genies [49] between assemblies and the reference genome assembly also confirm assemblies quality. Phylogenetic tree reconstruction further shows a clear clustering of individuals to their breed of origin.

Our pangenome graph provides new insights into the genetic diversity of the 14 French dairy and beef breeds used in this study and can be considered as a valuable resource for future genomic studies. To our knowledge, this is the first study of this scale in terms of diversity and number of *Bos taurus taurus* assemblies used, allowing the identification of several megabases of novel sequences not present in the ARS-UCD1.2 reference genome. Compared to the findings of Crysnanto et al*.* [9], our pangenome is characterized by a significantly higher number of nodes (507,822 vs. 182,940) and a larger total size (2,858,048,002 bases vs. 2,558,596,439 bases). These differences can be explained by the fact that our study, unlike the others, includes chromosome X in the graph construction. A second explanation is that we included a larger number of assemblies (64 vs. 6 individuals), as well as a more diverse set (14 vs. 6 breeds). This enabled us to capture more genomic variation and increase the structural complexity of the pangenome graph. Similar conclusions were reported by Miao et al*.* [29], who observed an increase in pangenome diversity when utilizing 21 pig assemblies compared to 11 in a previous study [50]. The increased heterogeneity of our assemblies also influences the distribution of sequences between the core and the flexible genomes. Specifically, 90% of the pangenome (2,562,959,040 bases) are conserved across all individuals, while the remaining 10% (295,088,962 bases) correspond to variable regions. This proportion of the flexible genome is higher than previously reported, where the core genome accounted for 93.9% (2,402,561,410 bases) and 95.8% (2,598,811,581 bases) of the total pangenome size, compared to 6.1% (156,035,029 bases) and 4.2% (109 Mb) of variable sequences, in Crysnanto et al*.* [9] and Leonard et al*.* [13] studies, respectively. However, the study by Dai et al*.* (2023) [51], which used 20 pseudo-phased HiFi assemblies from Chinese indicine cattle, reported a proportion of ~ 78% and ~ 22% for the core and flexible genome, respectively. This was higher than in our study, but this can likely be explained by the use of indicine animals that are genetically more distant from the ARS-UCD1.2 reference genome.

In total, we identified 151,771 nodes representing 158.7 Mb absent from the cattle reference genome. This exceeds the amount of NRSs reported in previous studies, which identified 70.3 Mb in *Bos taurus* [9], 18.6 Mb [52] in *Bos indicus*, 148.5 Mb [51] in Chinese indicine, 38.3 Mb in goats [53], and 72.5 Mb [54] and 105.16 Mb [29] in pigs, based on pangenome graphs constructed from 6, 5, 20, 8, 11, and 21 assemblies, respectively. However, our dataset includes a substantially larger number of individuals, which likely accounts for the higher amount of identified NRSs. These findings are consistent with the patterns observed in our pangenome graph, particularly regarding breed-specific NRUIs. Indeed, we observed a positive correlation between number of individuals per breed and total NRUI length. For instance, Holstein cattle (8 individuals) exhibited a total of 1.73 Mb of NRUIs, whereas Limousine and Rouge Flamande breeds (2 individuals each) showed lower values of 0.42 Mb and 0.62 Mb, respectively.

From the pangenome graph, we identified 109,275 SVs, which is higher than SV catalogs previously reported. For example, a catalog based on 16 HiFi cattle haplotype-resolved assemblies detected 53,297 SVs [55], and another study identified 68,328 SVs from six cattle assemblies [9]. In our SV catalog, 84,612 SVs (77.4%) were characterized as bi-allelic, with 27,171 insertions and 24,592 deletions. The distribution of insertion and deletion sizes revealed a symmetrical pattern between these two categories, with a majority of small size (between 50 and 200 bp) SVs, and a decreasing number of SVs as size increased. Additionally, four peaks (150 bp, 250 bp, 5.5 kb and 8.6 kb) were observed and corresponded to the SVs caused by transposable elements. These results are consistent with previous studies, such as those reported by Eché et al*.* [3]. Moreover, the proportion of bi-allelic SVs in our study (77.4%) is lower than the 94% (64,224 SVs) reported by Crysnanto et al*.* [9]. This difference can be attributed to the inclusion of a larger number of assemblies from diverse cattle breeds in our pangenome, which increases the likelihood that some SVs display more than two alleles, thus classifying them as multi-allelic. Further analysis of the insertions revealed 25.4 Mb of NRUIs, that are of particular interest as they may code for potential functional elements.

Inspection of this SV panel revealed the presence of several known SVs related to phenotypic traits. For example, we identified the 8.4 kb LINE1 insertion at the *ASIP* locus on BTA13 which encodes the AGOUTI signalling protein involved in mammalian pigmentation. This SV was found exclusively in all the Normande animals and is known to be associated with coat colour variation [56].

As nine out the 230 deletions were found monomorphic in our study, we used the genotyping data to investigate the effect of the 221 remaining polymorphic deletions by conducting GWAS analyses in the three main French dairy breeds for 13 phenotypes related to fertility, milk production, udder health, and morphology. We identified numerous QTL associated with the analysed phenotypes, all of them have been previously reported in other studies [57–59]. In most cases, candidate genes were highlighted, particularly for milk production and composition. Among them, the well-known *DGAT1* gene (*diacylglycerol O-acyltransferase 1*) that encodes an enzyme catalysing the synthesis of triglycerides in milk, was identified on BTA14 (~ 600 kb) [60, 61]. Similarly, the cluster of genes encoding αs1-casein (*CSN1S1*), αs2-casein (*CSN1S2*), β-casein (*CSN2*), and κ-casein (*CSN3*) was detected on BTA6 (~ 85.5 Mb) and is strongly associated with milk protein content [62, 63]. Additionally, *MGST1* (*microsomal glutathione S-transferase 1*), located on BTA5 (~ 93.5 Mb), has been found associated with milk fat content [64, 65]. However, identifying the causal mutation underlying a candidate gene remains a major challenge and is not systematically determined. To date, most GWAS have focused on SNPs or InDels, while SVs, which represent a substantial portion of genetic and phenotypic variability [55, 66], remain largely unexplored, particularly in cattle.

In this context, our study aimed to better characterize the impact of an SV panel on phenotypes of interest, providing new insights into their role in the genetic architecture of complex traits. Our results highlight a major QTL located on BTA11, associated with stature, where the most significant variant of the chromosome is an SV, *i.e.*, a 6.2 kb deletion located in the upstream region and the first exon of the *MATN3* gene. This finding is a promising advance toward incorporating SVs into GWAS. Its significance is further underscored by the fact that our study used only a subset of the SVs detected from the pangenome (230 deletions out of 84,612 bi-allelic SVs). This observation suggests that leveraging a more extensive SV panel could enhance the power to detect loci and potentially identify causal mutations underlying agronomically relevant phenotypes in GWAS.

Several studies have investigated the genetic determinism of stature in cattle, including a large-scale meta-analysis encompassing 17 cattle populations from nine countries [67]. This study identified 163 genomic regions of 1 Mb with significant effects on this phenotype, eight of which were located on chromosome 11. Among these, a SNP at position 78,870,305 bp (reference genome: UMD 3.1) exhibited the strongest association with stature (−log_10_(P) = 42.89) but no candidate gene was identified at this position. Our results confirm the involvement of this region in the genetic determinism of height at sacrum in cattle. The SV detected in our study is located at 78,825,400 bp, in close proximity to this region. Moreover, we identify the *MATN3* candidate gene that may be affected by the presence of this SV. Several other studies have also reported significant QTLs in this genomic region (BTA11 ~ 78 Mb), two mentioning the *MATN3* gene as a positional candidate gene [68, 69]. However, no functional validation has yet been conducted to confirm its biological role. Nonetheless, these findings further support our GWAS results and suggest a functional impact of this region, warranting further investigations. This also highlights that adding SVs into GWAS analyses can provide a better understanding of the genetic determinism of complex traits.

*MATN3* is part of the matrilin genes family and has been widely studied in recent years. This gene encodes the protein matrilin 3, which is primarily expressed in cartilage tissue and plays a key role in extracellular matrix assembly [70]. Due to its role in the collagen development, studies have shown that mutations in *MATN3* are associated with a predisposition to osteoarthritis and the premature development of growth plate chondrocytes in mice [71]. Additionally, a *MATN3* mutation has been associated to spondylo-epi-metaphyseal dysplasia and dwarfism in human [72].

In our study, the identified SV corresponds to a 6.2 kb deletion that affects the first exon and half of the first intron of *MATN3*. In cattle, no transcript isoforms is currently annotated in the Cattle Genotype-Tissue Expression atlas (CattleGTEx) [73] database, which is not unexpected given that *MATN3* is primarily expressed in cartilage during development – this combination of tissue and developmental stage is not well represented in existing expression datasets. To infer the most likely bovine transcript structure, we used the human transcript *MATN3* NM_002381.5 from NCBI as a reference. Comparative analysis of predicted cattle proteins with the human protein suggests that the most plausible bovine transcript corresponds to NCBI’s predicted transcript XM_015473571.2. Based on this transcript, the 6.2 kb deletion removes key regulatory elements upstream the gene, 5’UTR region, the full first exon (including the translational start site), and part of the first intron. Given this structure, it is likely that the deletion alters *MATN3* expression or partially disrupts the normal initiation of translation rather than causing a complete loss of function. Further studies are needed to better characterize the impact of this deletion on gene expression and protein function. The functional study in mice [71], mentioned above, supports the essential role of *MATN3* in skeletal development, but complete knockout in model organisms does not result in lethality.

Thus, in addition to characterizing the pangenome and identifying SVs and NRUIs in French cattle breeds, our study identifies a positional and functional candidate SV associated with stature in the Holstein breed. However, additional functional validation analyses are required to confirm the involvement of this variant in the genetic determinism of this trait.

## Conclusions

Numerous studies have underscored the value of pangenome graphs in identifying large structural variations often missed when relying on a single linear reference genome. Our findings corroborate these observations by using 64 de novo assemblies from 14 French dairy and beef cattle breeds to construct a comprehensive cattle pangenome. This approach enabled the identification of an extensive catalog of structural variations and non-reference sequences. By integrating some of these SV into GWAS analyses, we detected a 6.2 kb deletion in the *MATN3* gene, strongly associated with stature in Holstein cattle. This study emphasizes the importance of incorporating pangenome-based approaches in genetic studies to better capture variants that may contribute to key phenotypic traits in cattle.

## Supplementary Information

Supplememntary Material 1 Table S1. List of assemblies used in the studyDescription: List of metrics for all the assemblies used to construct the pangenome graph and the accession number of each sample.

Supplementary Material 2 Table S2. Minor Allele Frequency for imputed SVs in the three dairy cattle breeds. Description MAF calculated after imputation of the 231 SVs in the MON, NMD and HOL breed

Supplementary Material 3 Figures S1-S14 D-Genies plot for chromosomal alignment concordance between ARS-UCD1.2 on x-axis and the 64 assemblies on y-axis. Description: Plots were presented by breed in the following order: Abondance, Aubrac (2 pages), Blonde d’Aquitaine, Brown Swiss, Charolaise, Holstein (2 pages), Limousine, Montbéliarde, Normande (2 pages), Parthenaise, Rouge Flamande, Simmental, Tarentaise, and Vosgienne 2805 KB)

Supplementary Material 4 Figures S15-S28 Size distribution of SVs classified as deletions and insertions, identified using Minigraph, and SyRI for the 14 breeds. Description: Plots were presented by breed in the following order: Abondance, Aubrac, Blonde d’Aquitaine, Brown Swiss, Charolaise, Holstein, Limousine, Montbéliarde, Normande, Parthenaise, Rouge Flamande, Simmental, Tarentaise, and Vosgienne

Supplementary Material 5 Table S3 Frequencies of SVs identified in the pangenome and in the local database. Description: Estimation of allelic frequencies, Allele with the Minor Allele Frequency (al_maf), Minor Allele Frequency (freq_maf), Heterozygosity (He) estimated by He = 2pq and Polymorphic Information Content (PIC) estimated by PIC = He-2p2q2.

Supplementary Material 6 Figures S29-S41 Manhattan plots of each GWAS analyses for the three French main dairy breeds. Description: Plots were presented by phenotype analysed in the following order: heifer conception rate, cow conception rate, calving-first artificial insemination interval (AI1), heifer non-return rate, cow non-return rate, milk yield, fat content, protein content, fat yield, protein yield, somatic cell score, clinical mastitis, and height at sacrum

## Data Availability

The fasta files of the 64 assemblies used to build the pangenome are available in the European Nucleotide Archive (ENA) at EMBL-EBI under accession number PRJEB68295 (https://www.ebi.ac.uk/ena/data/view/PRJEB68295). Individual assembly accession numbers are available in supplementary file (see Additional file 1, Table S1). Additionally, paired-end Illumina SR data (2 × 150 bp) are available for the same 64 animals and publicly accessible under ENA project accession number PRJEB64023. The pangenome graph, 64 paths, the full VCF file and NRUIs extraction data are available at 10.57745/AI6Y4R.
