# Supplementary material for: Application of a French cattle pangenome, from structural variant discovery to association studies on key phenotypes
Source: Genet Sel Evol. 2025 Oct 23;57:61. doi: 10.1186/s12711-025-01012-x (PMC12551211; doi:10.1186/s12711-025-01012-x)
Supplement: Supplementary file 3 — Supplementary Material 3 Figures S1-S14 D-Genies plot for chromosomal alignment concordance between ARS-UCD1.2 on x-axis and the 64 assemblies on y-axis. Description: Plots were presented by breed in the following order: Abondance, Aubrac (2 pages), Blonde d’Aquitaine, Brown Swiss, Charolaise, Holstein (2 pages), Limousine, Montbéliarde, Normande (2 pages), Parthenaise, Rouge Flamande, Simmental, Tarentaise, and Vosgienne 2805 KB) [file 12711_2025_1012_MOESM3_ESM.pdf]

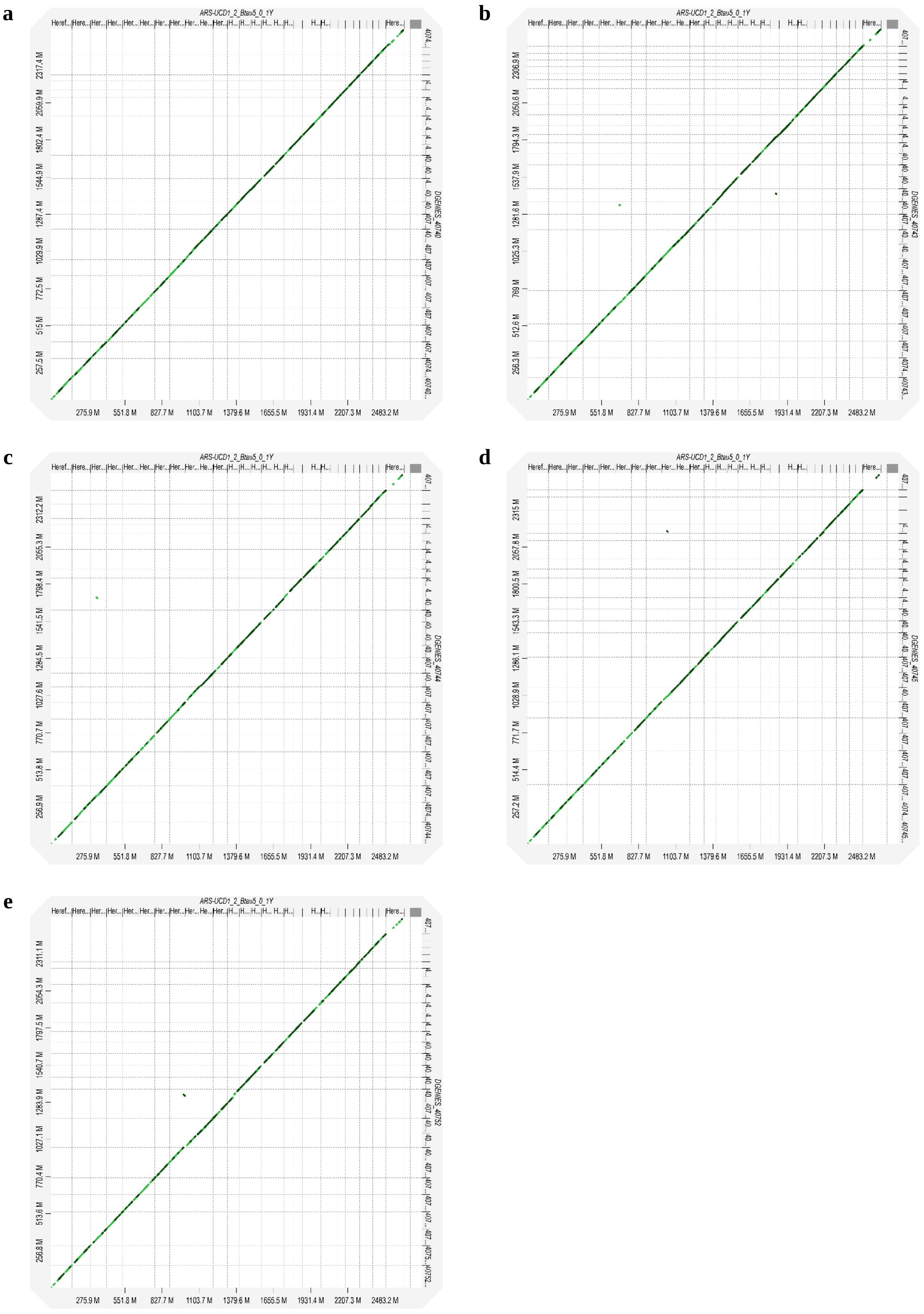

Figure S1 Chromosomal alignment concordance between ARS-UCD1.2 on *x*-axis and Abondance assemblies on *y*-axis: **a)** 40740, **b)** 40743, **c)** 40744, **d)** 40745, and **e)** 40752

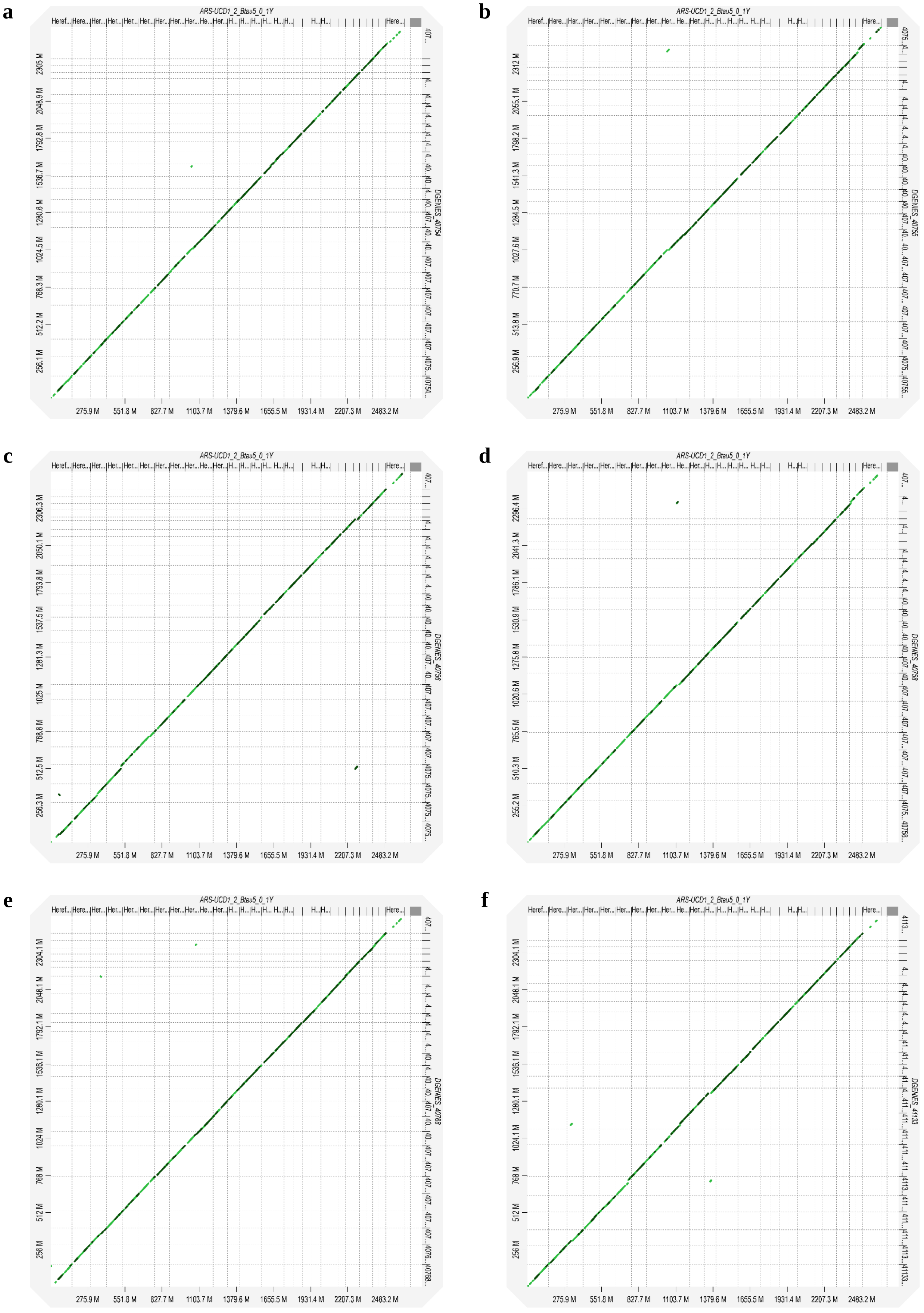
a
b
c
d
e
f
ARS-UCD1_2_Btau5_0_1Y
DGENIES_40754
DGENIES_40755
DGENIES_40756
DGENIES_40758
DGENIES_40768
DGENIES_41133

g

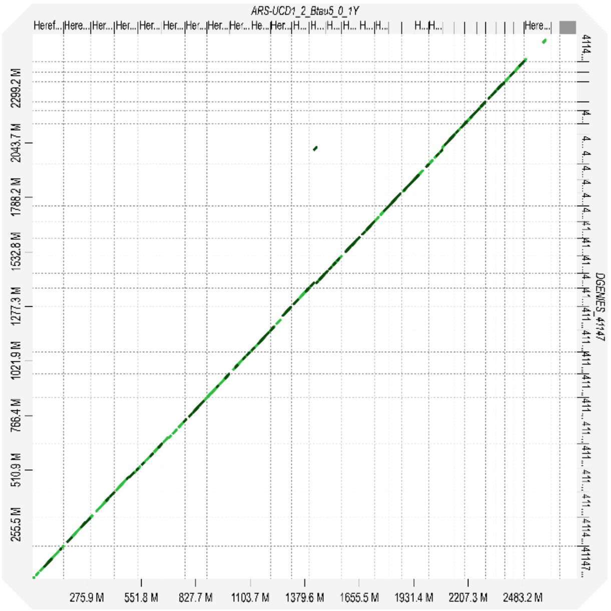

Figure S2 Chromosomal alignment concordance between ARS-UCD1.2 on *x*-axis and Aubrac assemblies on *y*-axis: **a)** 40754, **b)** 40755, **c)** 40756, **d)** 40758, **e)** 40768, **f)** 41133, and **g)** 41147

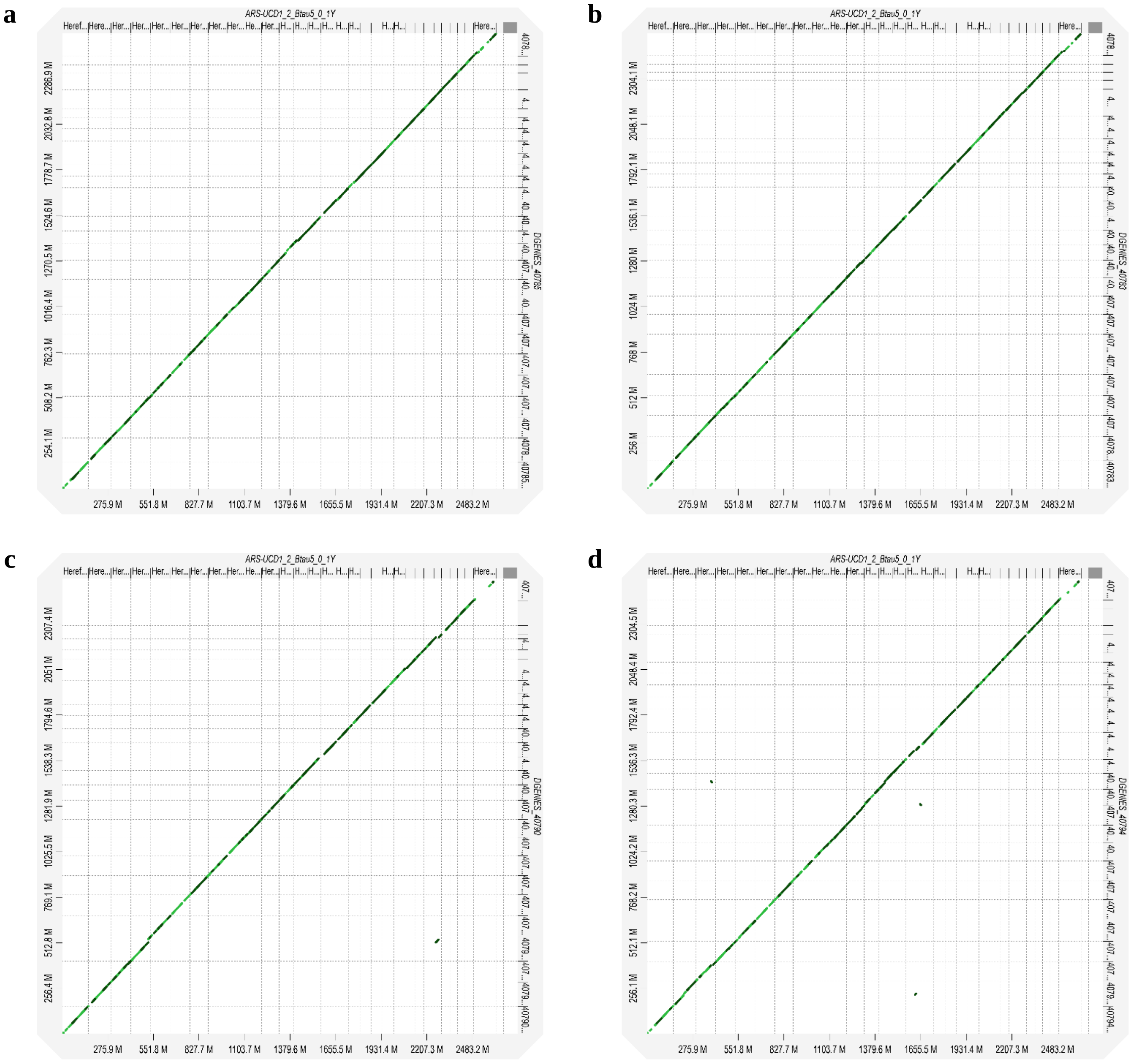

Figure S3 Chromosomal alignment concordance between ARS-UCD1.2 on *x*-axis and Blonde d'Aquitaine assemblies on *y*-axis: **a)** 40783, **b)** 40785, **c)** 40790, and **d)** 40794

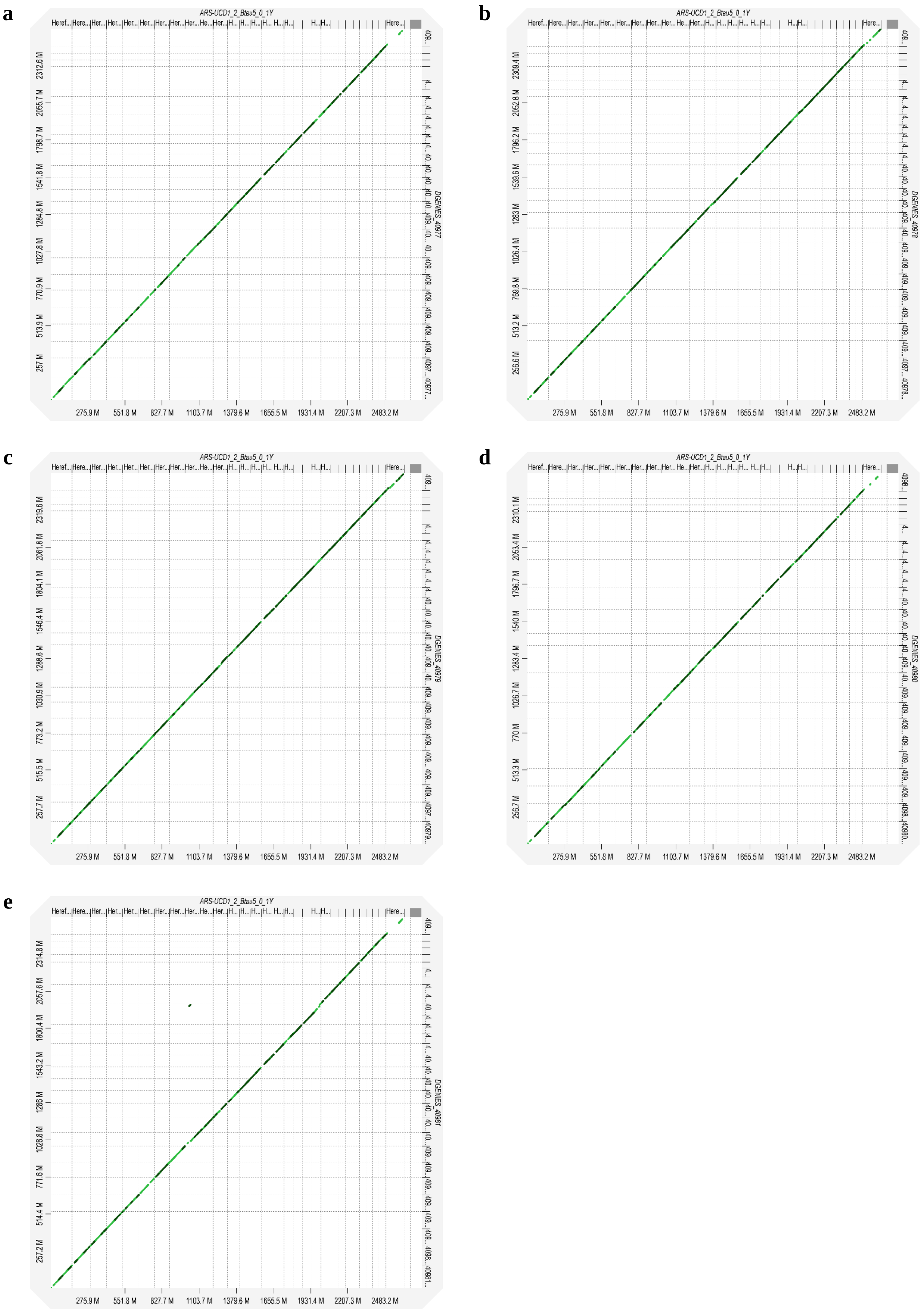

Figure S4 Chromosomal alignment concordance between ARS-UCD1.2 on *x*-axis and Brown Swiss assemblies on *y*-axis: **a)** 40977, **b)** 40978, **c)** 40979, **d)** 40980, and **e)** 40981

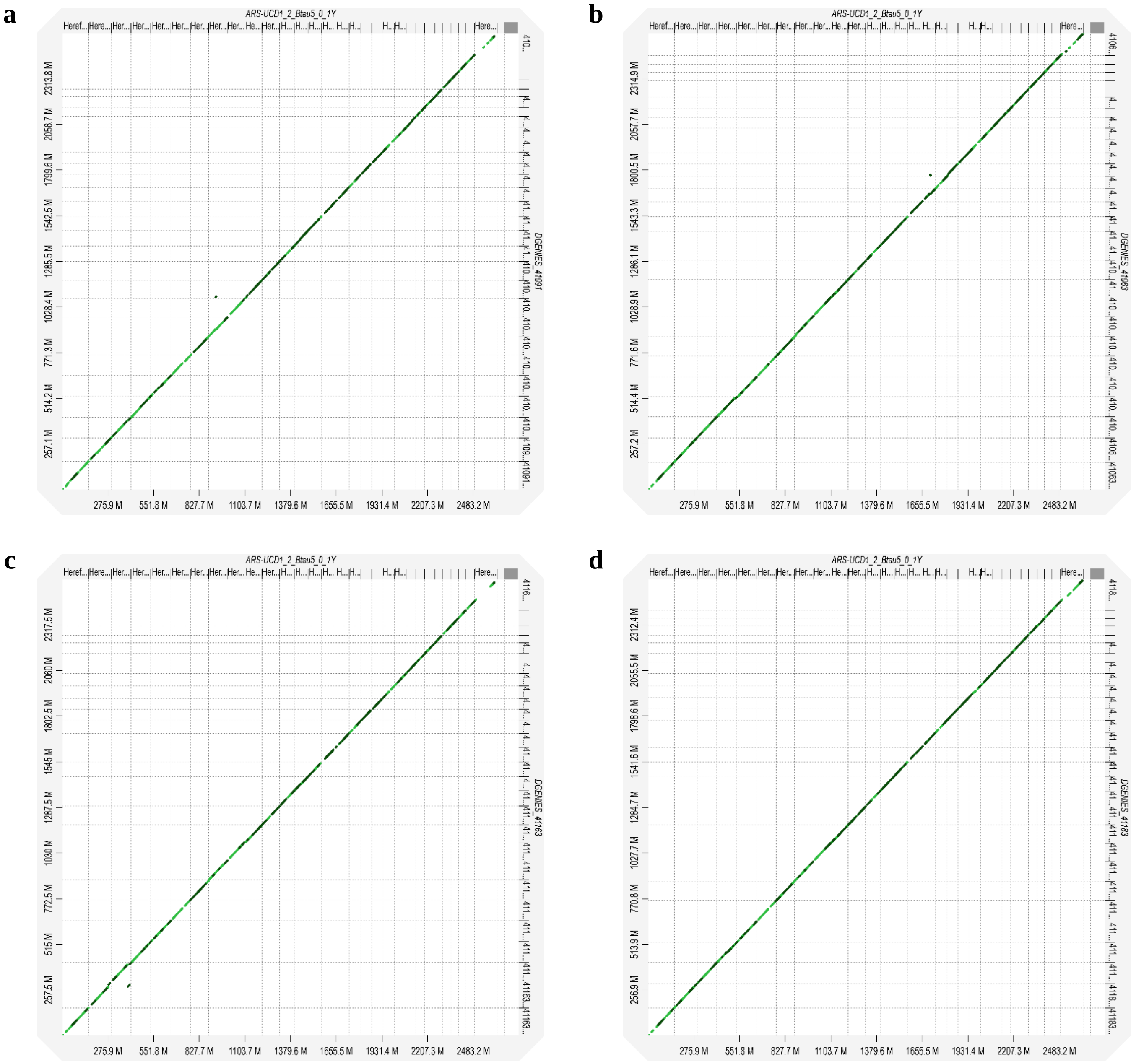

Figure S5 Chromosomal alignment concordance between ARS-UCD1.2 on *x*-axis and Charolaise assemblies on *y*-axis: **a)** 41063, **b)** 41091, **c)** 41163, and **d)** 41183

a

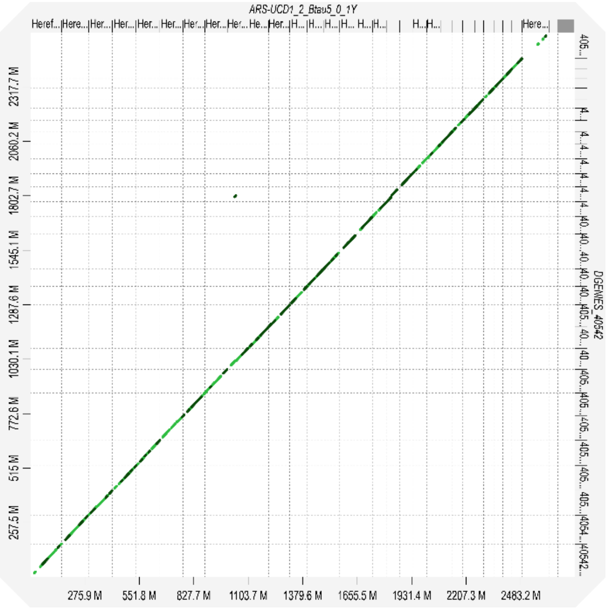
ARS-UCD1_2_Btau5_0_1Y
DGENES_40542
2317.7 M
2060.2 M
1802.7 M
1545.1 M
1287.6 M
1030.1 M
772.6 M
515 M
257.5 M
275.9 M
551.8 M
827.7 M
1103.7 M
1379.6 M
1655.5 M
1931.4 M
2207.3 M
2483.2 M

b

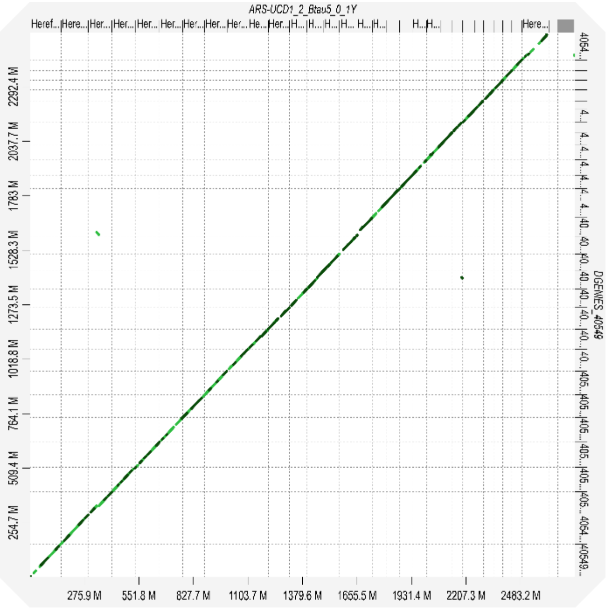
ARS-UCD1_2_Btau5_0_1Y
DGENES_40549
2292.4 M
2037.7 M
1783 M
1528.3 M
1273.5 M
1018.8 M
764.1 M
509.4 M
254.7 M
275.9 M
551.8 M
827.7 M
1103.7 M
1379.6 M
1655.5 M
1931.4 M
2207.3 M
2483.2 M

c

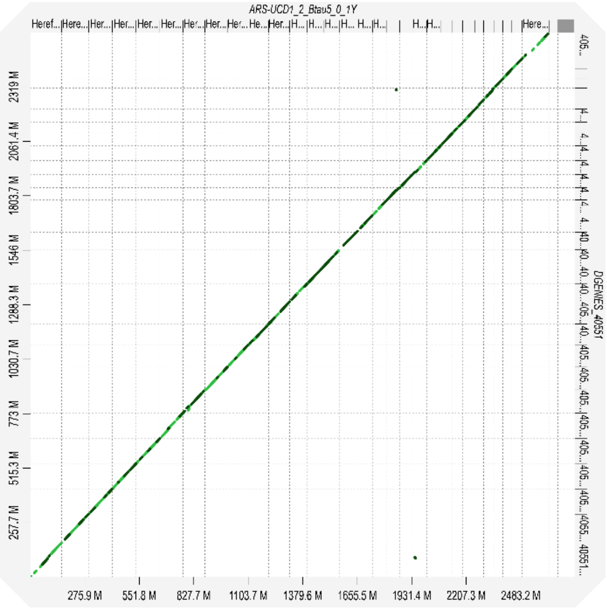
ARS-UCD1_2_Btau5_0_1Y
DGENES_40551
2319 M
2061.4 M
1803.7 M
1546 M
1288.3 M
1030.7 M
773 M
515.3 M
257.7 M
275.9 M
551.8 M
827.7 M
1103.7 M
1379.6 M
1655.5 M
1931.4 M
2207.3 M
2483.2 M

d

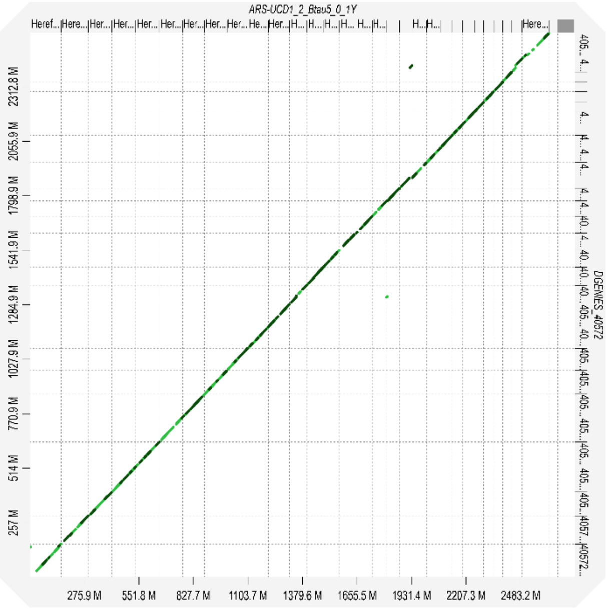
ARS-UCD1_2_Btau5_0_1Y
DGENES_40572
2312.8 M
2055.9 M
1798.9 M
1541.9 M
1284.9 M
1027.9 M
770.9 M
514 M
257 M
275.9 M
551.8 M
827.7 M
1103.7 M
1379.6 M
1655.5 M
1931.4 M
2207.3 M
2483.2 M

e

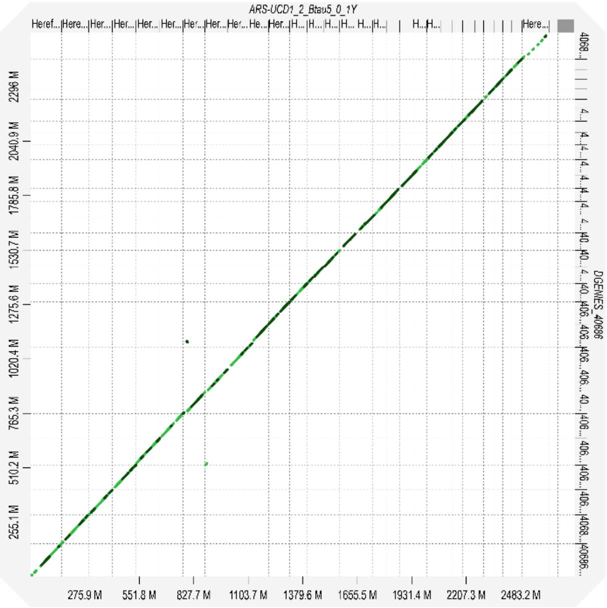
ARS-UCD1_2_Btau5_0_1Y
DGENES_40686
2296 M
2040.9 M
1785.8 M
1530.7 M
1275.6 M
1020.4 M
765.3 M
510.2 M
255.1 M
275.9 M
551.8 M
827.7 M
1103.7 M
1379.6 M
1655.5 M
1931.4 M
2207.3 M
2483.2 M

f

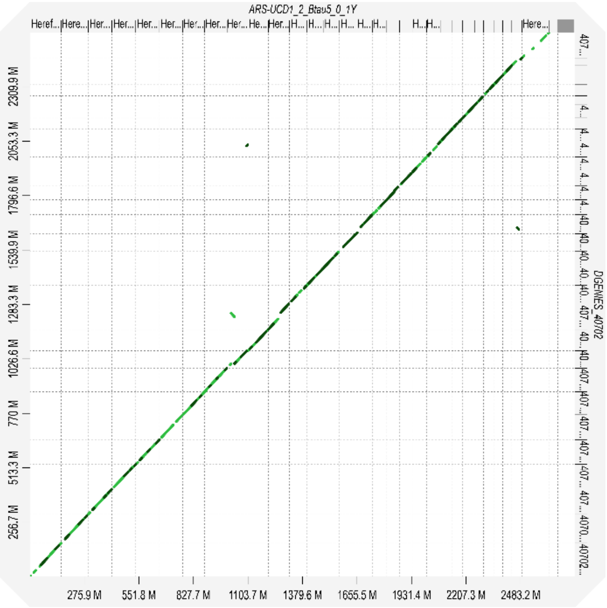
ARS-UCD1_2_Btau5_0_1Y
DGENES_40702
2309.9 M
2053.3 M
1796.6 M
1539.9 M
1283.3 M
1026.6 M
770 M
513.3 M
256.7 M
275.9 M
551.8 M
827.7 M
1103.7 M
1379.6 M
1655.5 M
1931.4 M
2207.3 M
2483.2 M

g

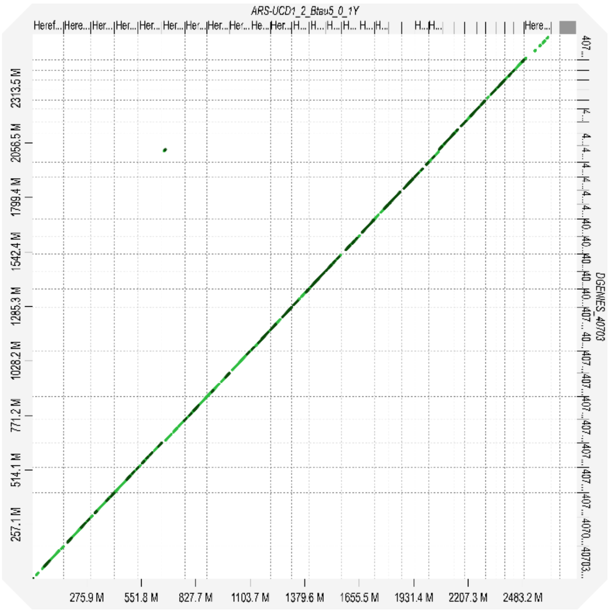

h

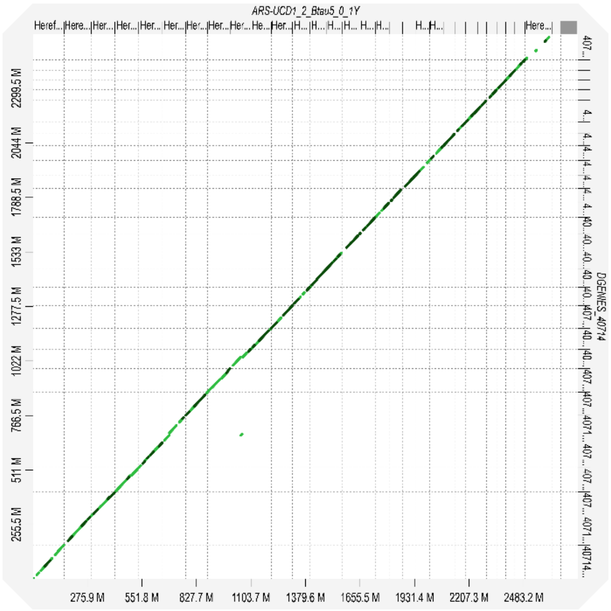

Figure S6 Chromosomal alignment concordance between ARS-UCD1.2 on *x*-axis and Holstein assemblies on *y*-axis: **a)** 40542, **b)** 40549, **c)** 40551, **d** 40572, **e)** 40686, **f)** 40702, **g)** 40703, and **h)** 40714

a

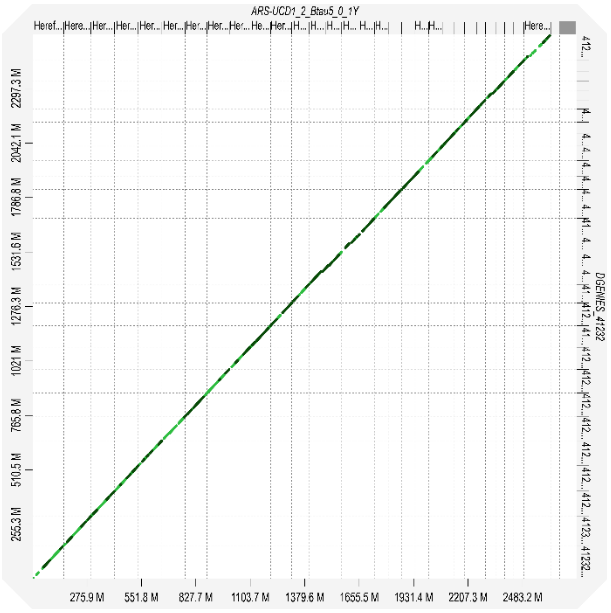

b

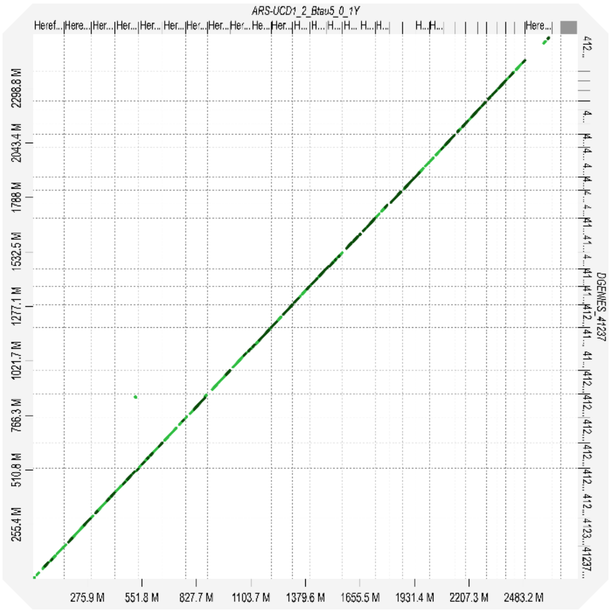

Figure S7 Chromosomal alignment concordance between ARS-UCD1.2 on *x*-axis and Limousine assemblies on *y*-axis: **a)** 41232, and **b)** 41237

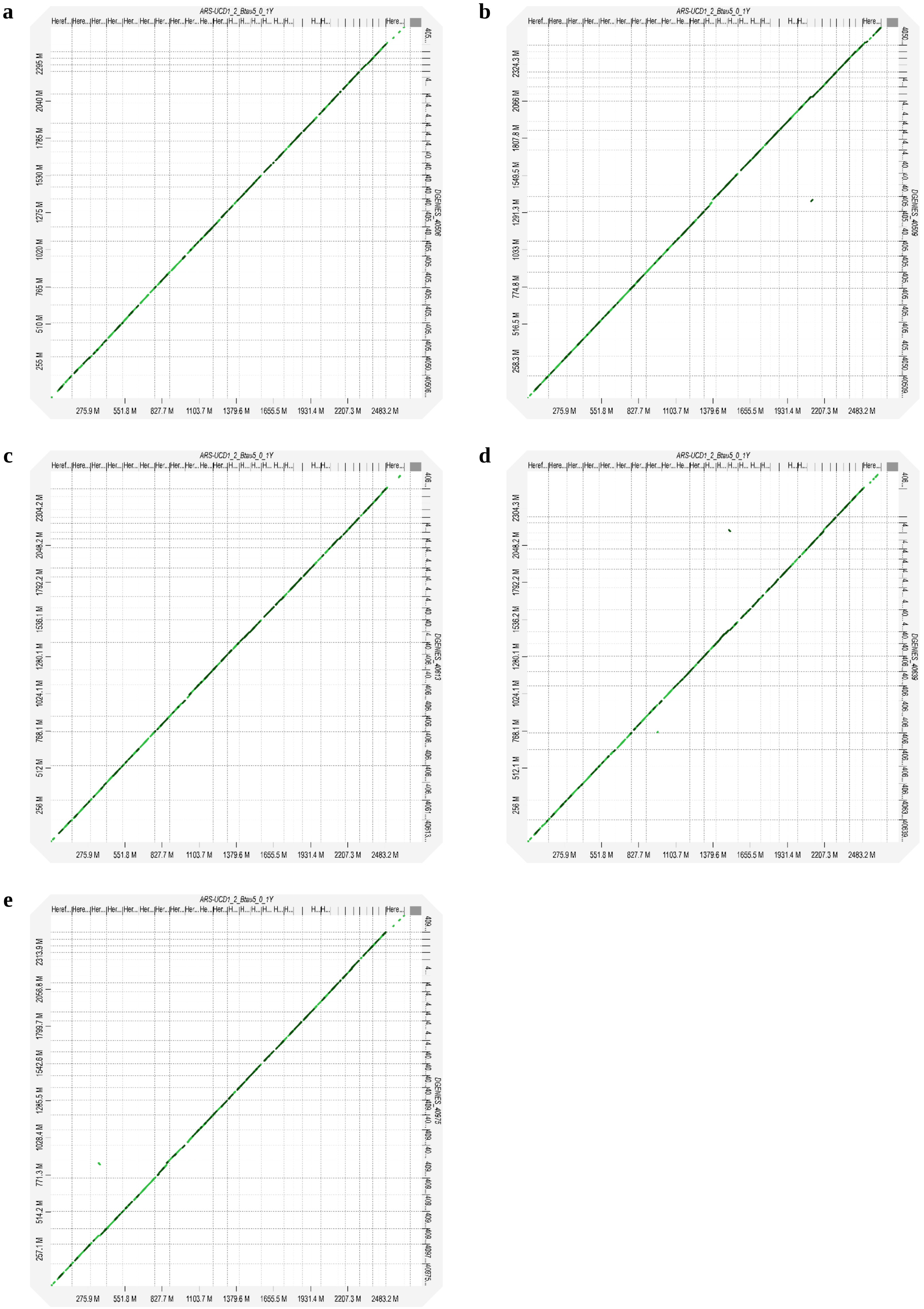

Figure S8 Chromosomal alignment concordance between ARS-UCD1.2 on *x*-axis and Montbéliarde assemblies on *y*-axis: **a)** 40506, **b)** 40509, **c)** 40613, **d)** 40639, and **e)** 40975

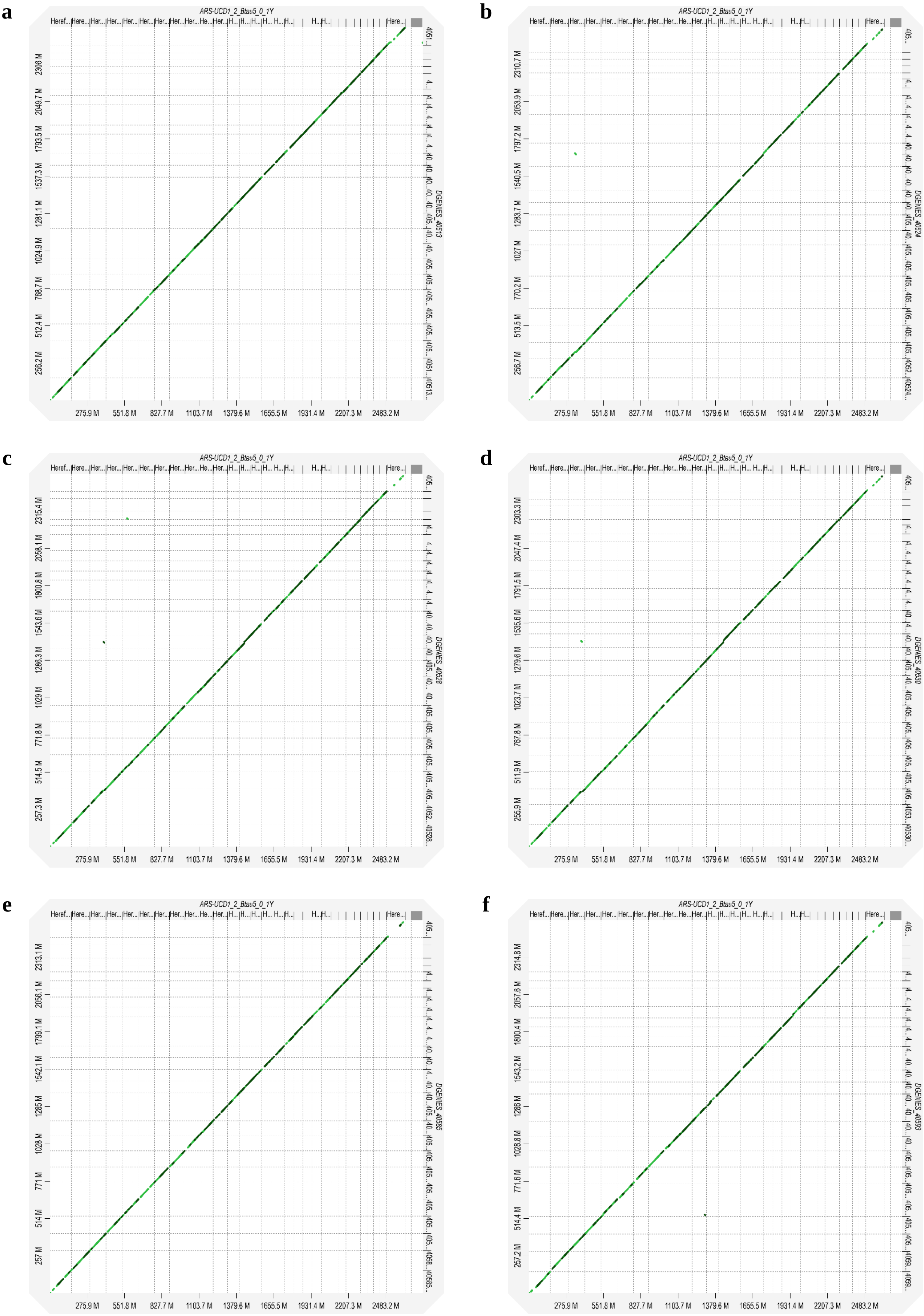
a
b
c
d
e
f
ARS-UCD1_2_Btau5_0_1Y
DGENIES_40513
DGENIES_40524
DGENIES_40528
DGENIES_40530
DGENIES_40586
DGENIES_40593
275.9 M
551.8 M
827.7 M
1103.7 M
1379.6 M
1655.5 M
1931.4 M
2207.3 M
2483.2 M

g

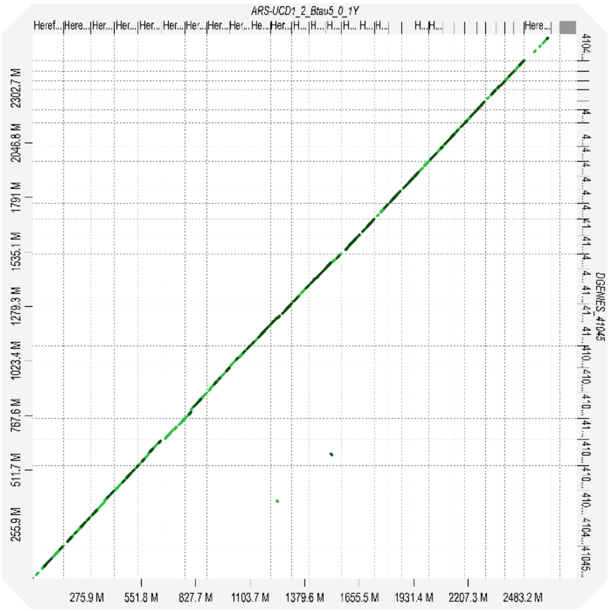

Figure S9 Chromosomal alignment concordance between ARS-UCD1.2 on *x*-axis and Normande assemblies on *y*-axis: **a)** 40513, **b)** 40524, **c)** 40528, **d)** 40530, **e)** 40585, **f)** 40593, and **g)** 41045

a

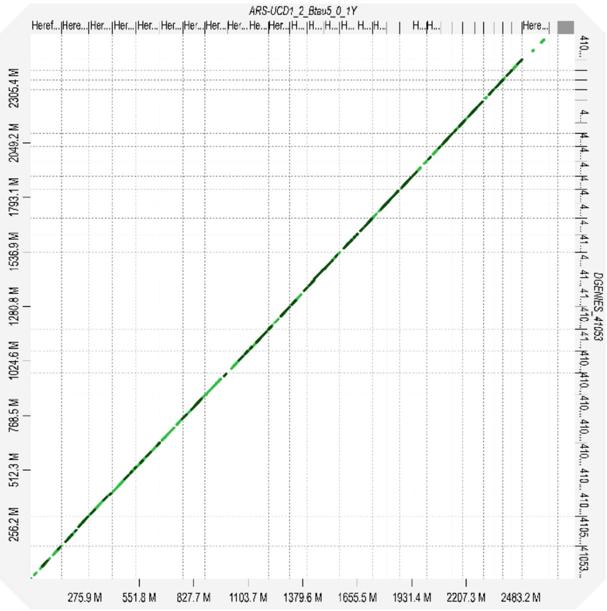

b

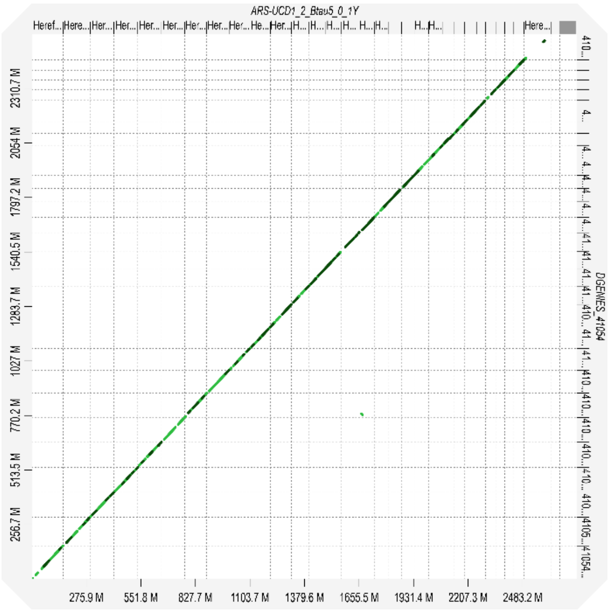

c

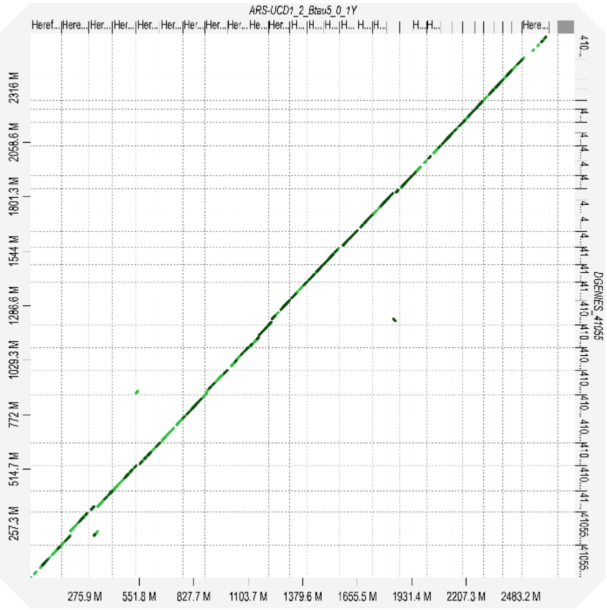

Figure S10 Chromosomal alignment concordance between ARS-UCD1.2 on *x*-axis and Parthenaise assemblies on *y*-axis: **a)** 41053, **b)** 41054, and **c)** 41055

a

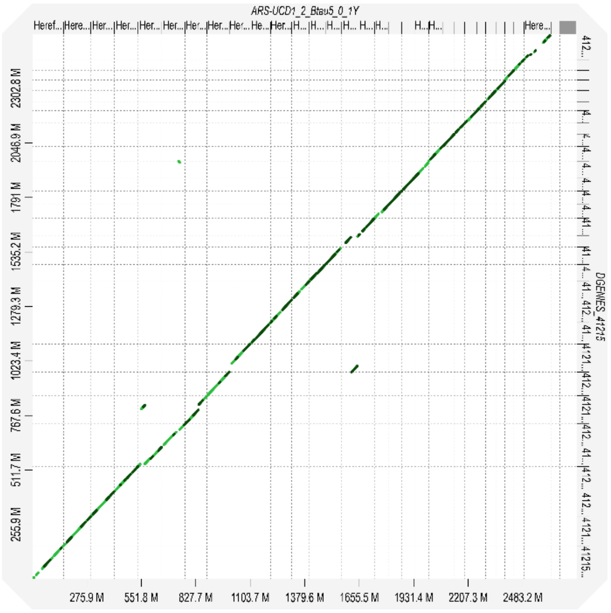

b

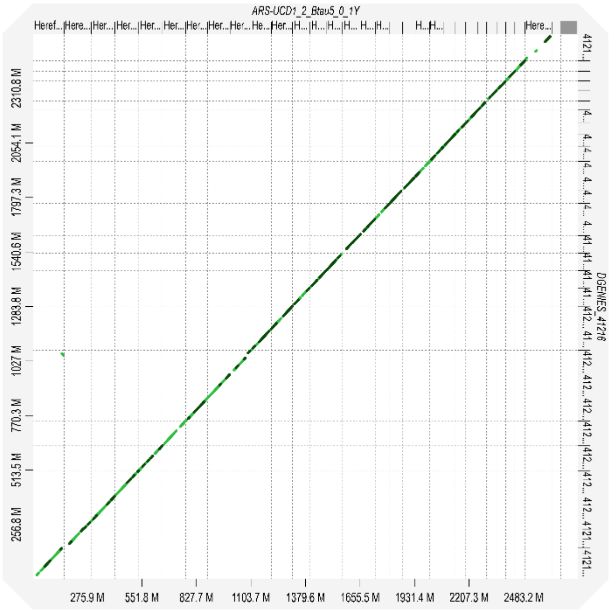

Figure S11 Chromosomal alignment concordance between ARS-UCD1.2 on *x*-axis and Rouge Flamande assemblies on *y*-axis: **a)** 41215, and **b)** 41216

a

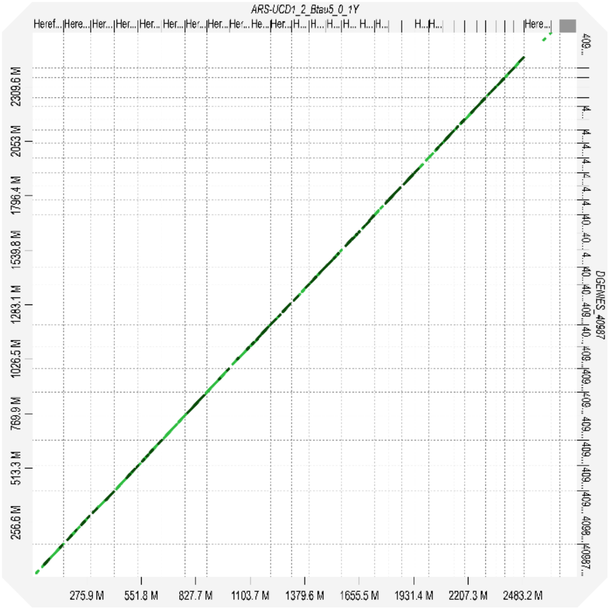

b

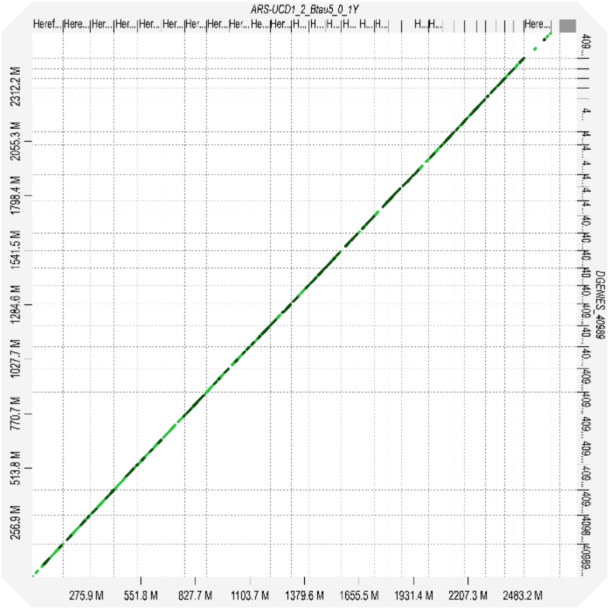

c

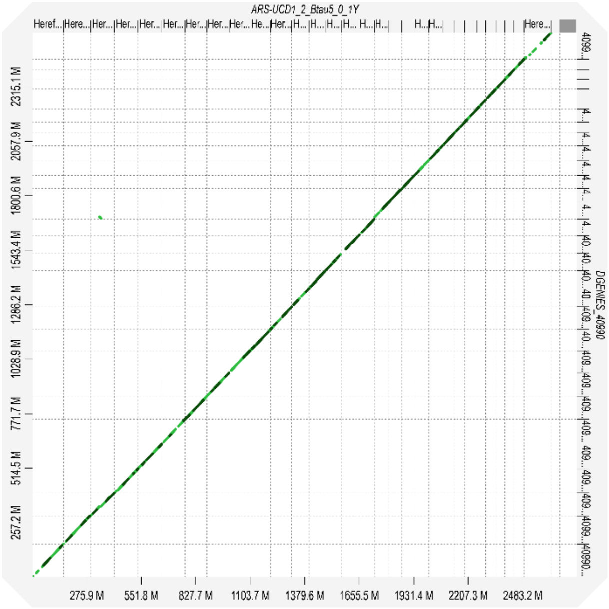

Figure S12 Chromosomal alignment concordance between ARS-UCD1.2 on *x*-axis and Simmental assemblies on *y*-axis: **a)** 40987, **b)** 40989, and **c)** 40990

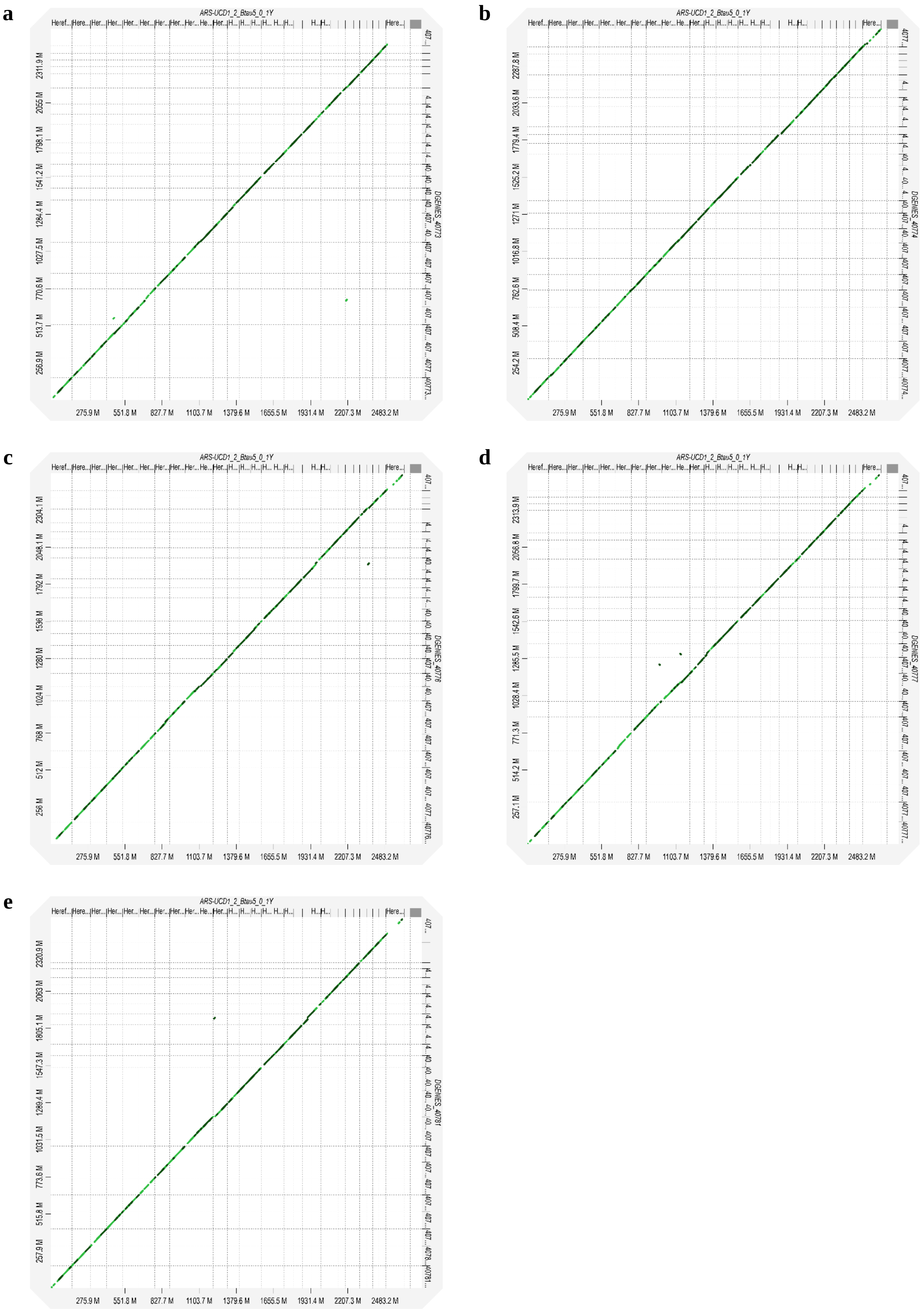

Figure S13 Chromosomal alignment concordance between ARS-UCD1.2 on *x*-axis and Tarentaise assemblies on *y*-axis: **a)** 40773, **b)** 40774, **c)** 40776, **d)** 40777, and **e)** 40781

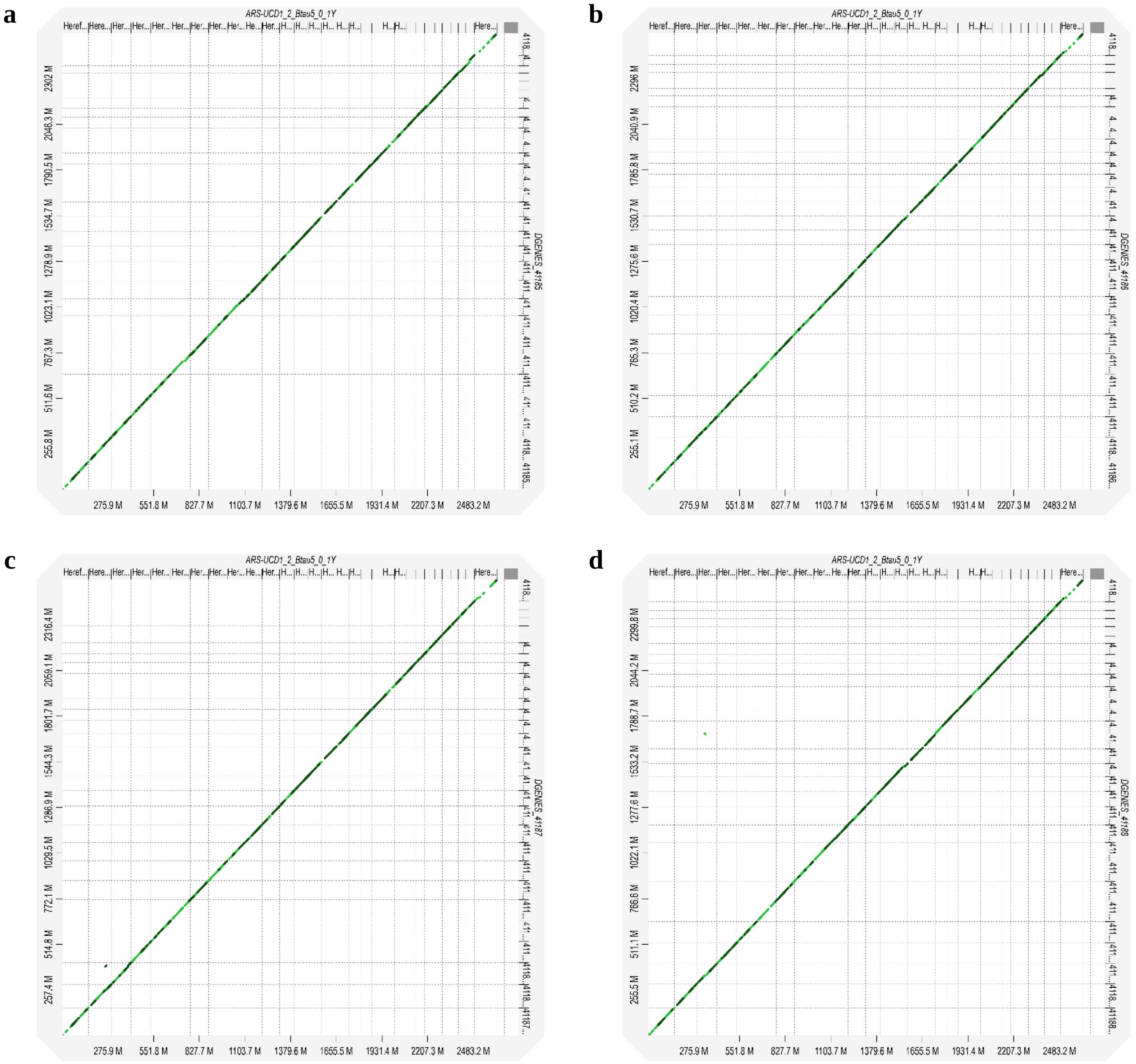

Figure S14 Chromosomal alignment concordance between ARS-UCD1.2 on *x*-axis and Vosgienne assemblies on *y*-axis: **a)** 41185, **b)** 41186, **c)** 41187, and **d)** 41188
