# Supplementary material for: Application of a French cattle pangenome, from structural variant discovery to association studies on key phenotypes
Source: Genet Sel Evol. 2025 Oct 23;57:61. doi: 10.1186/s12711-025-01012-x (PMC12551211; doi:10.1186/s12711-025-01012-x)
Supplement: Supplementary file 4 — Supplementary Material 4 Figures S15-S28 Size distribution of SVs classified as deletions and insertions, identified using Minigraph, and SyRI for the 14 breeds. Description: Plots were presented by breed in the following order: Abondance, Aubrac, Blonde d’Aquitaine, Brown Swiss, Charolaise, Holstein, Limousine, Montbéliarde, Normande, Parthenaise, Rouge Flamande, Simmental, Tarentaise, and Vosgienne [file 12711_2025_1012_MOESM4_ESM.pdf]

a

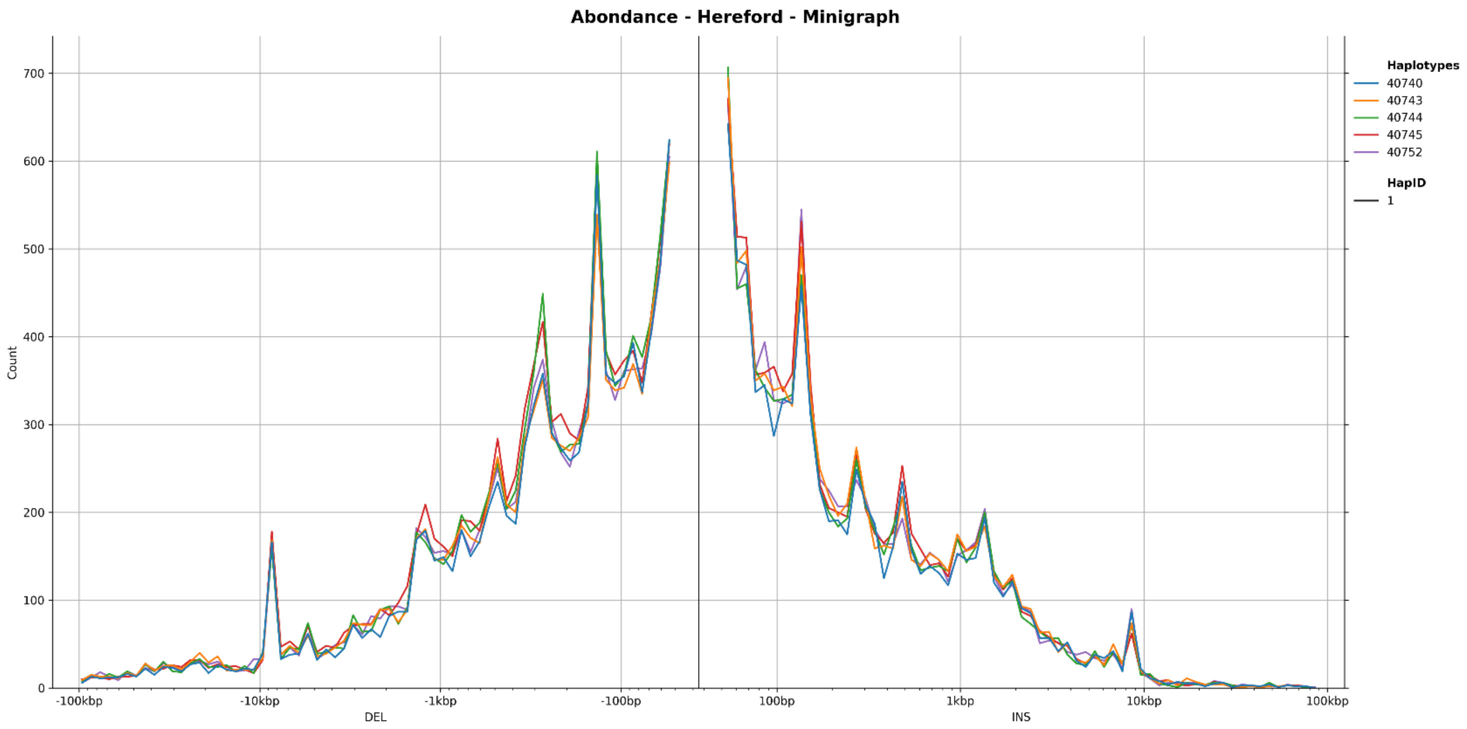

b

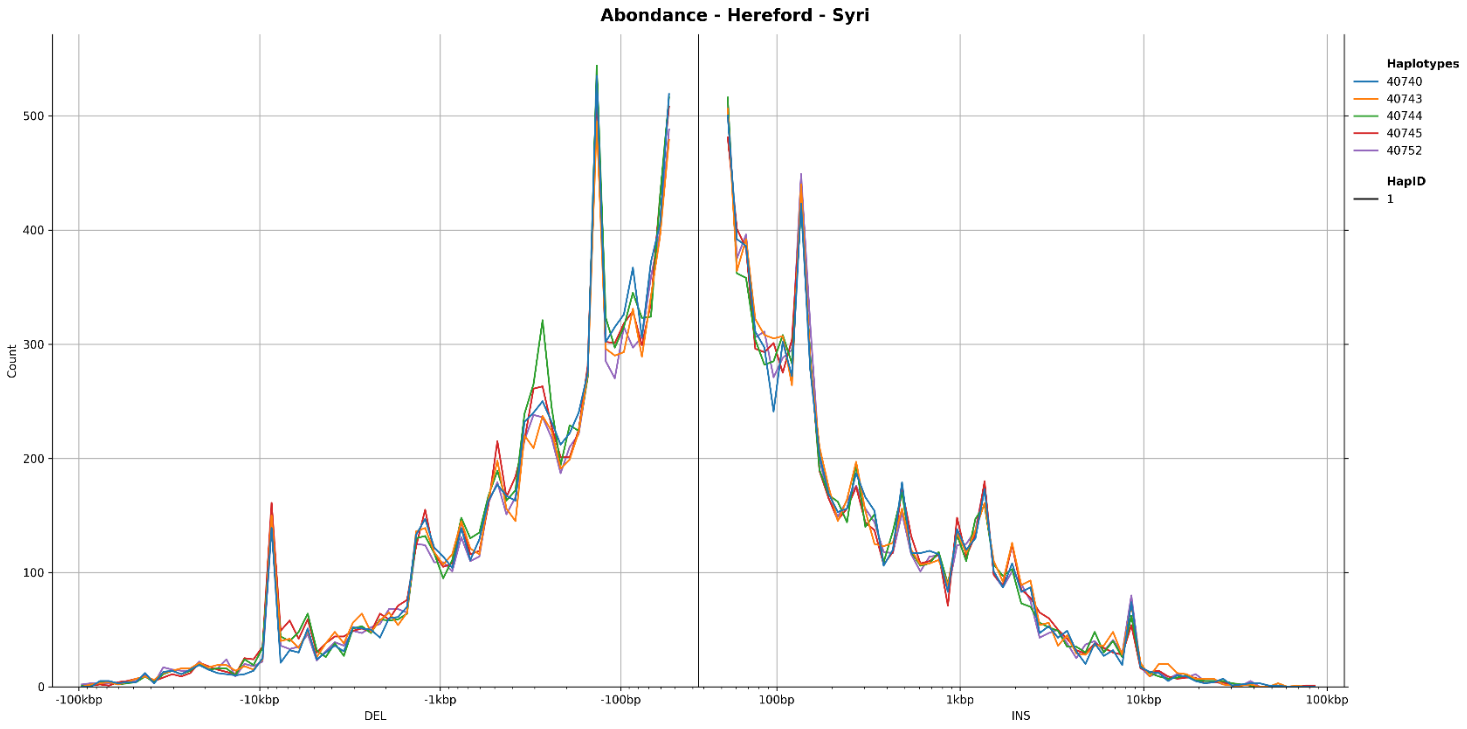

Figure S15 Size distribution of SVs classified as deletions (left) and insertions (right), identified using **a)** Minigraph, and **b)** SyRI for the Abondance breed

a

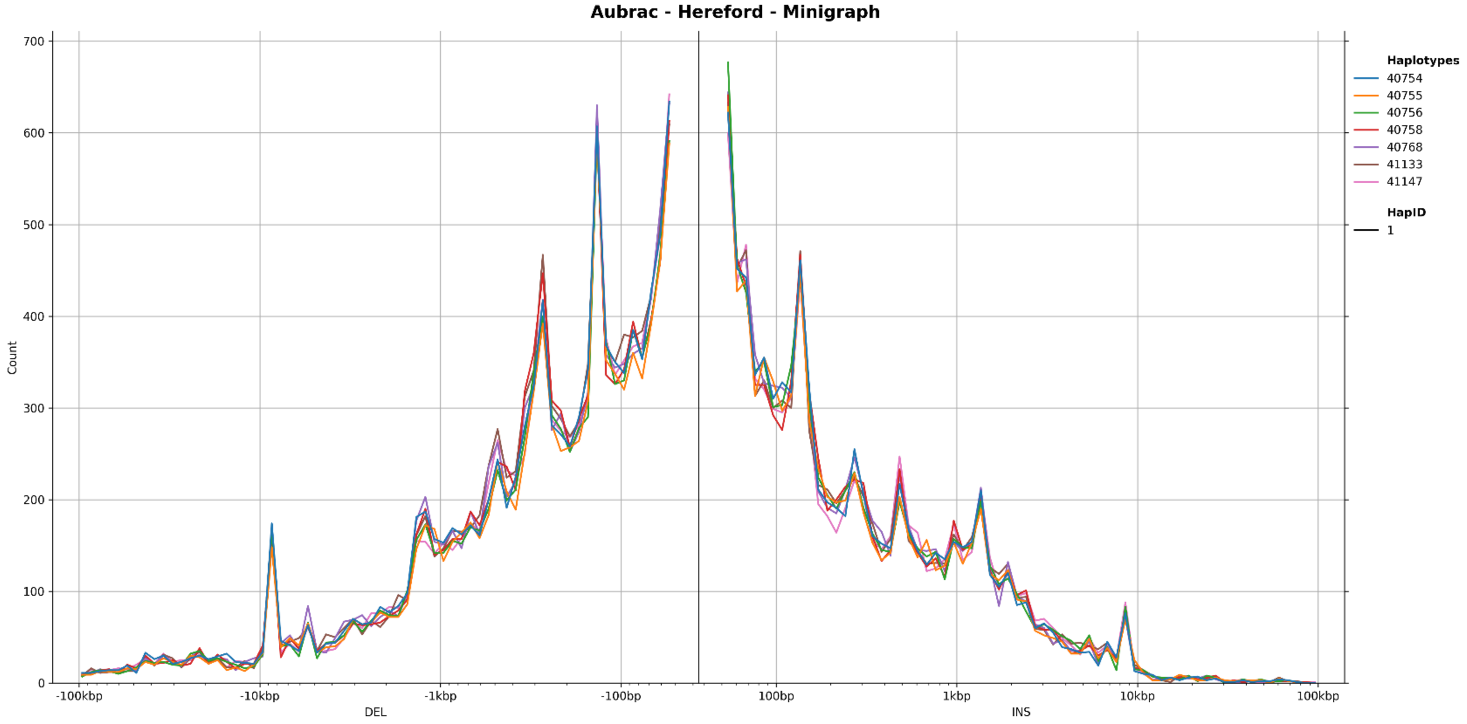

b

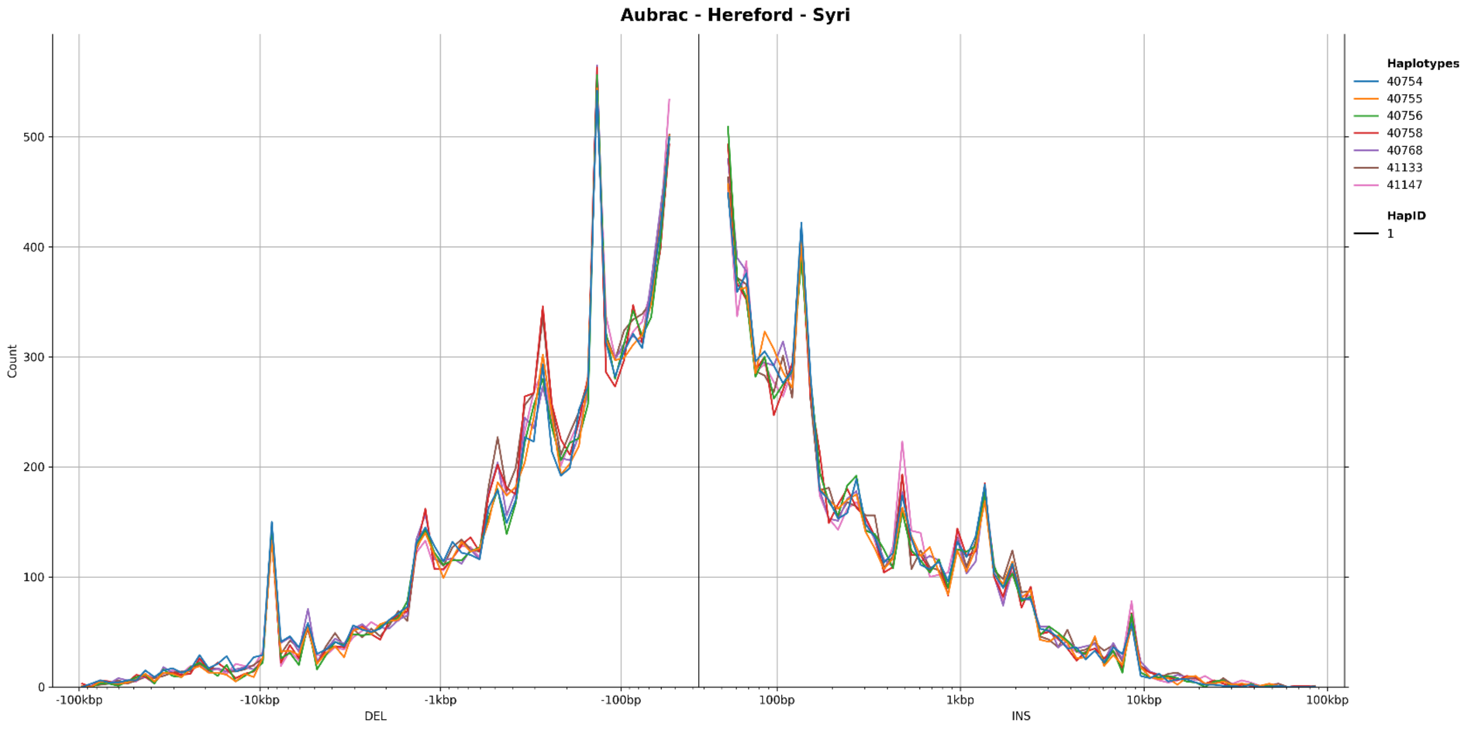

Figure S16 Size distribution of SVs classified as deletions (left) and insertions (right), identified using **a)** Minigraph, and **b)** SyRI for the Aubrac breed

a

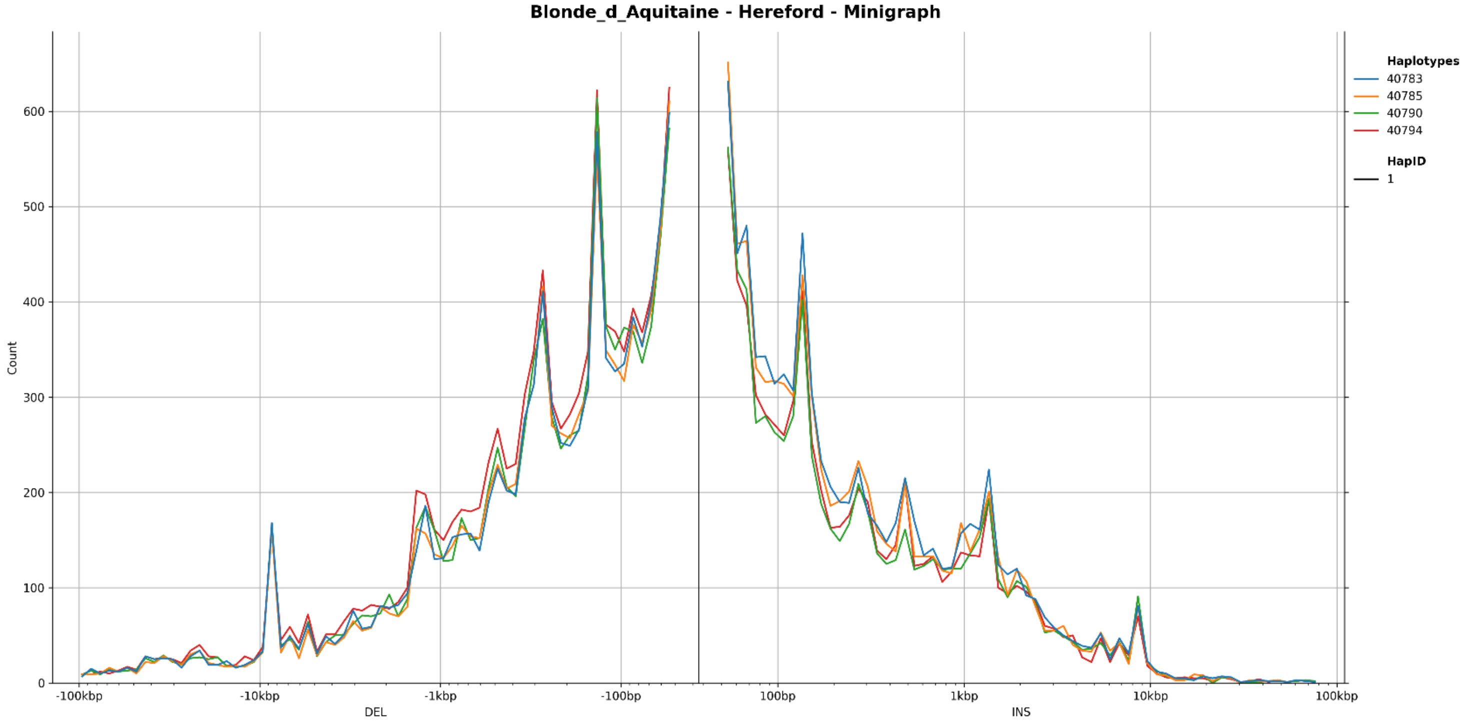

b

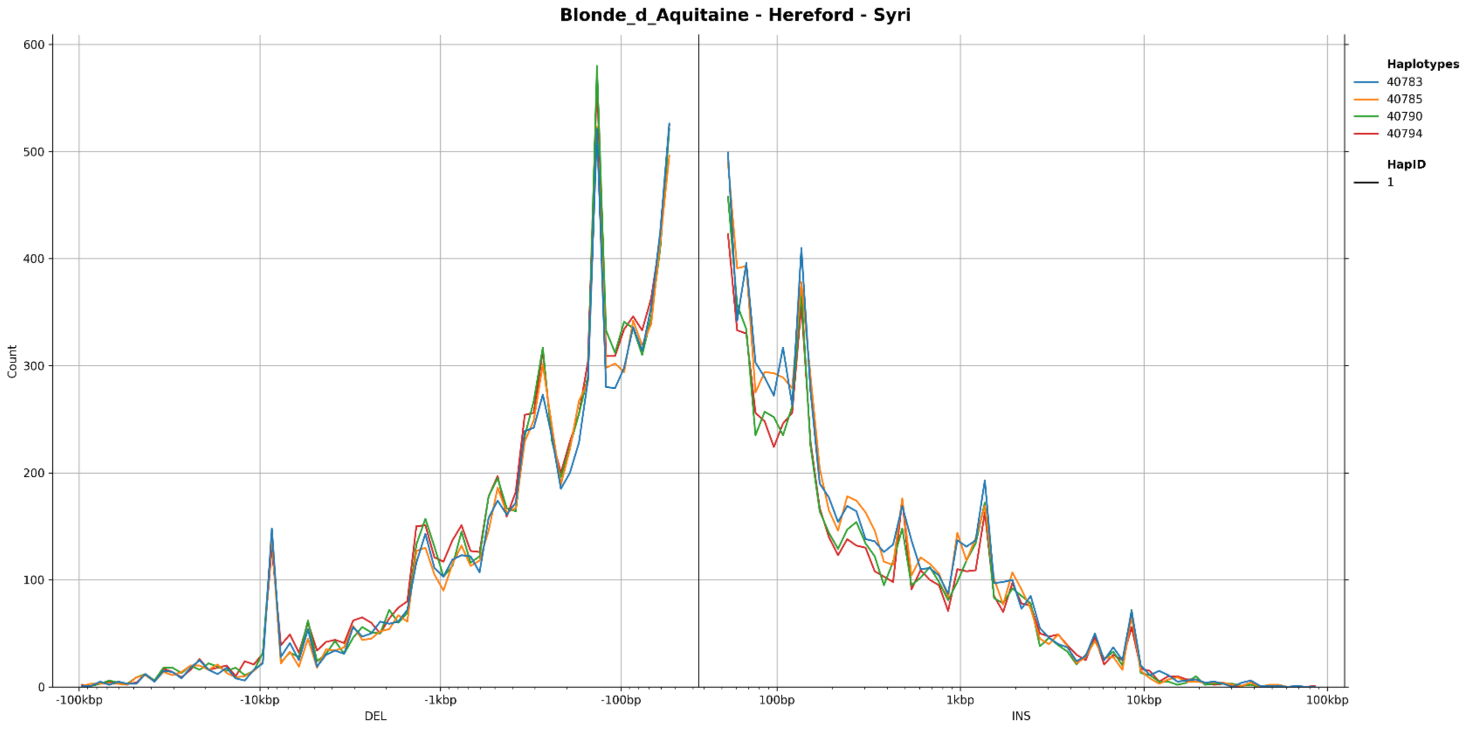

Figure S17 Size distribution of SVs classified as deletions (left) and insertions (right), identified using **a)** Minigraph, and **b)** SyRI for the Blonde d'Aquitaine breed

a

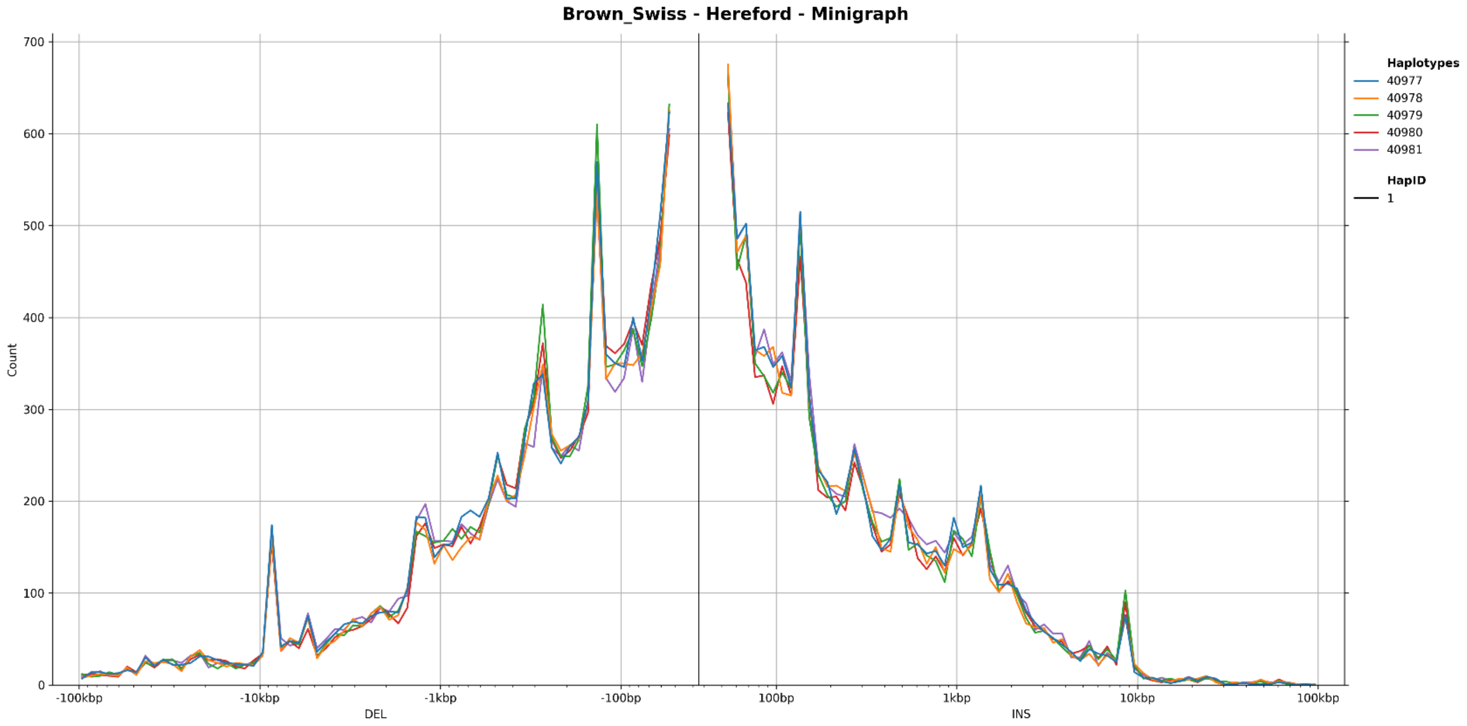

b

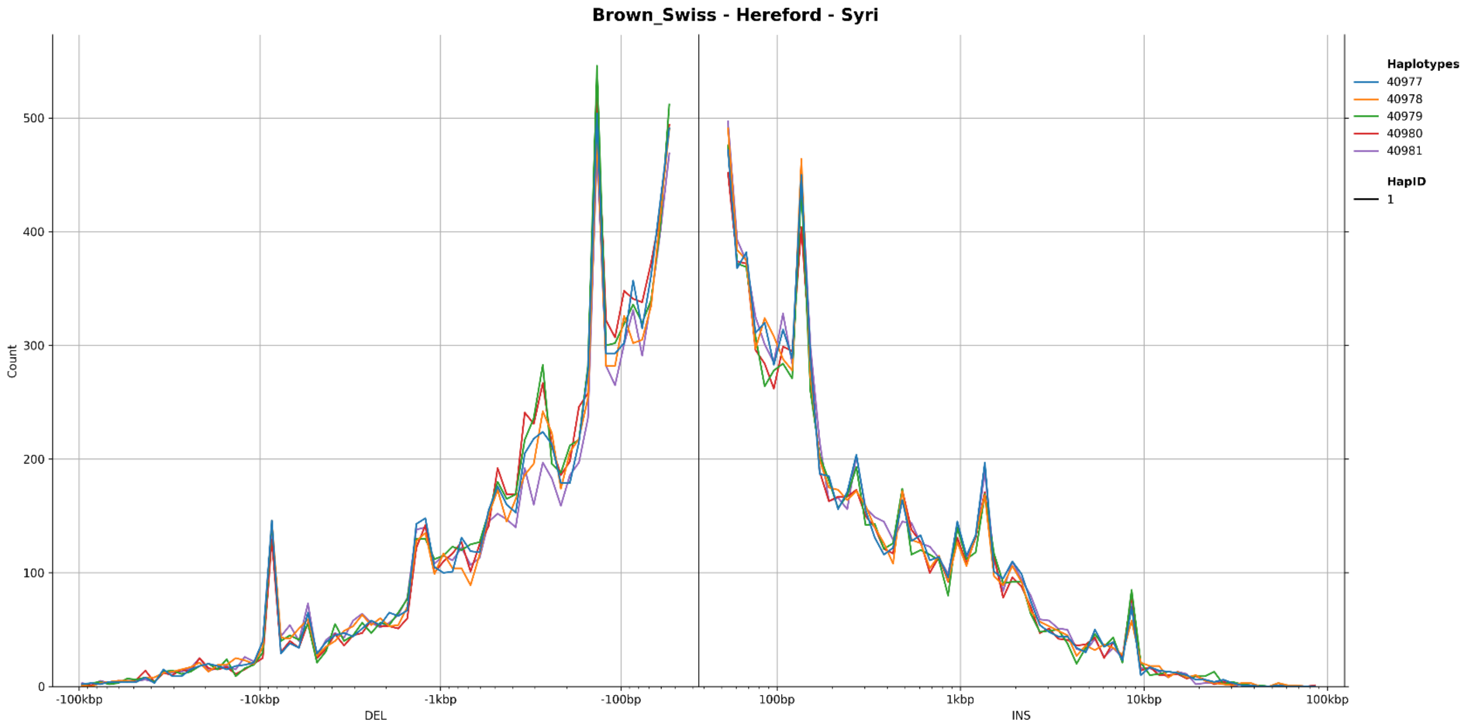

Figure S18 Size distribution of SVs classified as deletions (left) and insertions (right), identified using **a)** Minigraph, and **b)** SyRI for the Brown Swiss breed

a

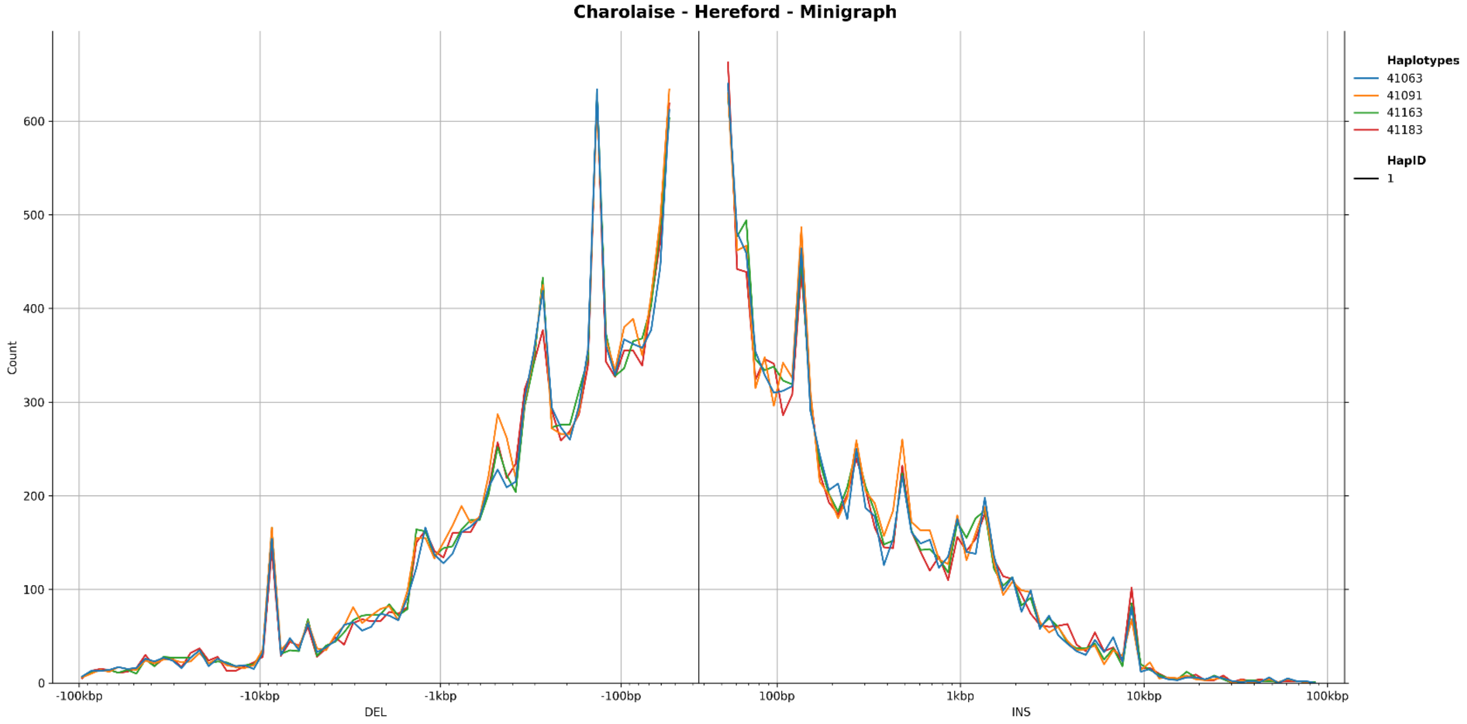

b

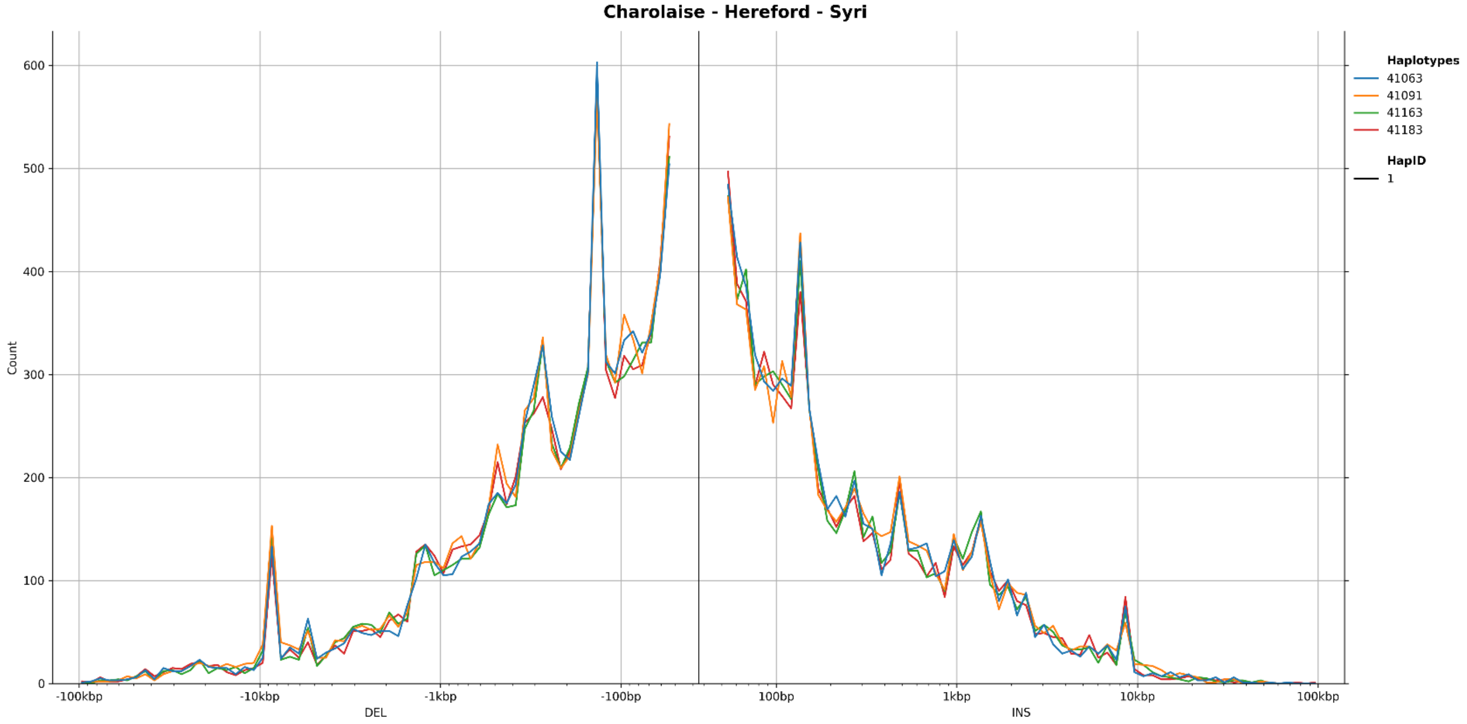

Figure S19 Size distribution of SVs classified as deletions (left) and insertions (right), identified using **a)** Minigraph, and **b)** SyRI for the Charolaise breed

a

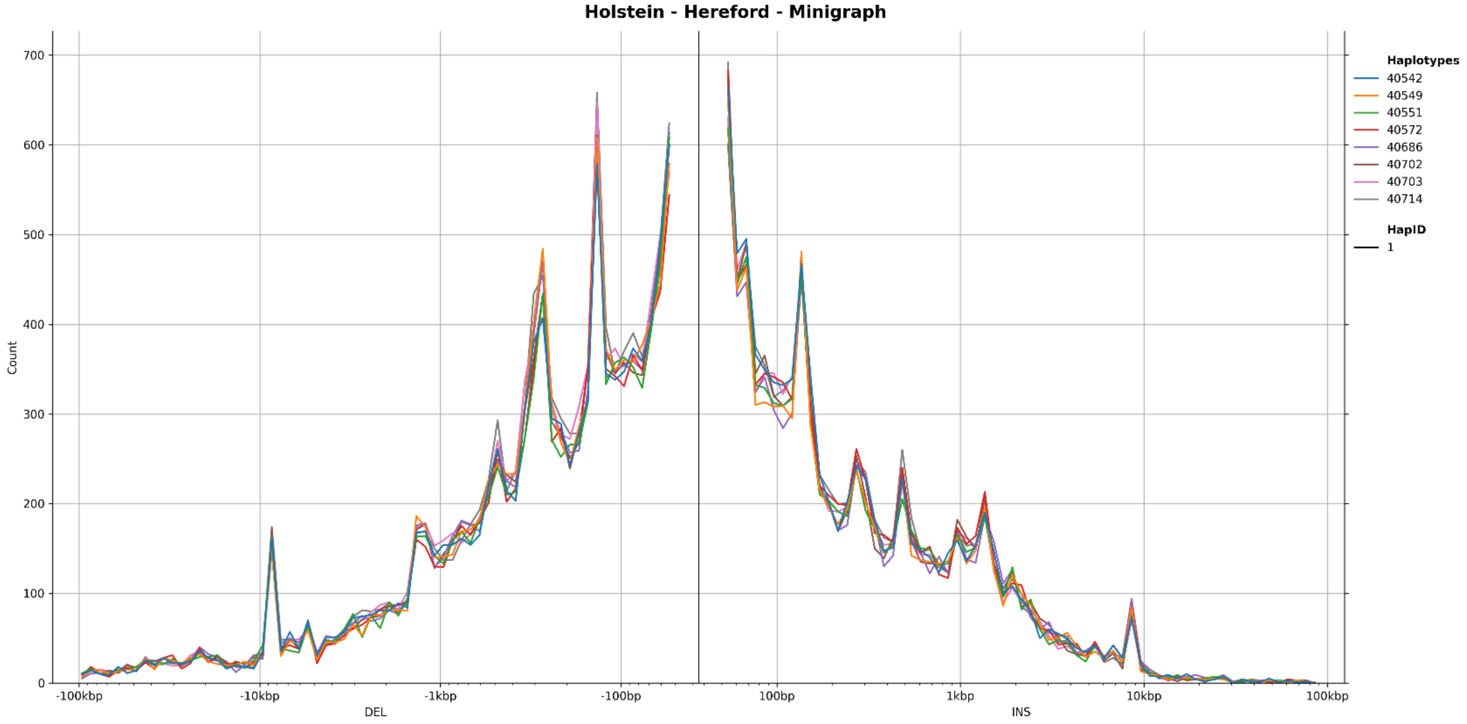

b

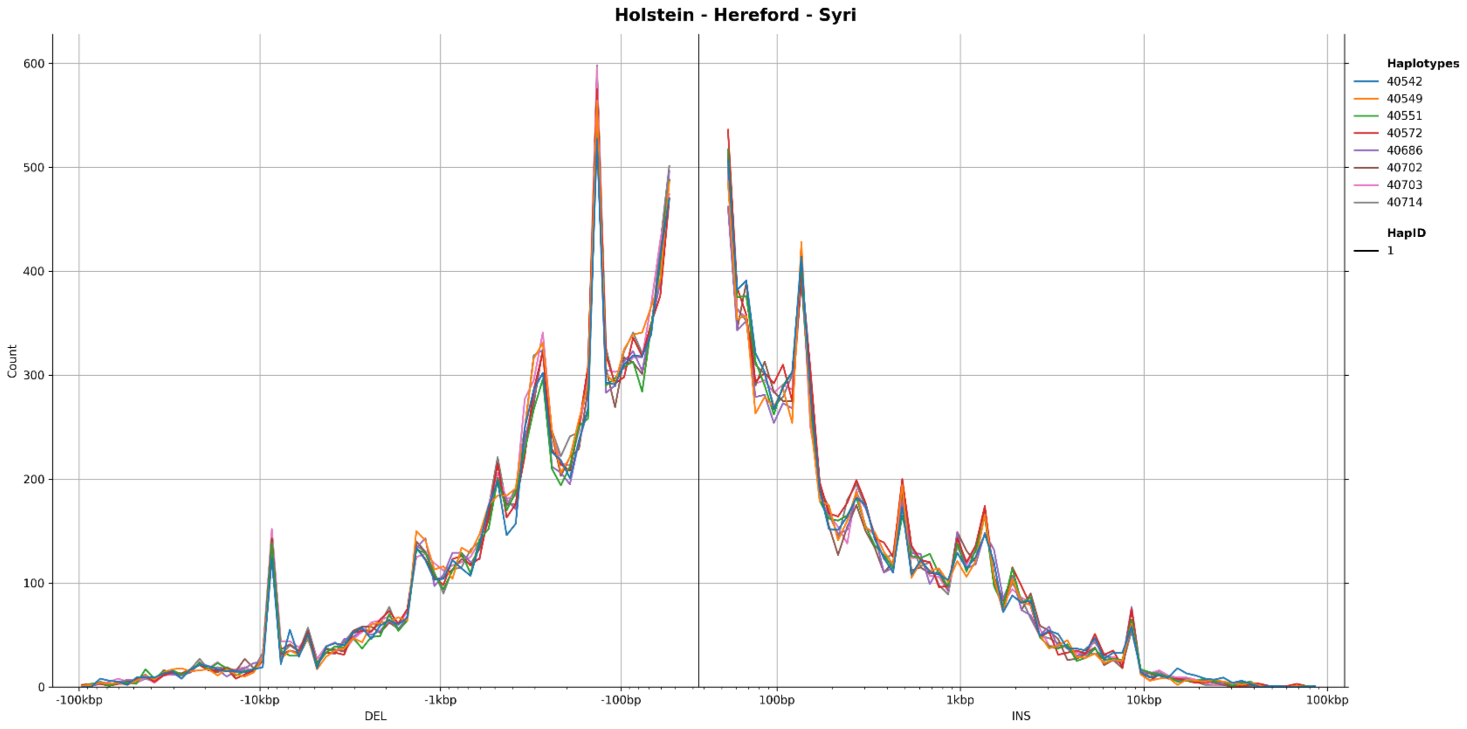

Figure S20 Size distribution of SVs classified as deletions (left) and insertions (right), identified using **a)** Minigraph, and **b)** SyRI for the Holstein breed

a

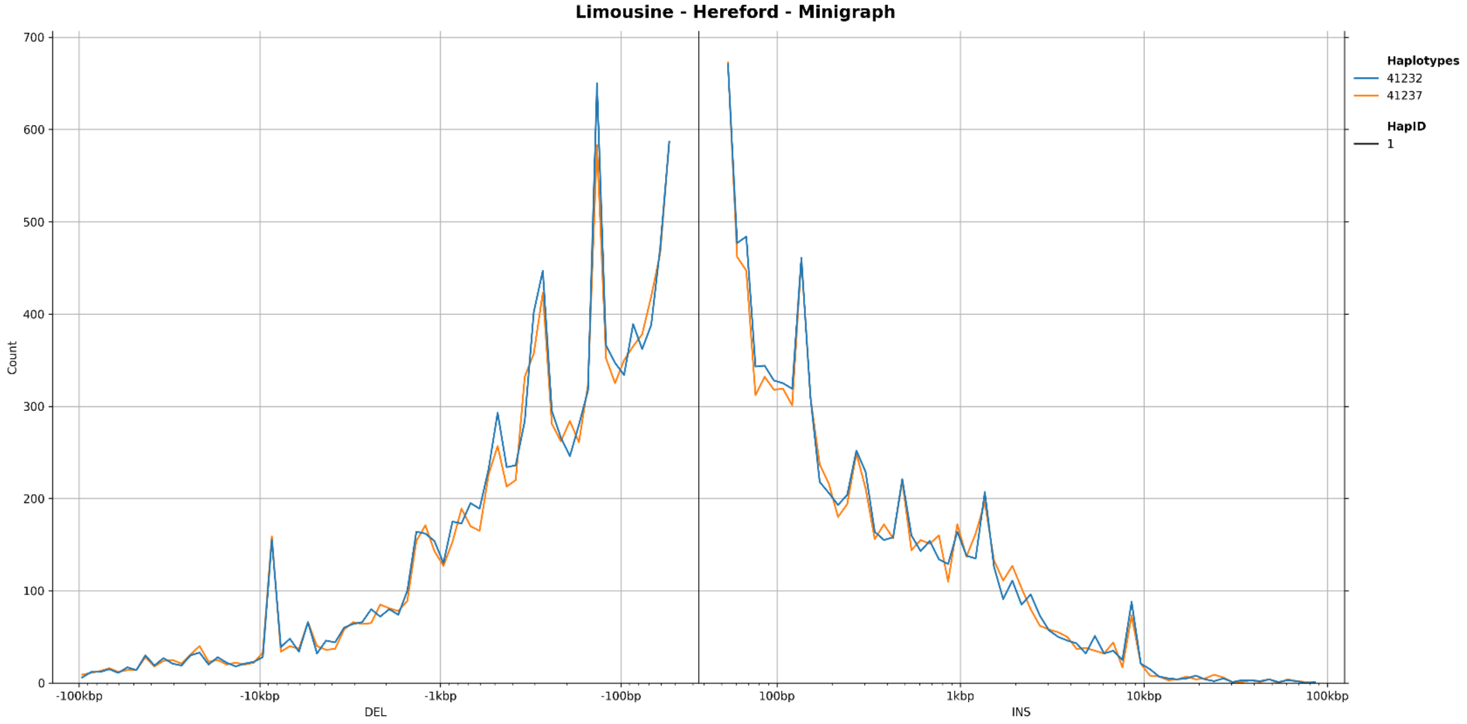

b

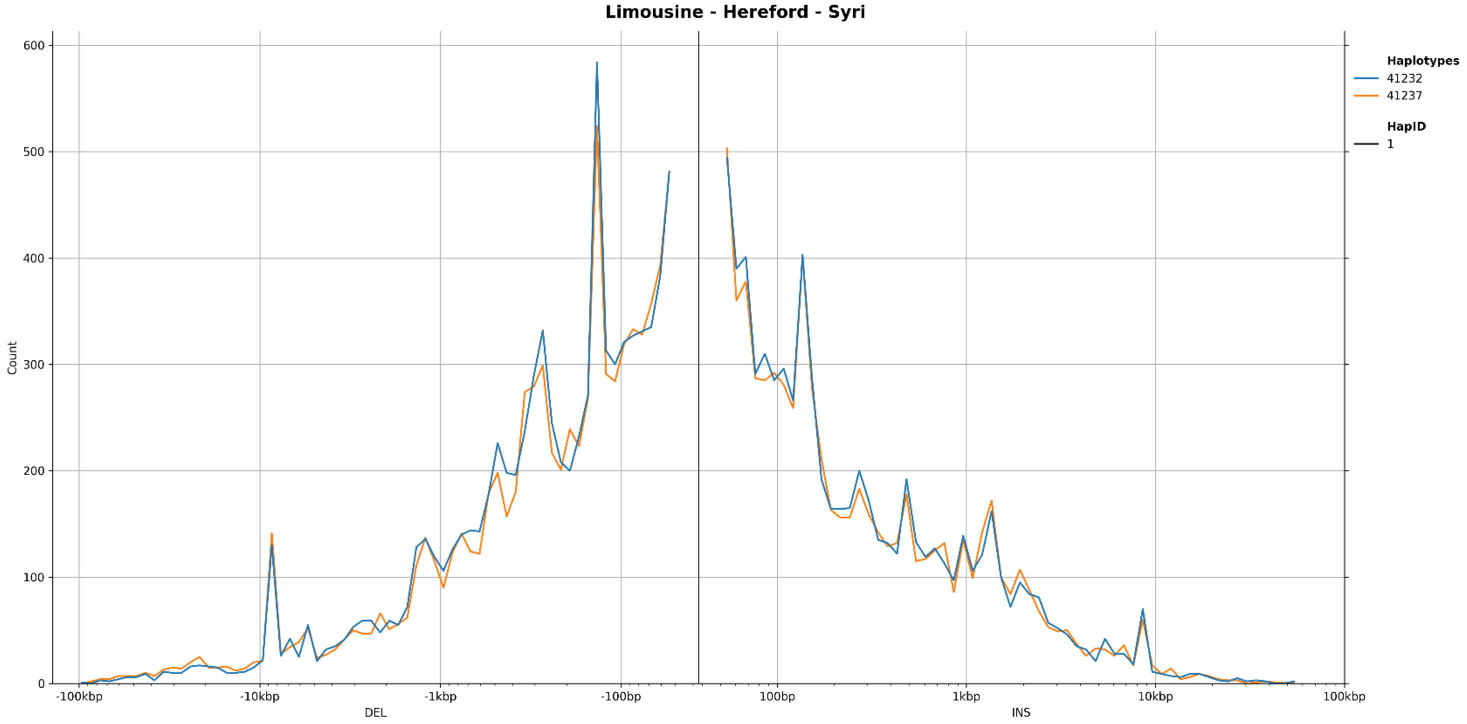

Figure S21 Size distribution of SVs classified as deletions (left) and insertions (right), identified using **a)** Minigraph, and **b)** SyRI for the Limousine breed

a

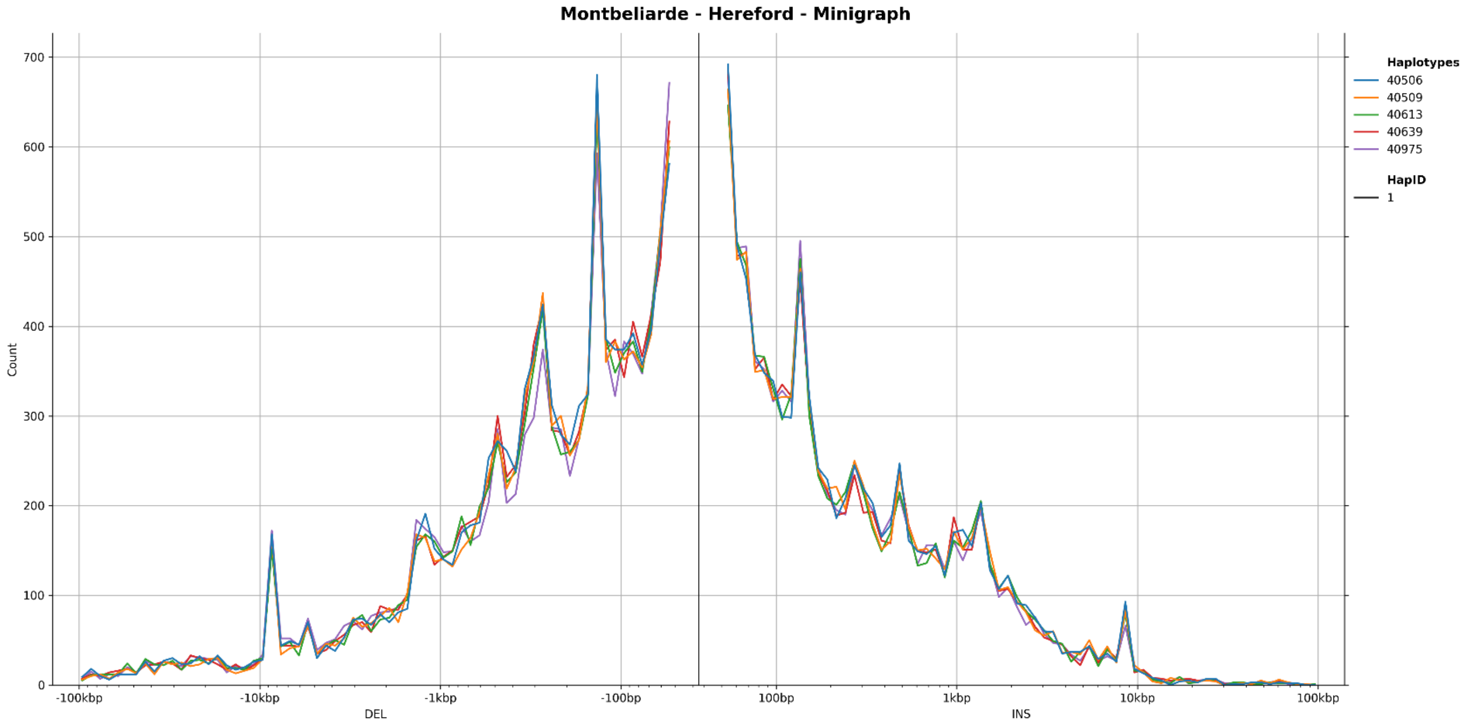

b

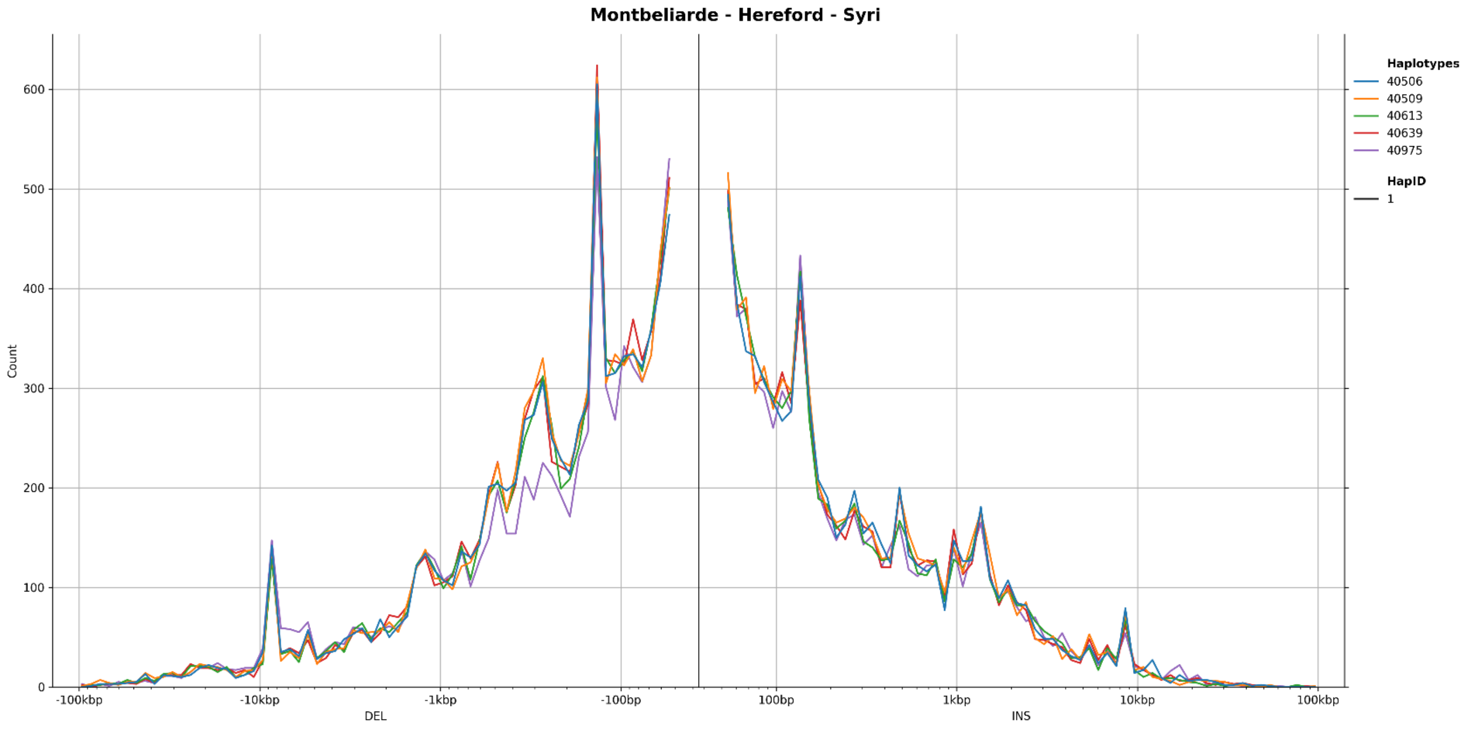

Figure S22 Size distribution of SVs classified as deletions (left) and insertions (right), identified using **a)** Minigraph, and **b)** SyRI for the Montbéliarde breed

a

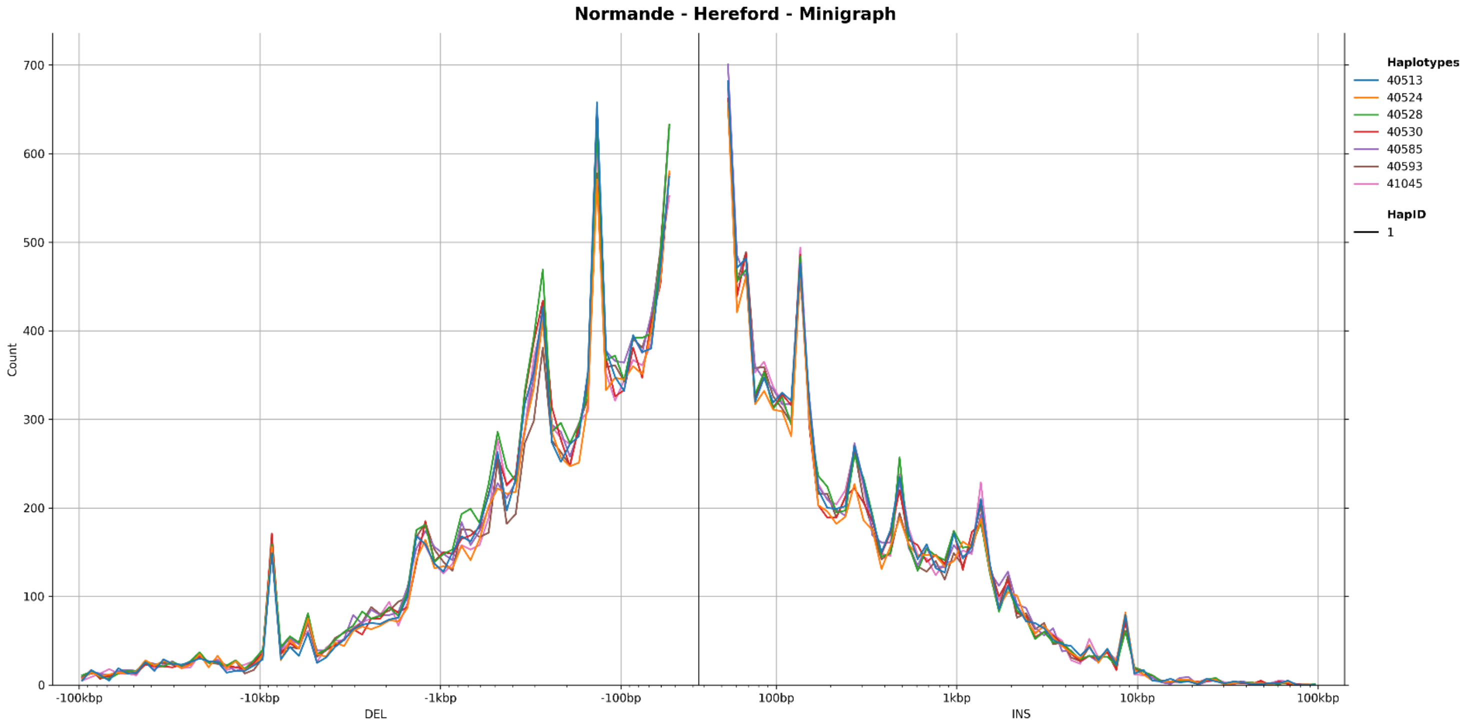

b

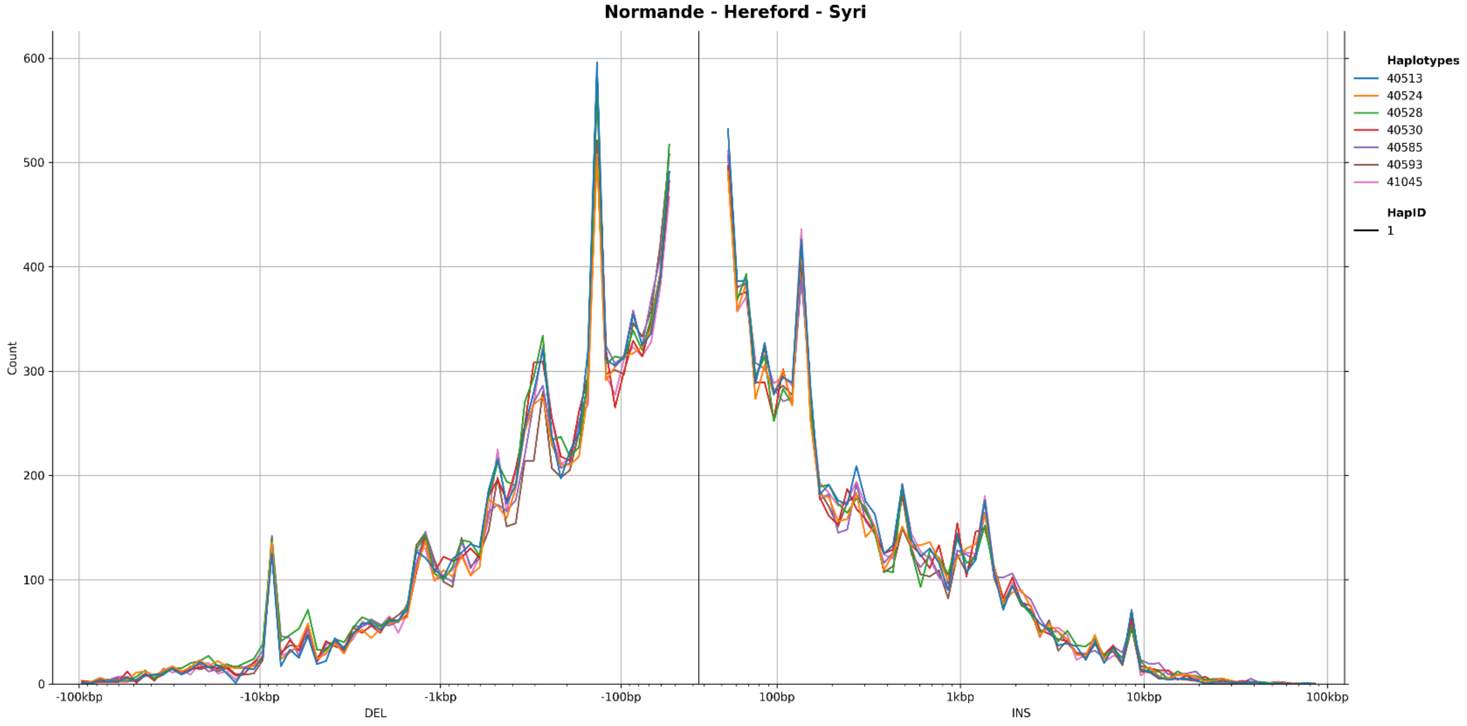

Figure S23 Size distribution of SVs classified as deletions (left) and insertions (right), identified using **a)** Minigraph, and **b)** SyRI for the Normande breed

a

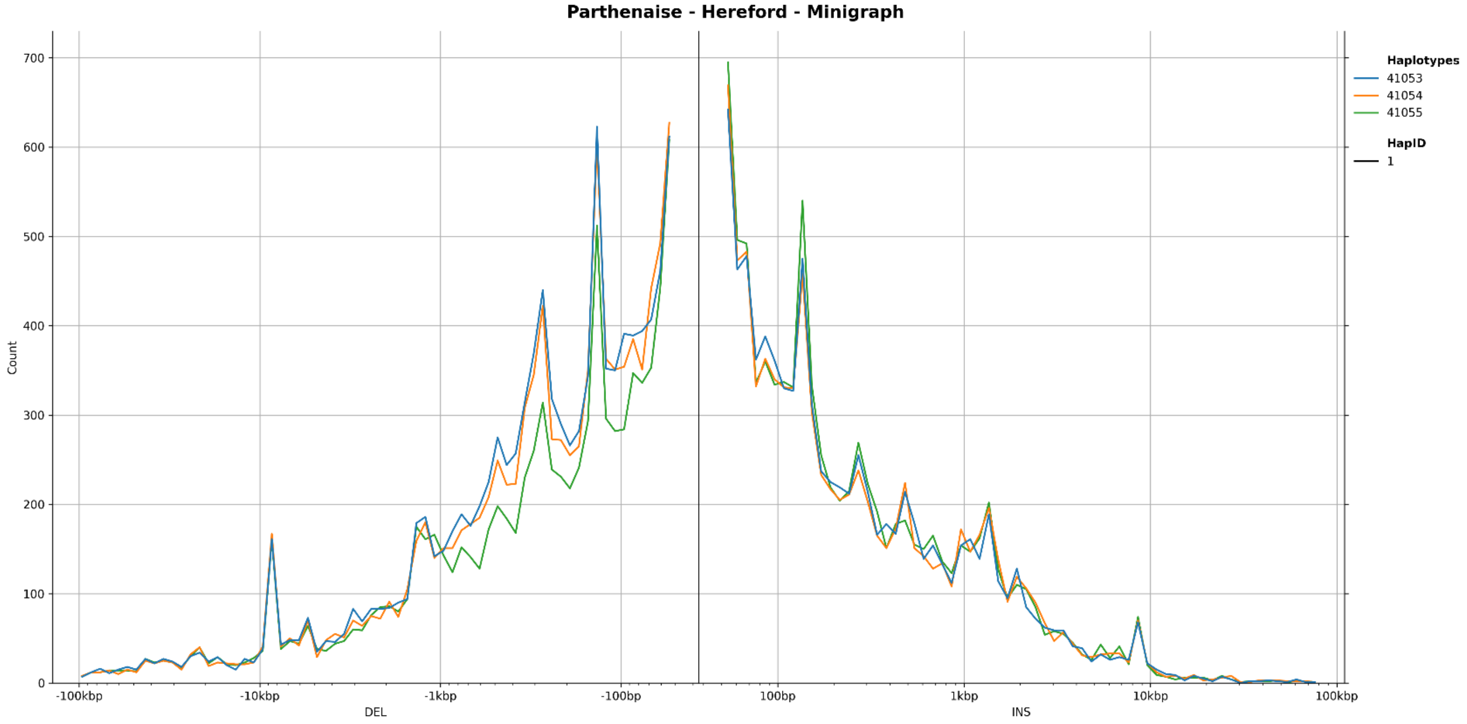

b

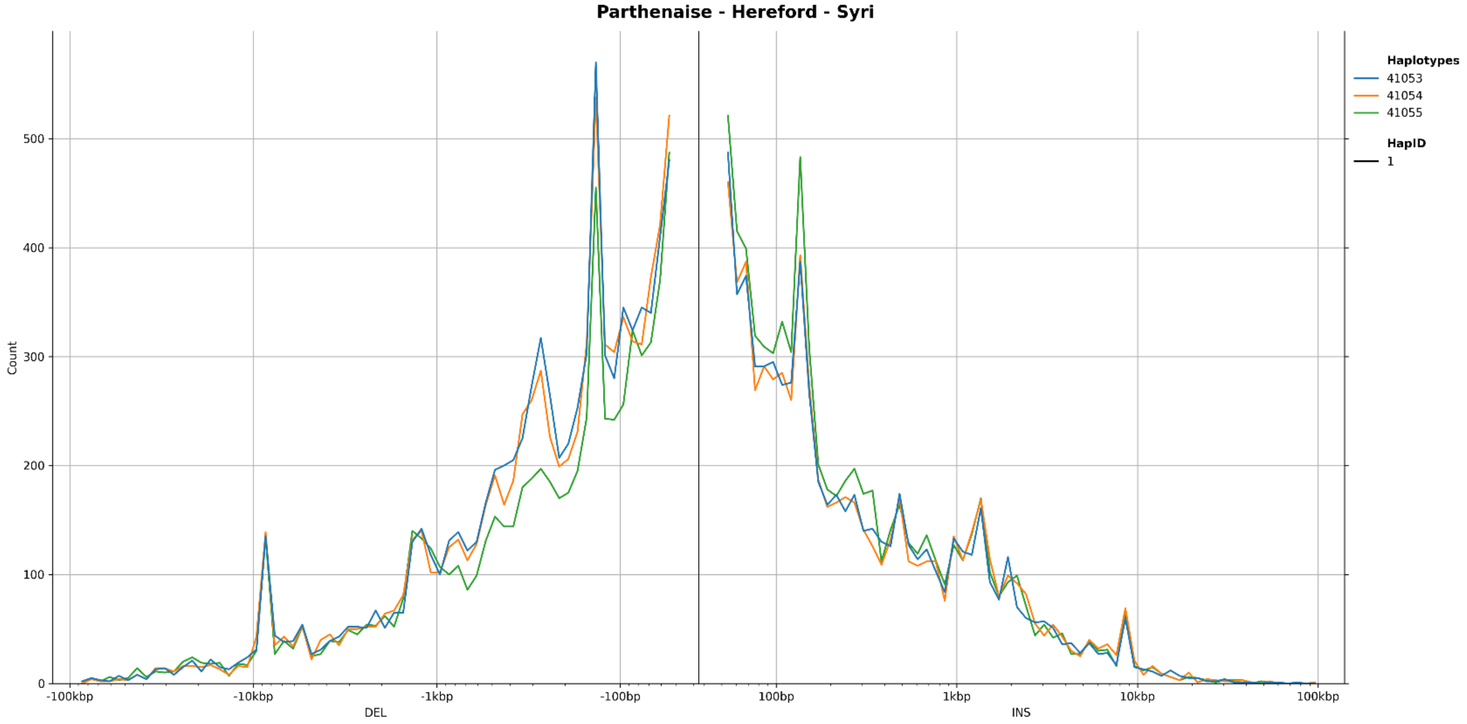

Figure S24 Size distribution of SVs classified as deletions (left) and insertions (right), identified using **a)** Minigraph, and **b)** SyRI for the Parthenaise breed

a

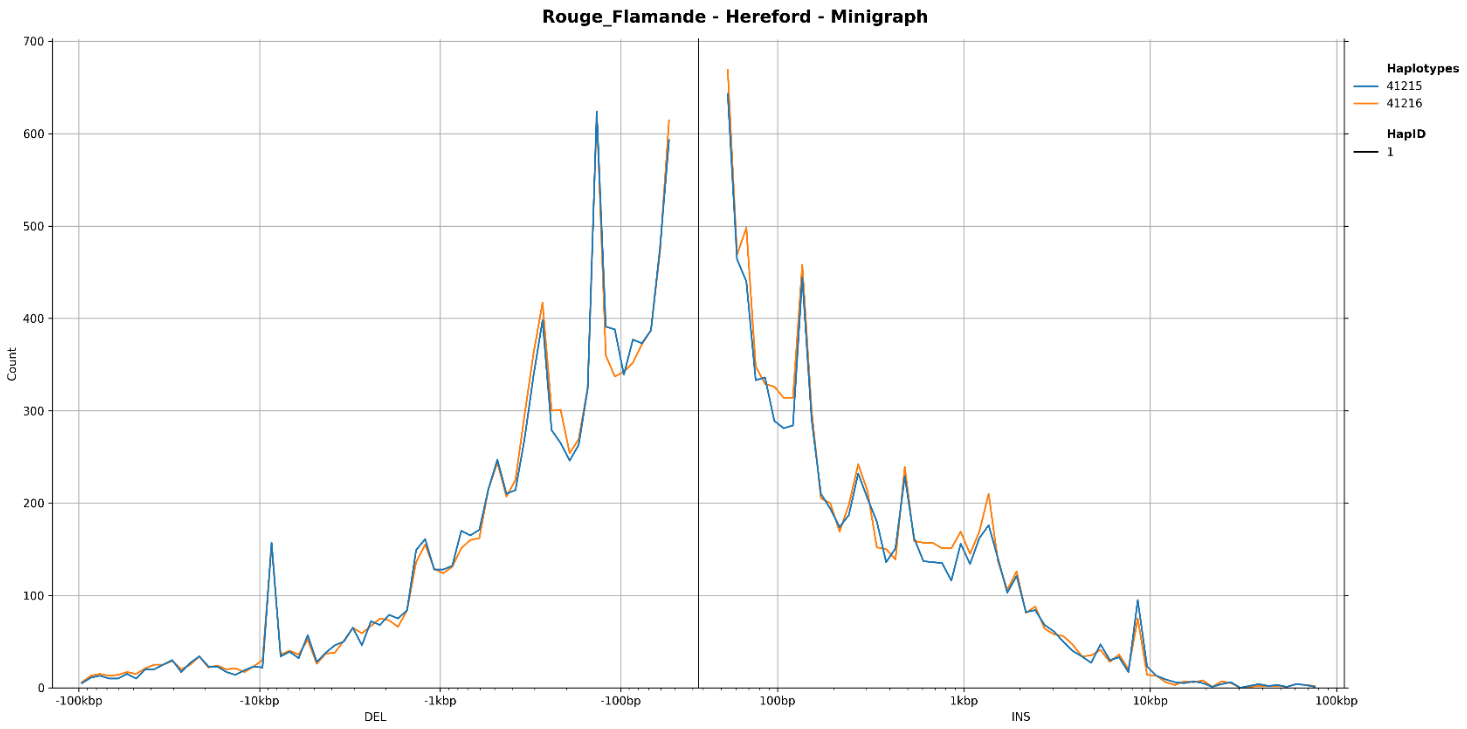

b

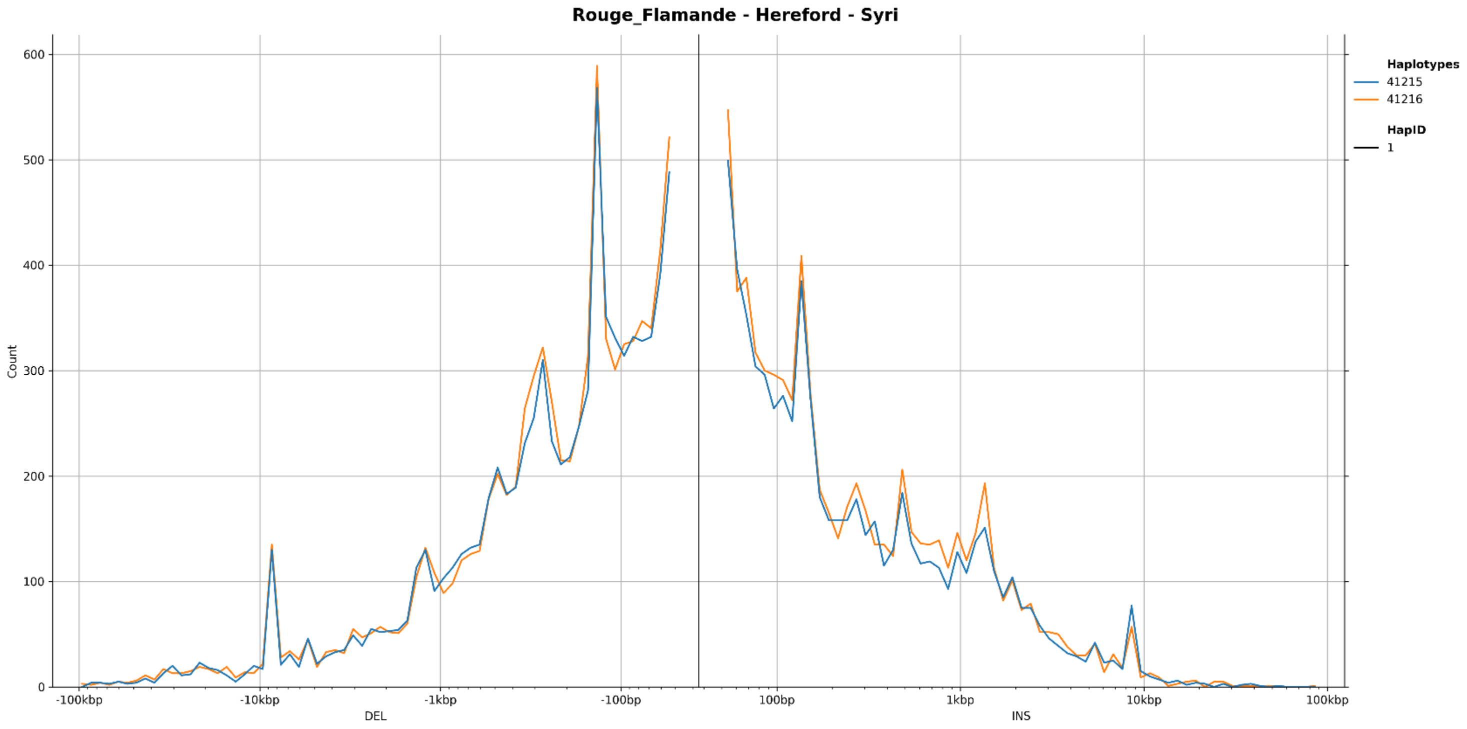

Figure S25 Size distribution of SVs classified as deletions (left) and insertions (right), identified using **a)** Minigraph, and **b)** SyRI for the Rouge Flamande breed

a

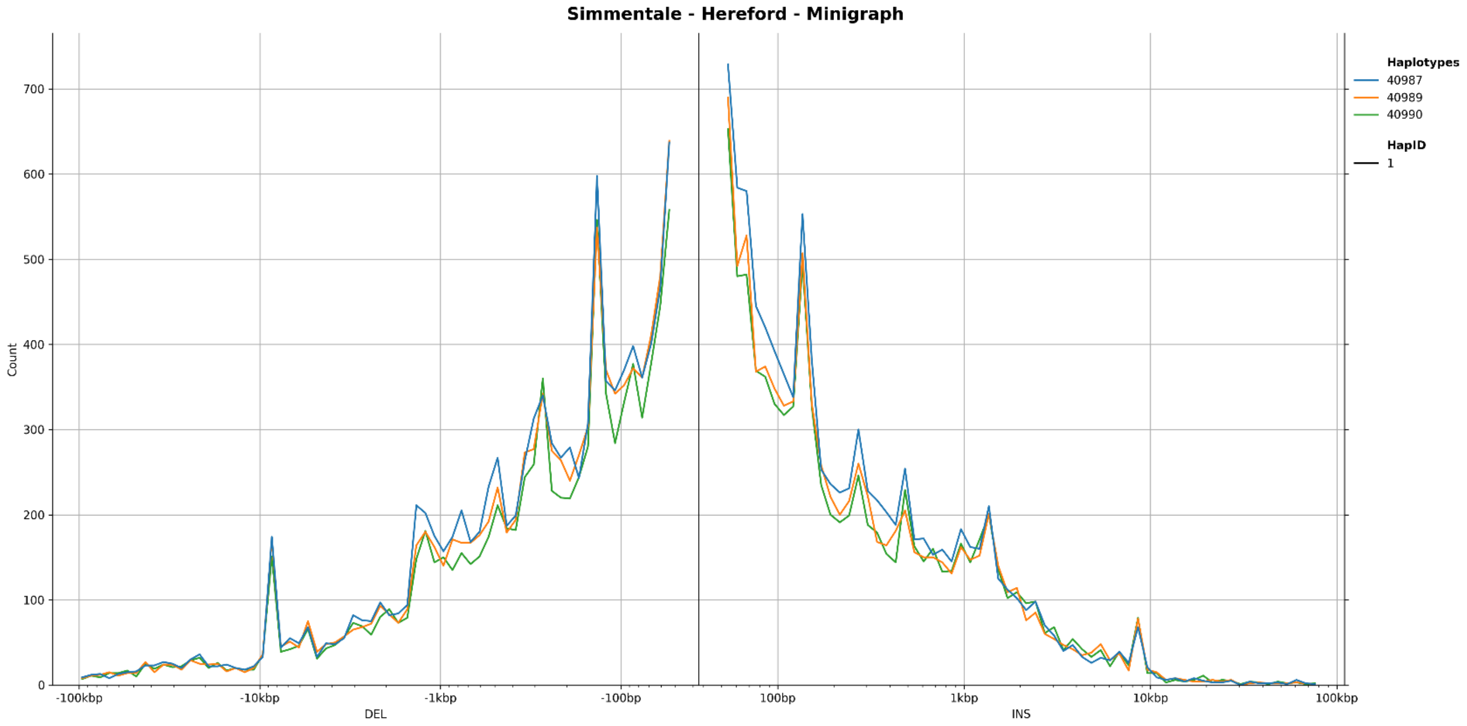

b

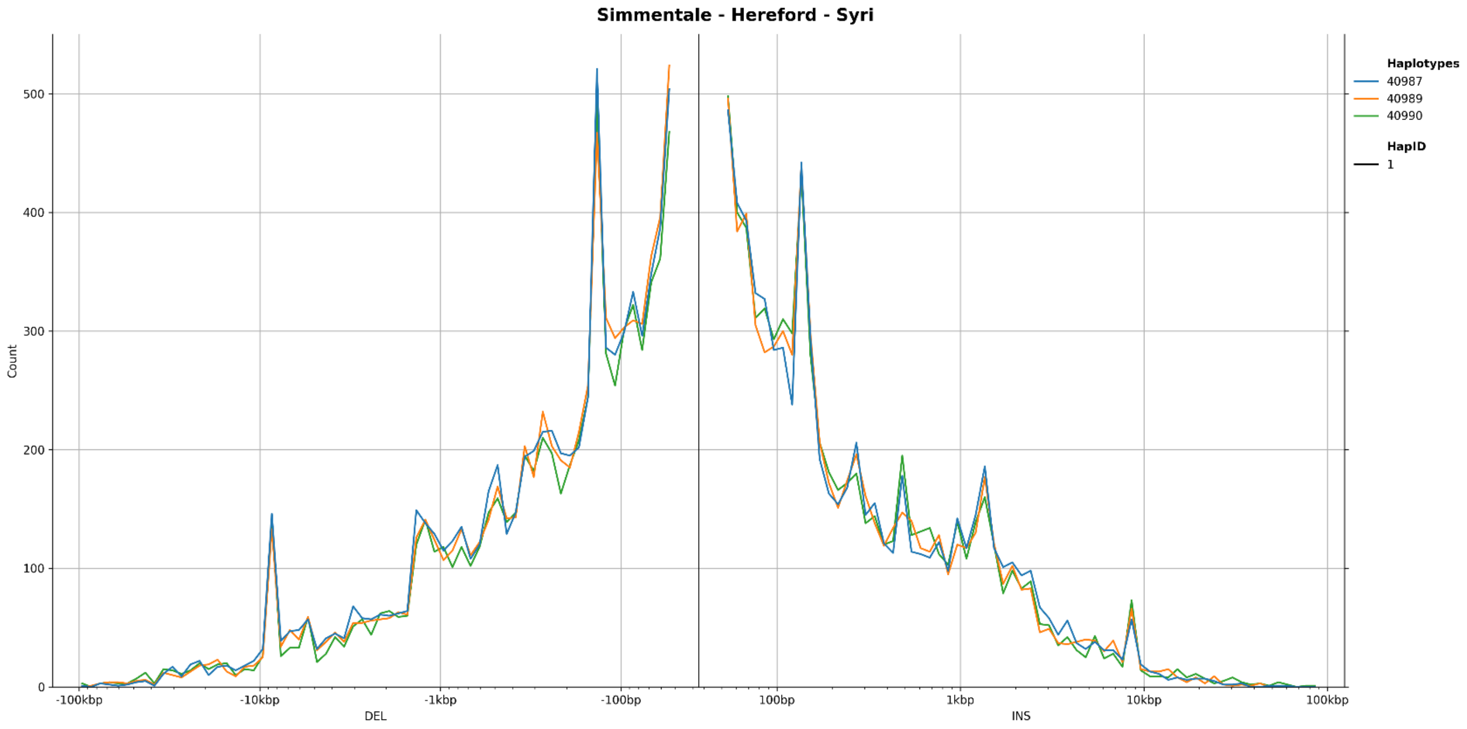

Figure S26 Size distribution of SVs classified as deletions (left) and insertions (right), identified using **a)** Minigraph, and **b)** SyRI for the Simmentale breed

a

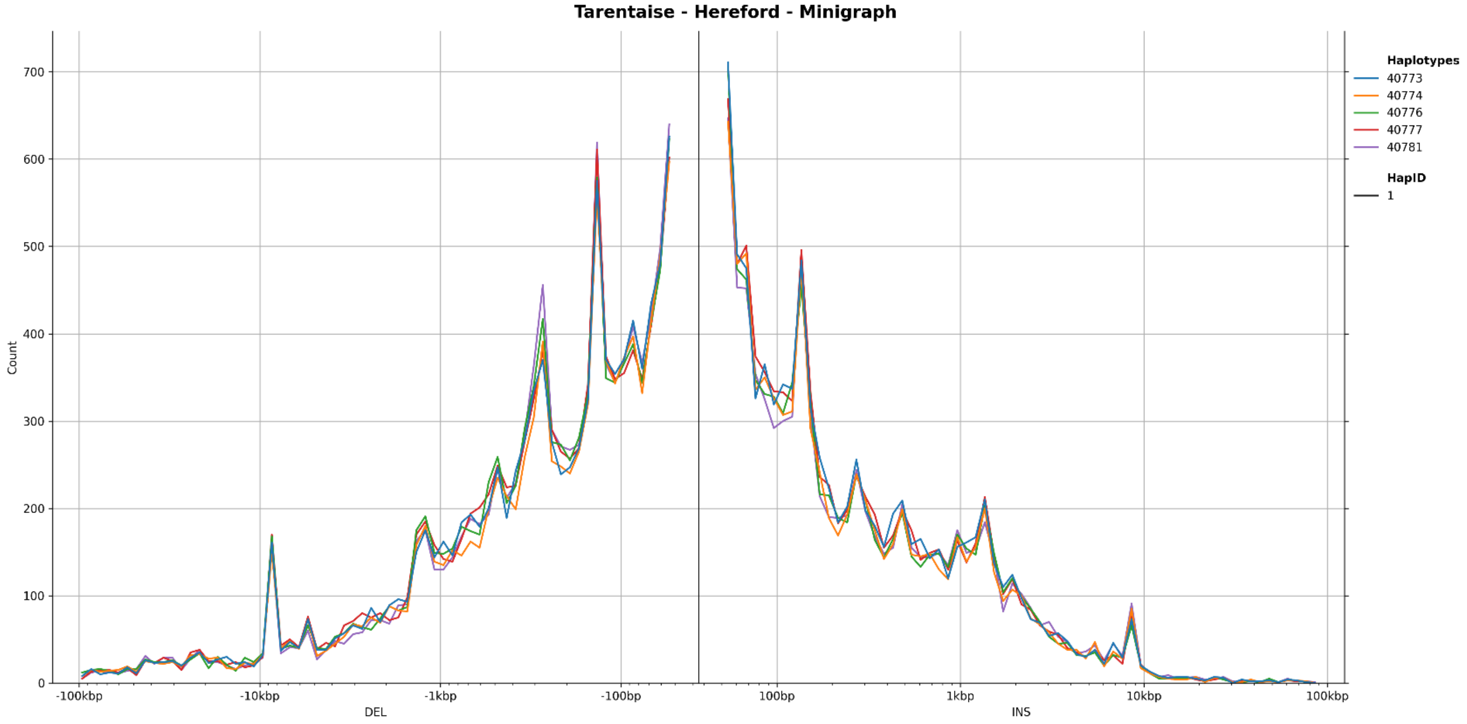

b

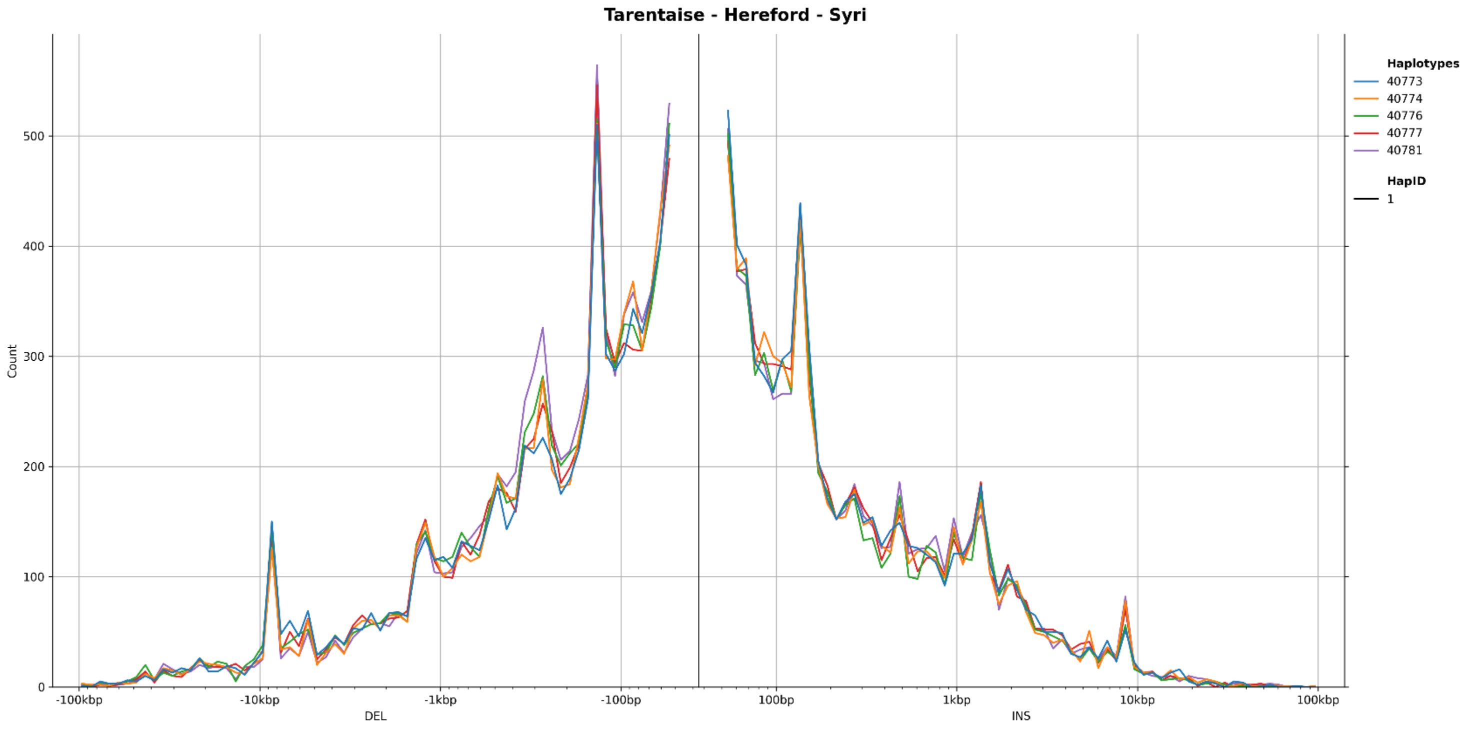

Figure S27 Size distribution of SVs classified as deletions (left) and insertions (right), identified using **a)** Minigraph, and **b)** SyRI for the Tarentaise breed

a

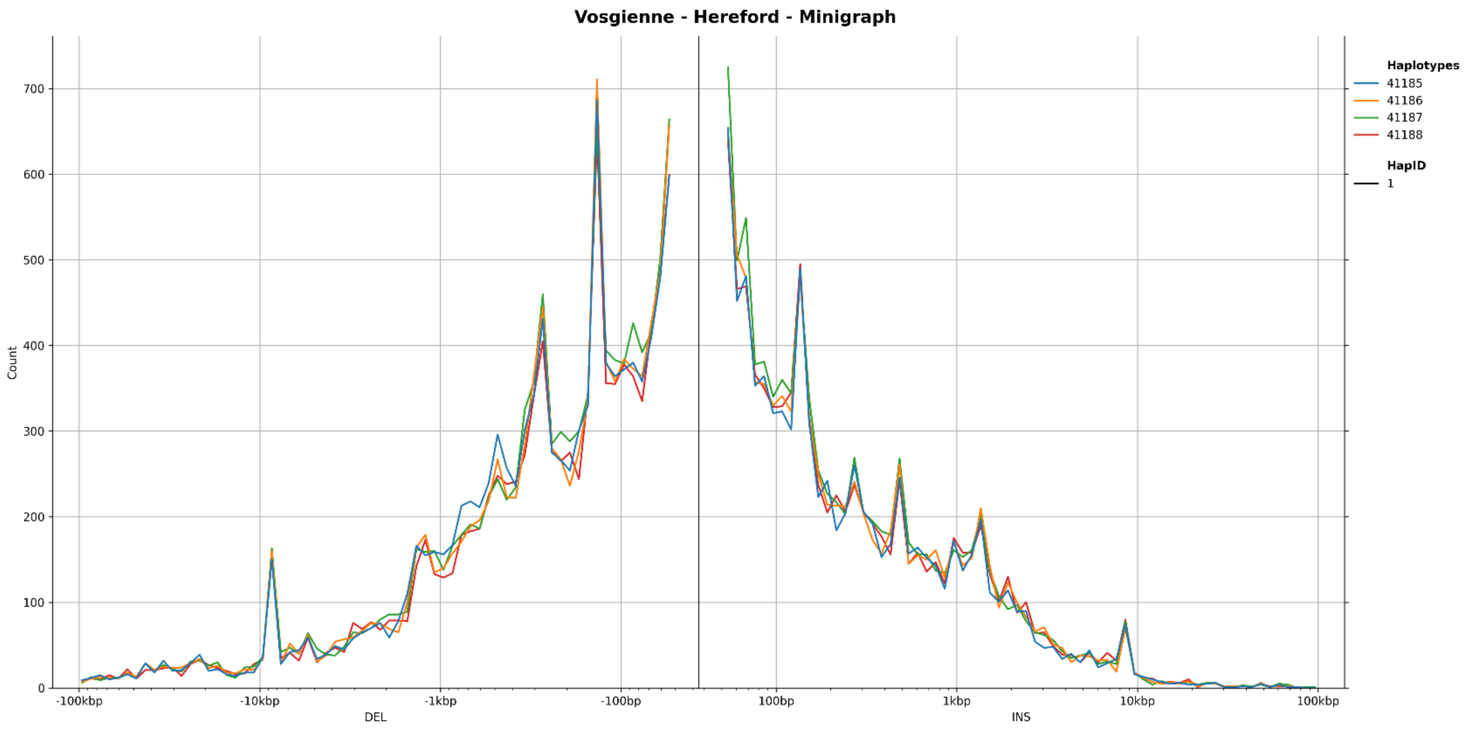

b

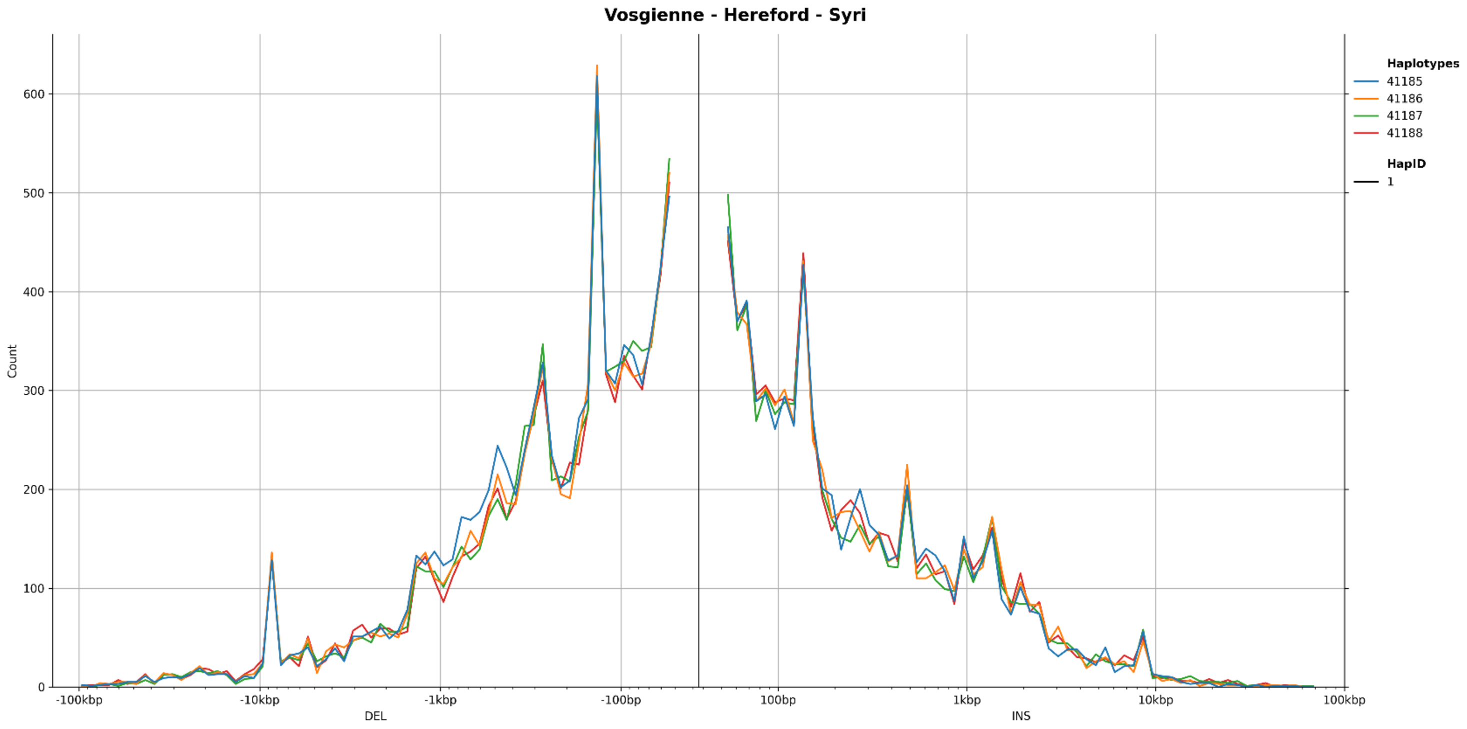

Figure S28 Size distribution of SVs classified as deletions (left) and insertions (right), identified using **a)** Minigraph, and **b)** SyRI for the Vosgienne breed
