# Supplementary material for: Application of a French cattle pangenome, from structural variant discovery to association studies on key phenotypes
Source: Genet Sel Evol. 2025 Oct 23;57:61. doi: 10.1186/s12711-025-01012-x (PMC12551211; doi:10.1186/s12711-025-01012-x)
Supplement: Supplementary file 6 — Supplementary Material 6 Figures S29-S41 Manhattan plots of each GWAS analyses for the three French main dairy breeds. Description: Plots were presented by phenotype analysed in the following order: heifer conception rate, cow conception rate, calving-first artificial insemination interval (AI1), heifer non-return rate, cow non-return rate, milk yield, fat content, protein content, fat yield, protein yield, somatic cell score, clinical mastitis, and height at sacrum [file 12711_2025_1012_MOESM6_ESM.pdf]

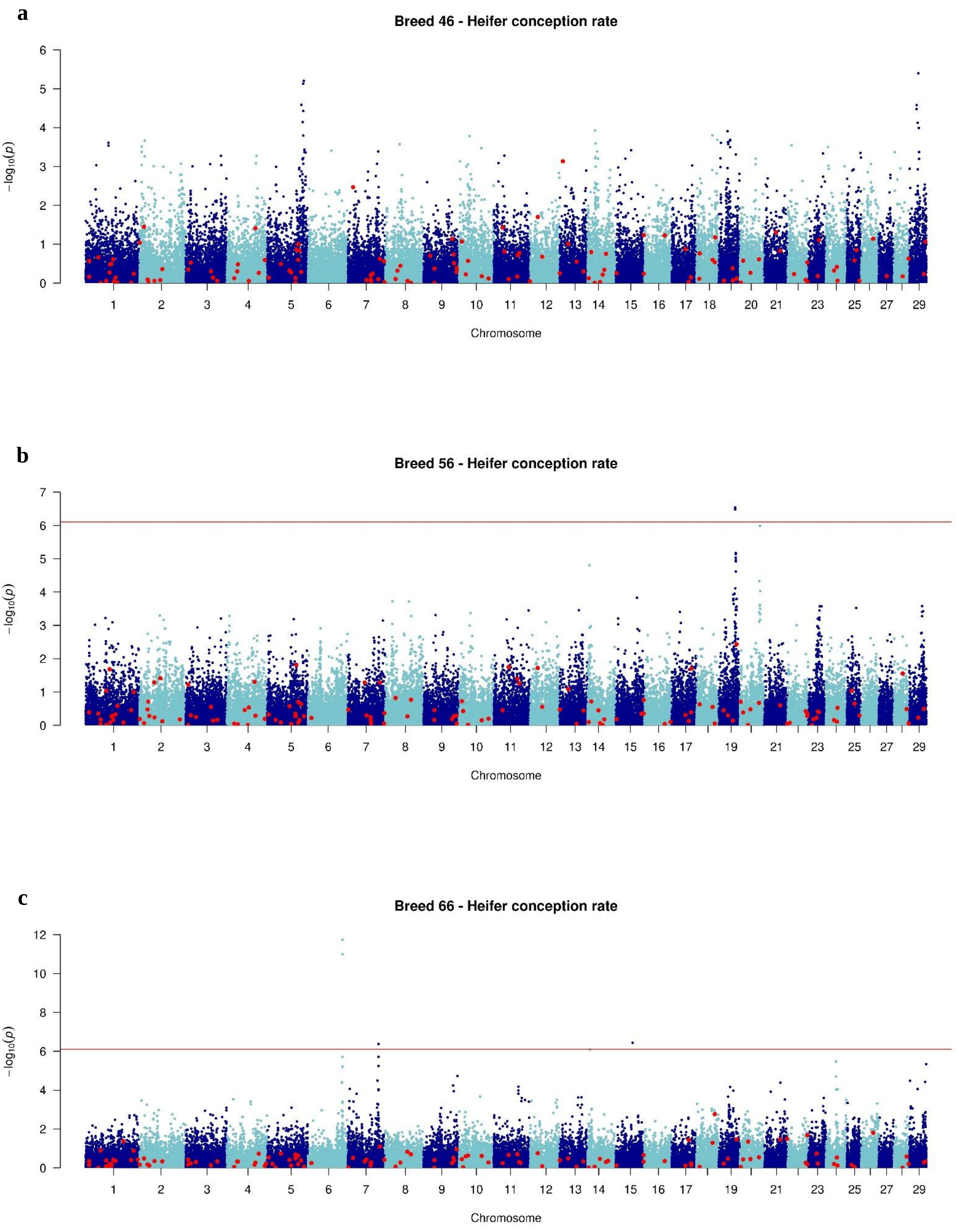

Figure S29 Manhattan plot of GWAS analysis: -log$_{10}$(P) values plotted against the positions of *Bos taurus* autosomes for variants associated with heifer conception rate in **a)** Montbéliarde, **b)** Normande, and **c)** Holstein bulls

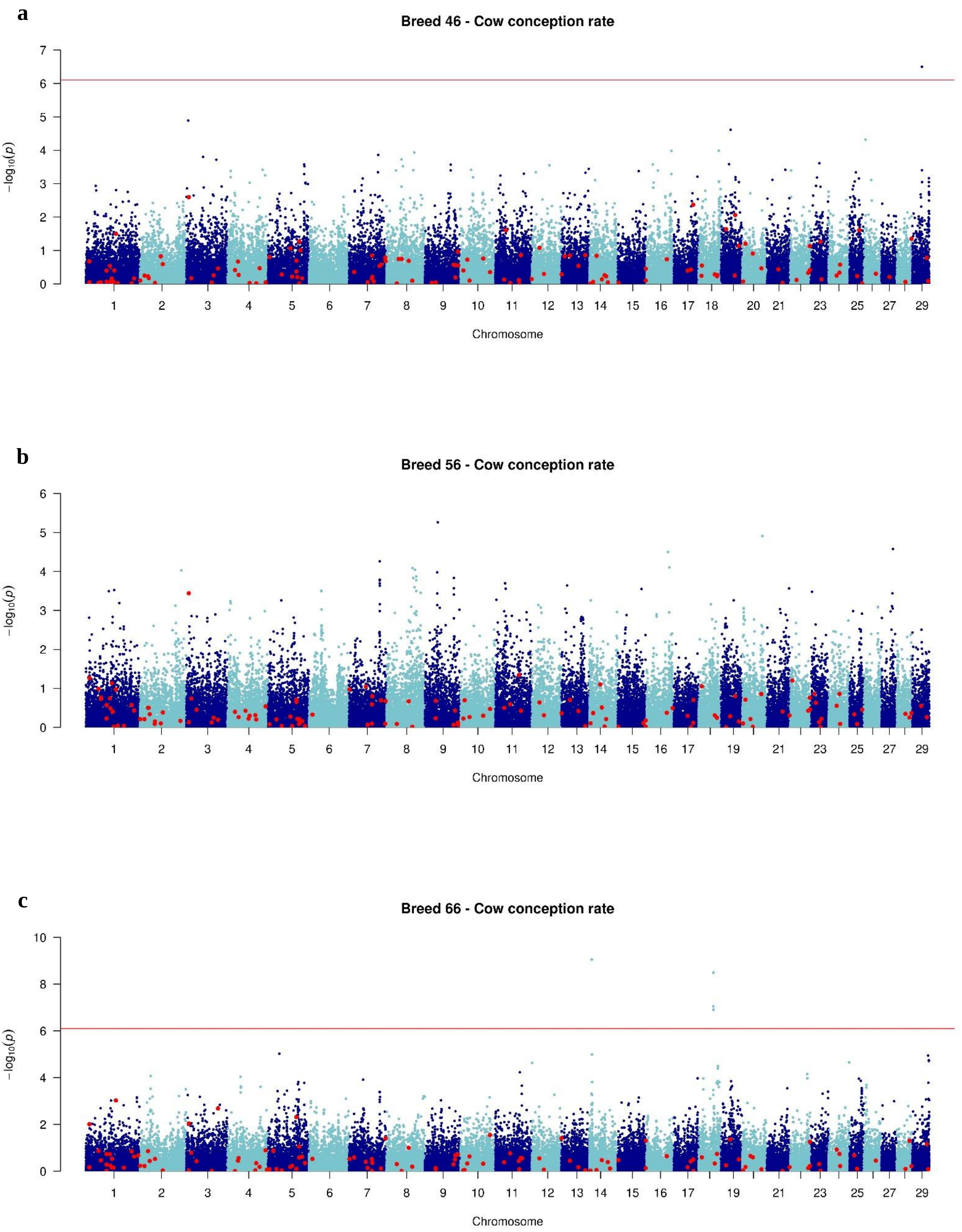

Figure S30 Manhattan plot of GWAS analysis: -$\log_{10}$(P) values plotted against the positions of *Bos taurus* autosomes for variants associated with cow conception rate in **a)** Montbéliarde, **b)** Normande, and **c)** Holstein bulls

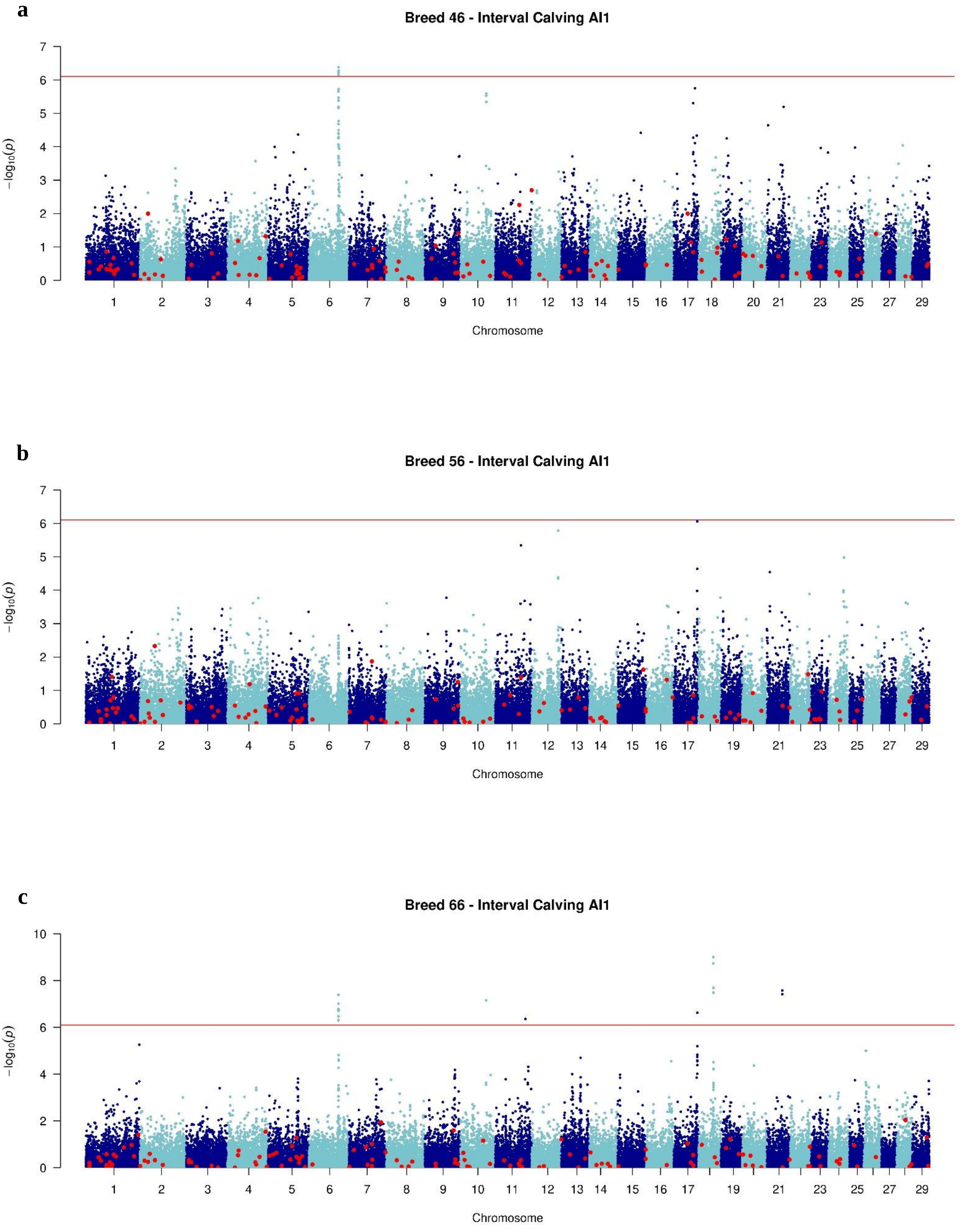

Figure S31 Manhattan plot of GWAS analysis: -$\log_{10}$(P) values plotted against the positions of *Bos taurus* autosomes for variants associated with interval calving-AI1 in **a)** Montbéliarde, **b)** Normande, and **c)** Holstein bulls

a

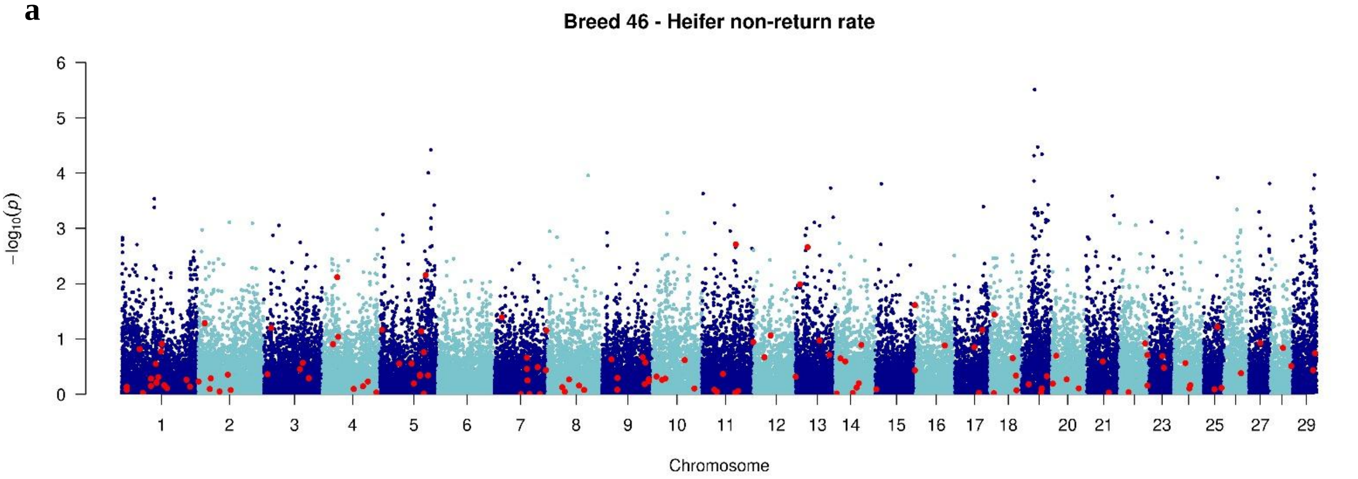

b

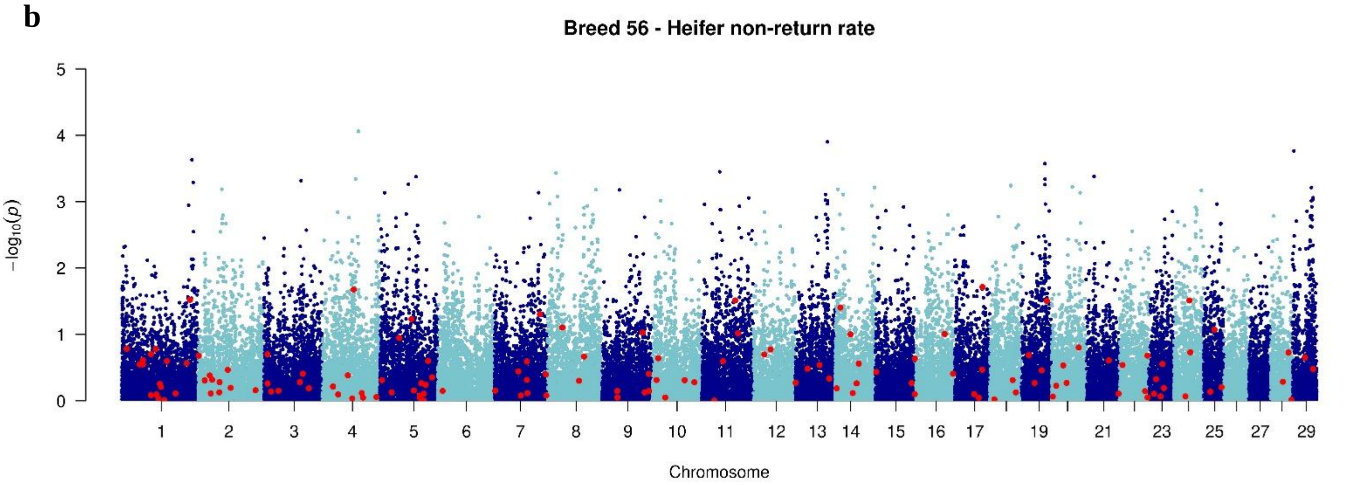

c

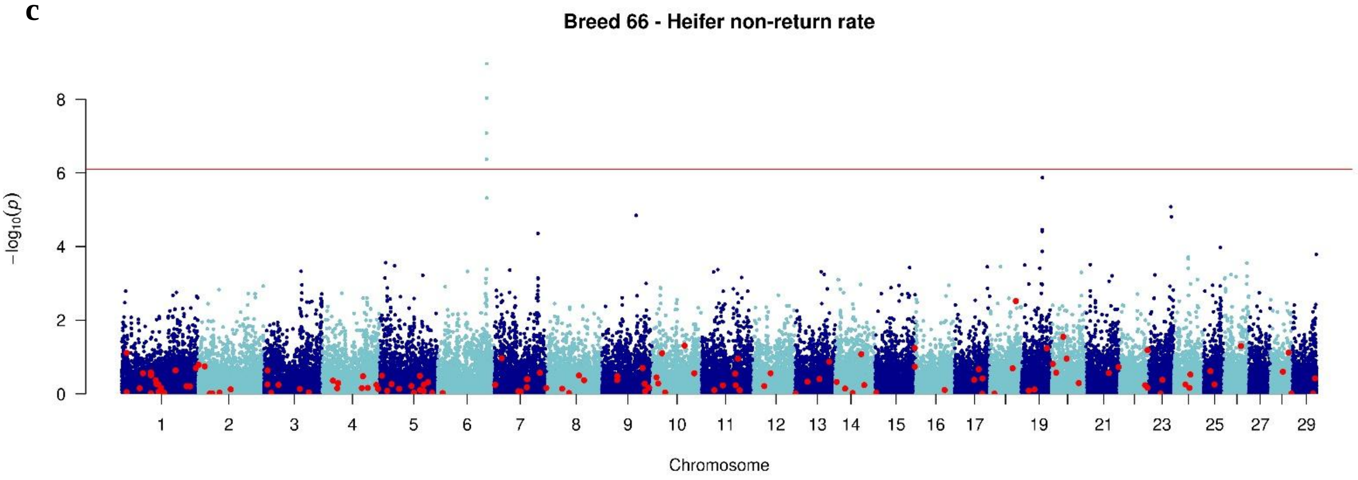

Figure S32 Manhattan plot of GWAS analysis: -$\log_{10}$(P) values plotted against the positions of *Bos taurus* autosomes for variants associated with heifer non-return rate in **a)** Montbéliarde, **b)** Normande, and **c)** Holstein bulls

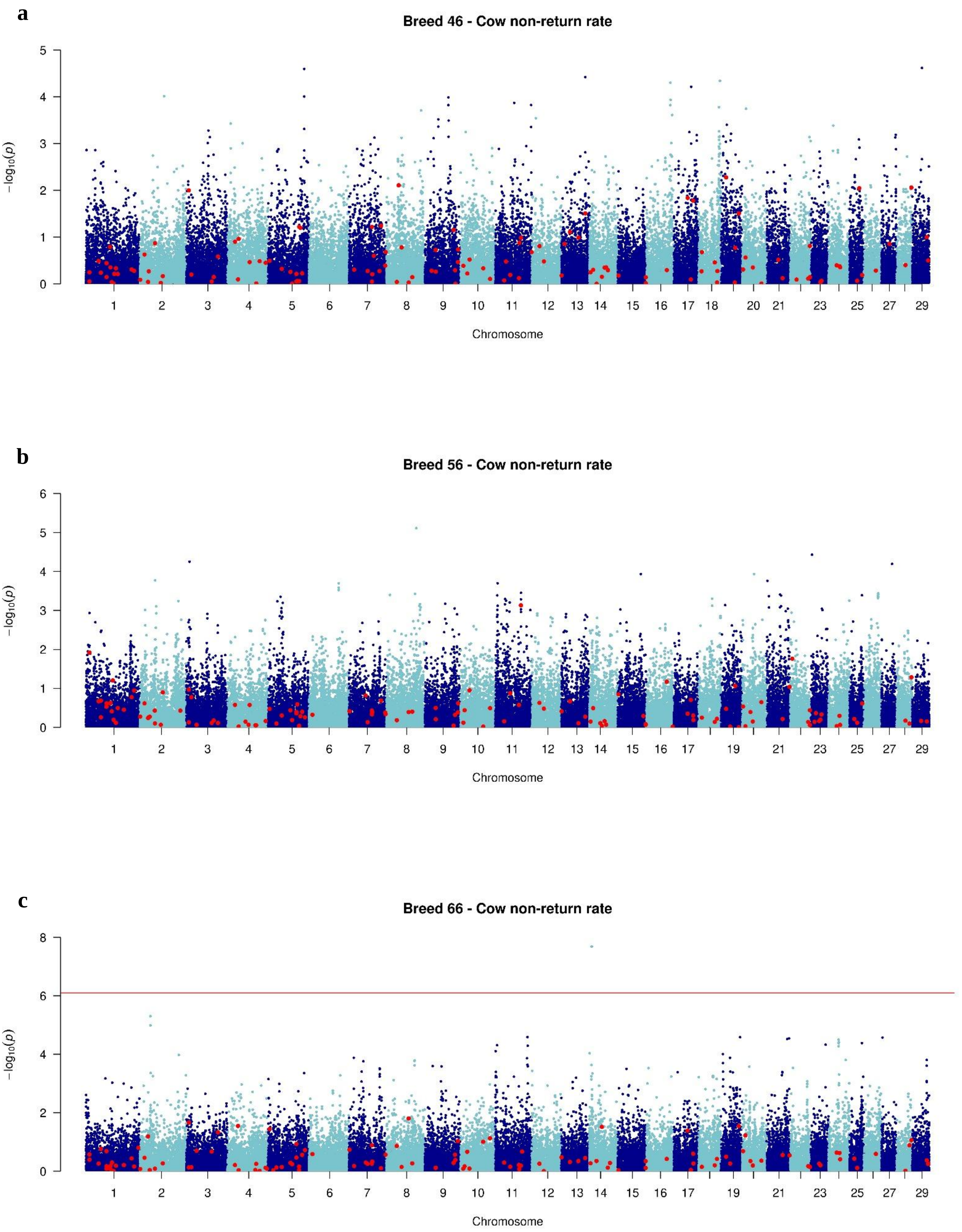

Figure S33 Manhattan plot of GWAS analysis: -$\log_{10}$(P) values plotted against the positions of *Bos taurus* autosomes for variants associated with cow non-return rate in **a)** Montbéliarde, **b)** Normande, and **c)** Holstein bulls

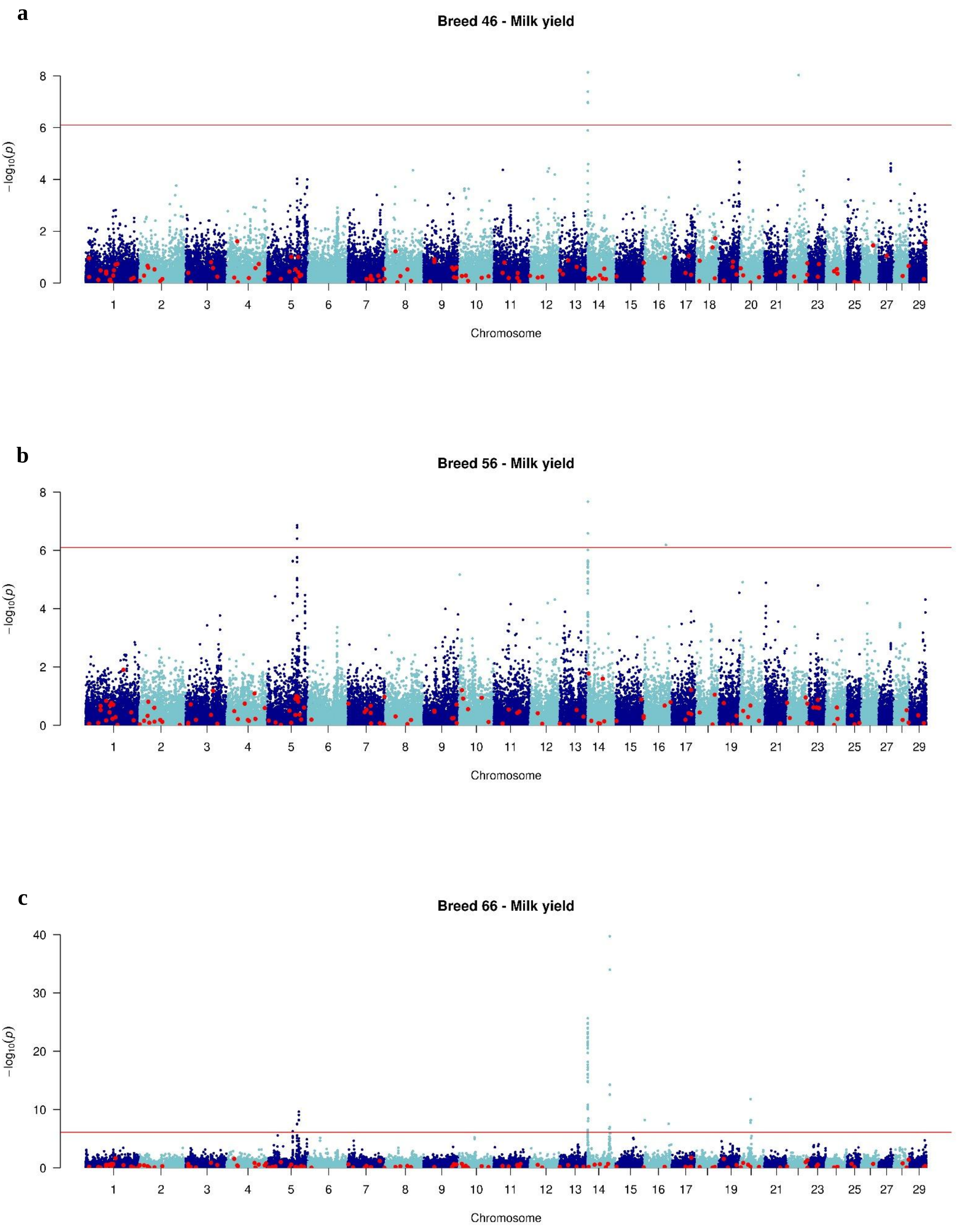

Figure S34 Manhattan plot of GWAS analysis: -$\log_{10}$(P) values plotted against the positions of *Bos taurus* autosomes for variants associated with milk yield in **a)** Montbéliarde, **b)** Normande, and **c)** Holstein bulls

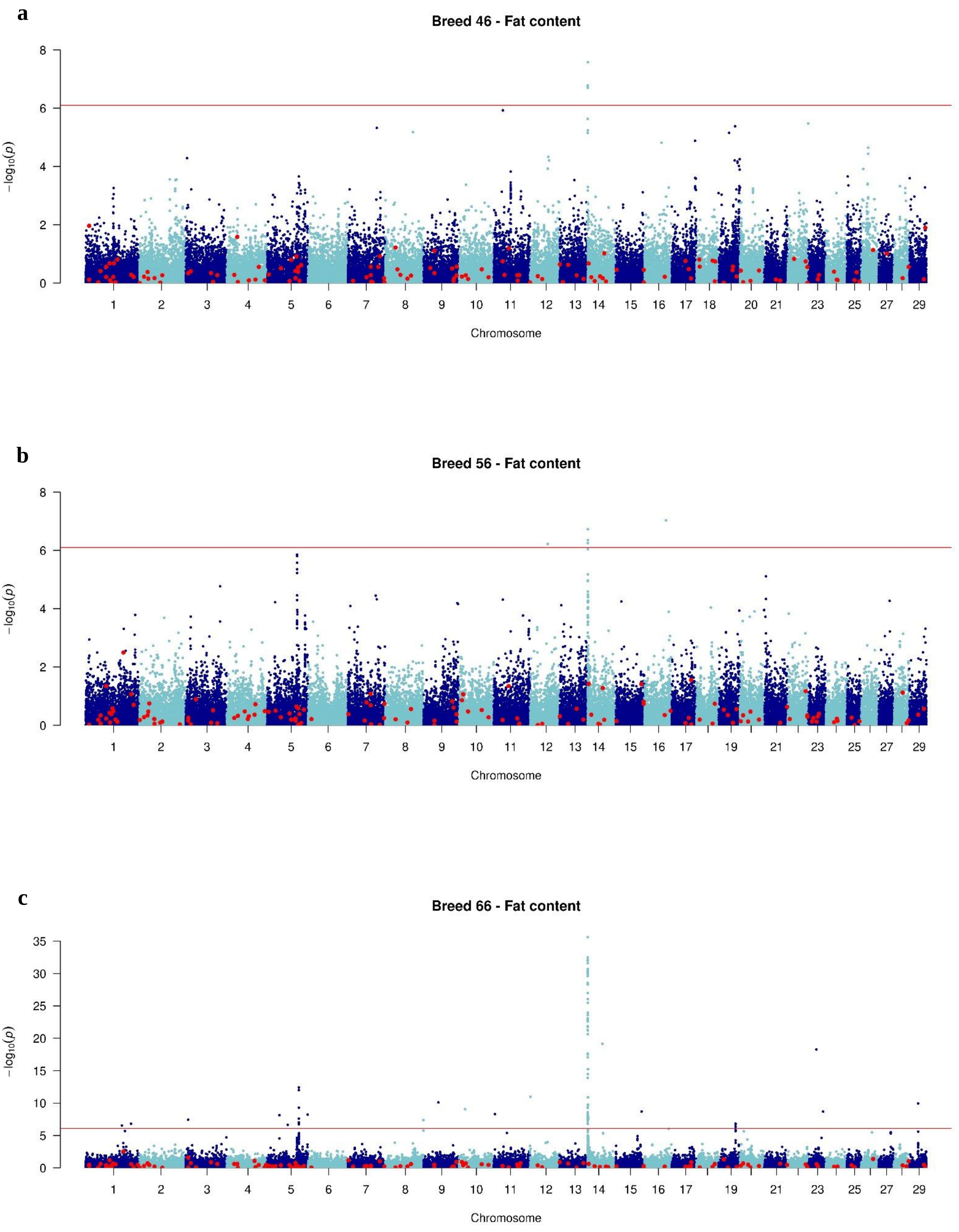

Figure S35 Manhattan plot of GWAS analysis: -$\log_{10}$(P) values plotted against the positions of *Bos taurus* autosomes for variants associated with fat content in **a)** Montbéliarde, **b)** Normande, and **c)** Holstein bulls

a

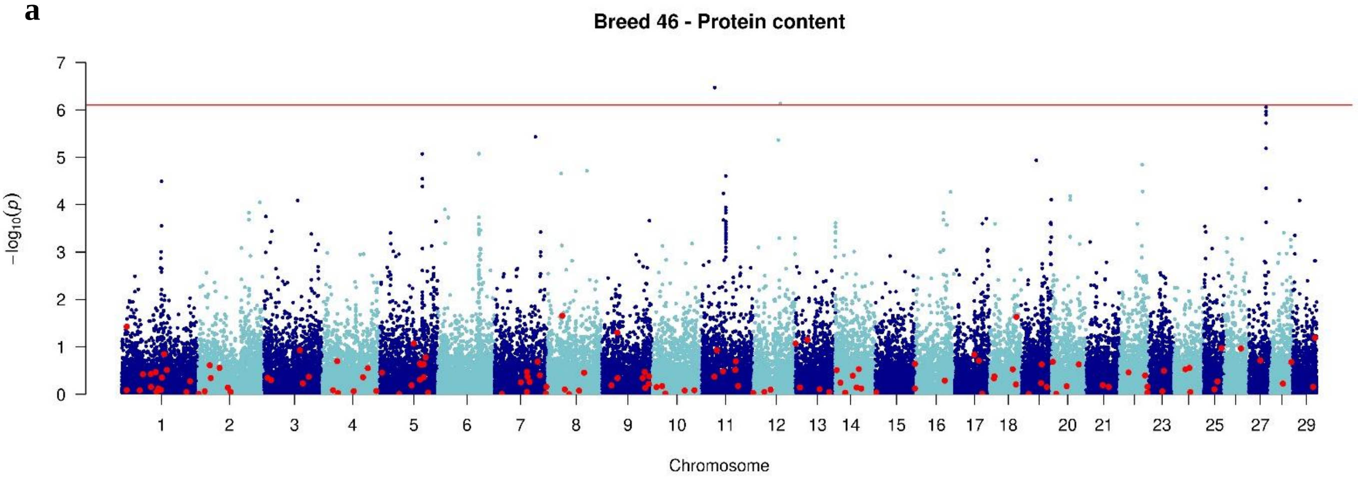

b

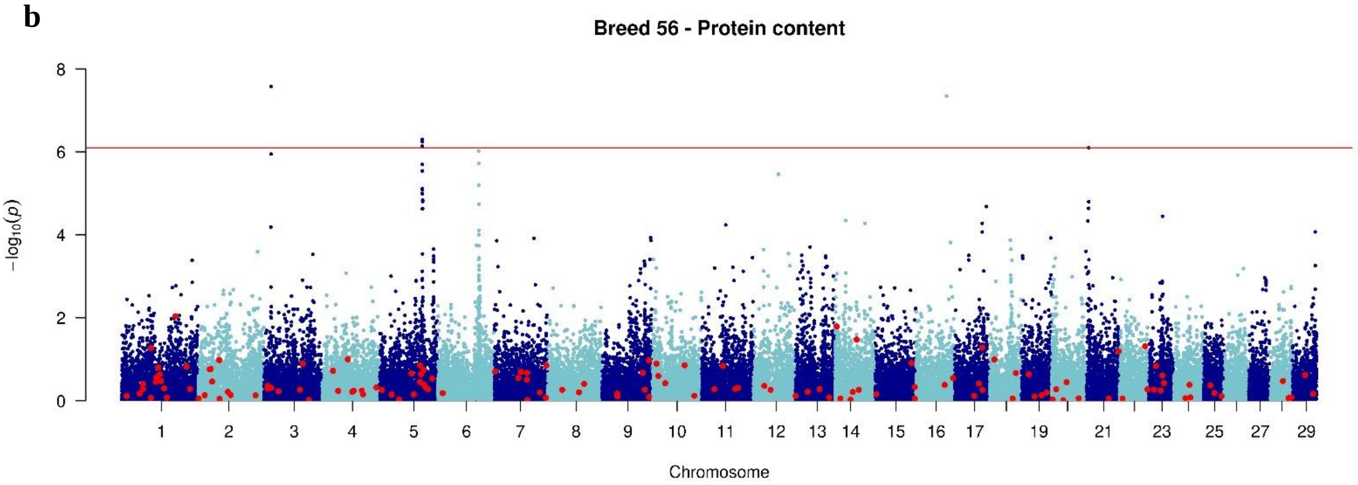

c

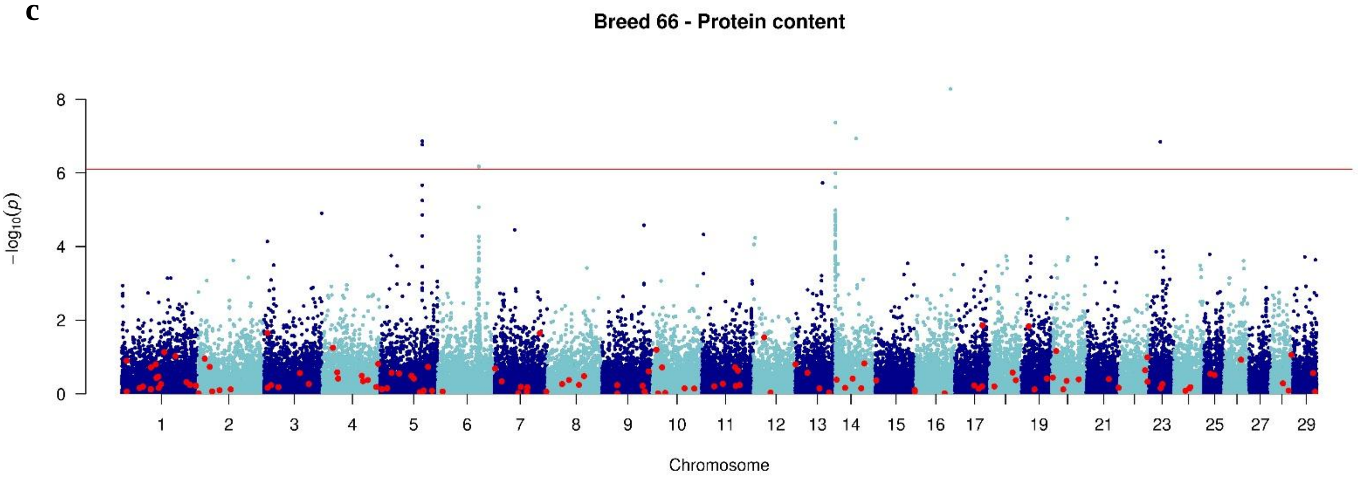

Figure S36 Manhattan plot of GWAS analysis: -$\log_{10}$(P) values plotted against the positions of *Bos taurus* autosomes for variants associated with protein content in **a)** Montbéliarde, **b)** Normande, and **c)** Holstein bulls

a

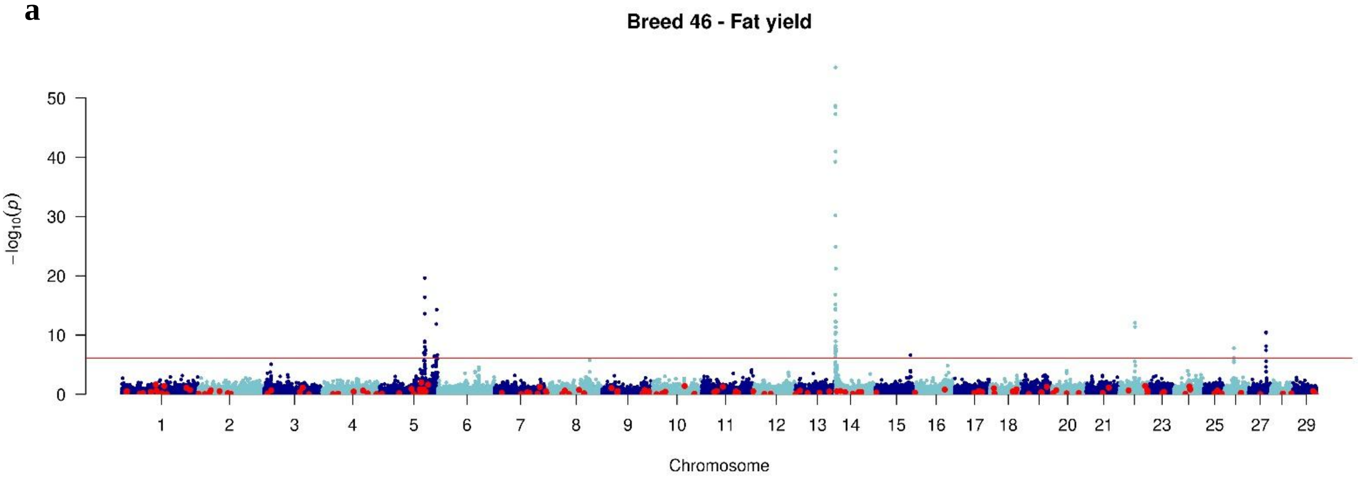

b

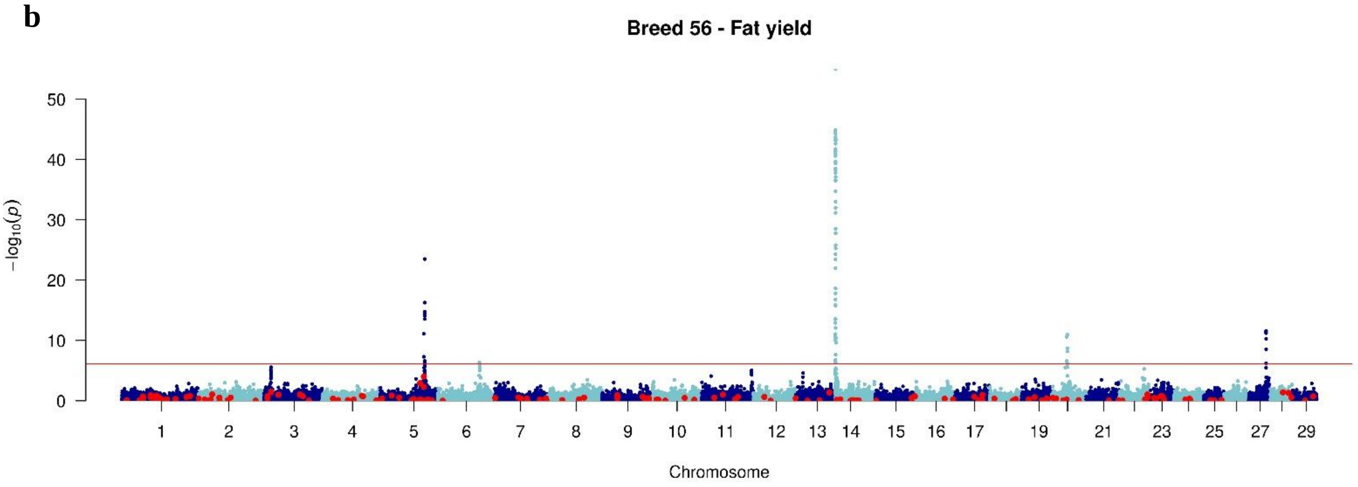

c

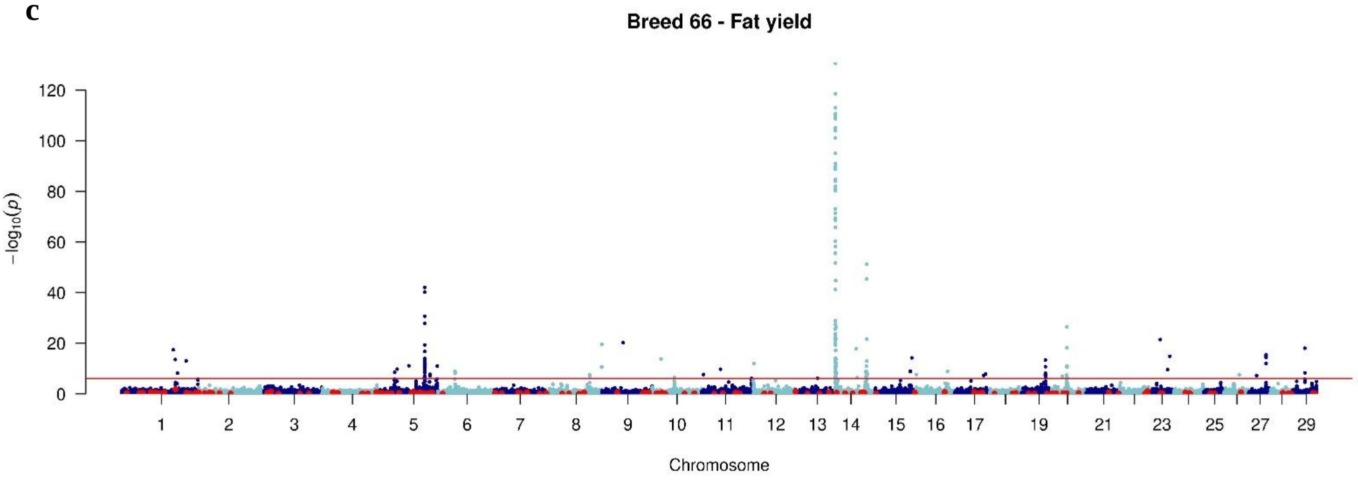

Figure S37 Manhattan plot of GWAS analysis: -$\log_{10}$(P) values plotted against the positions of *Bos taurus* autosomes for variants associated with fat yield in **a)** Montbéliarde, **b)** Normande, and **c)** Holstein bulls

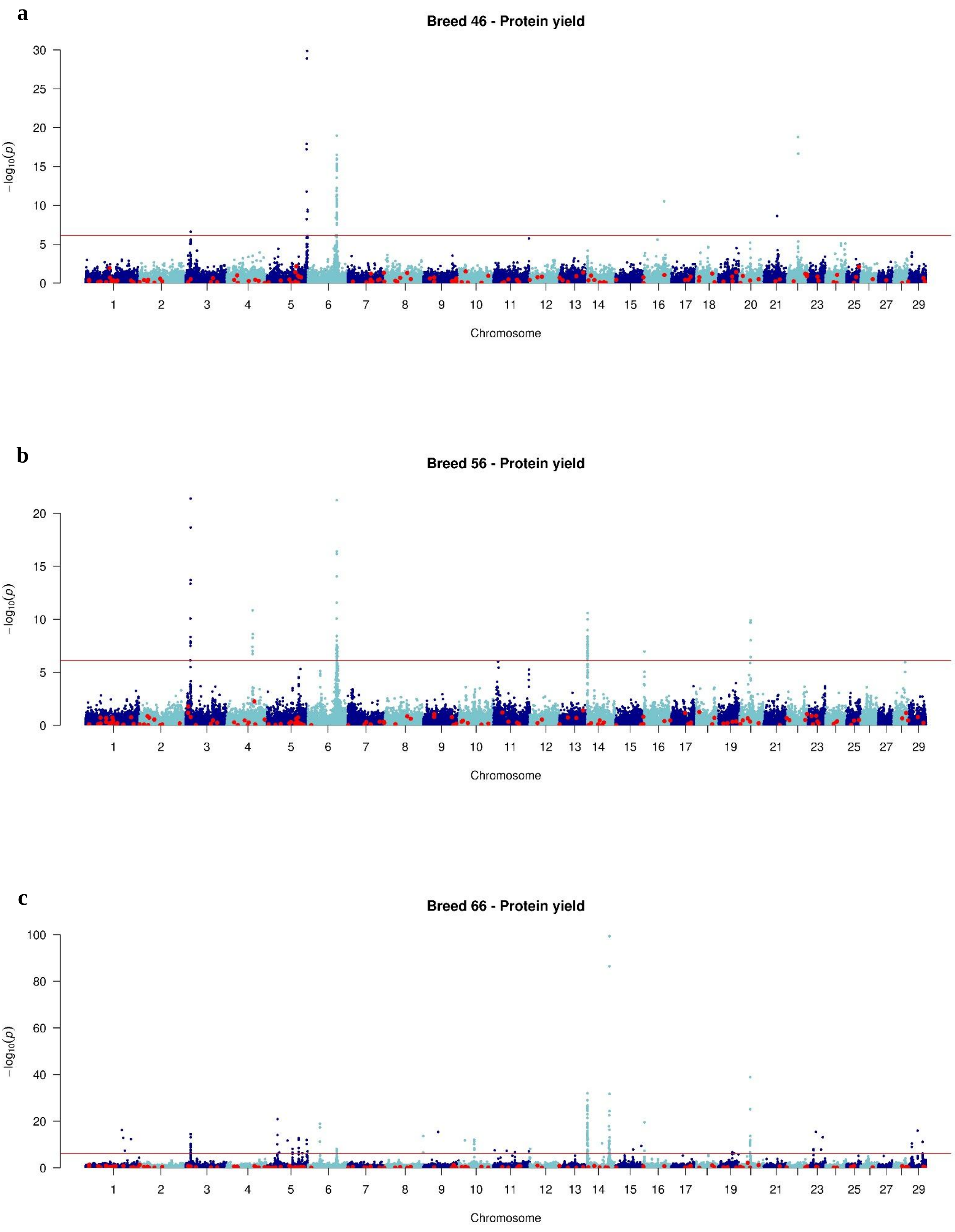

Figure S38 Manhattan plot of GWAS analysis: -log$_{10}$(P) values plotted against the positions of *Bos taurus* autosomes for variants associated with protein yield in **a)** Montbéliarde, **b)** Normande, and **c)** Holstein bulls

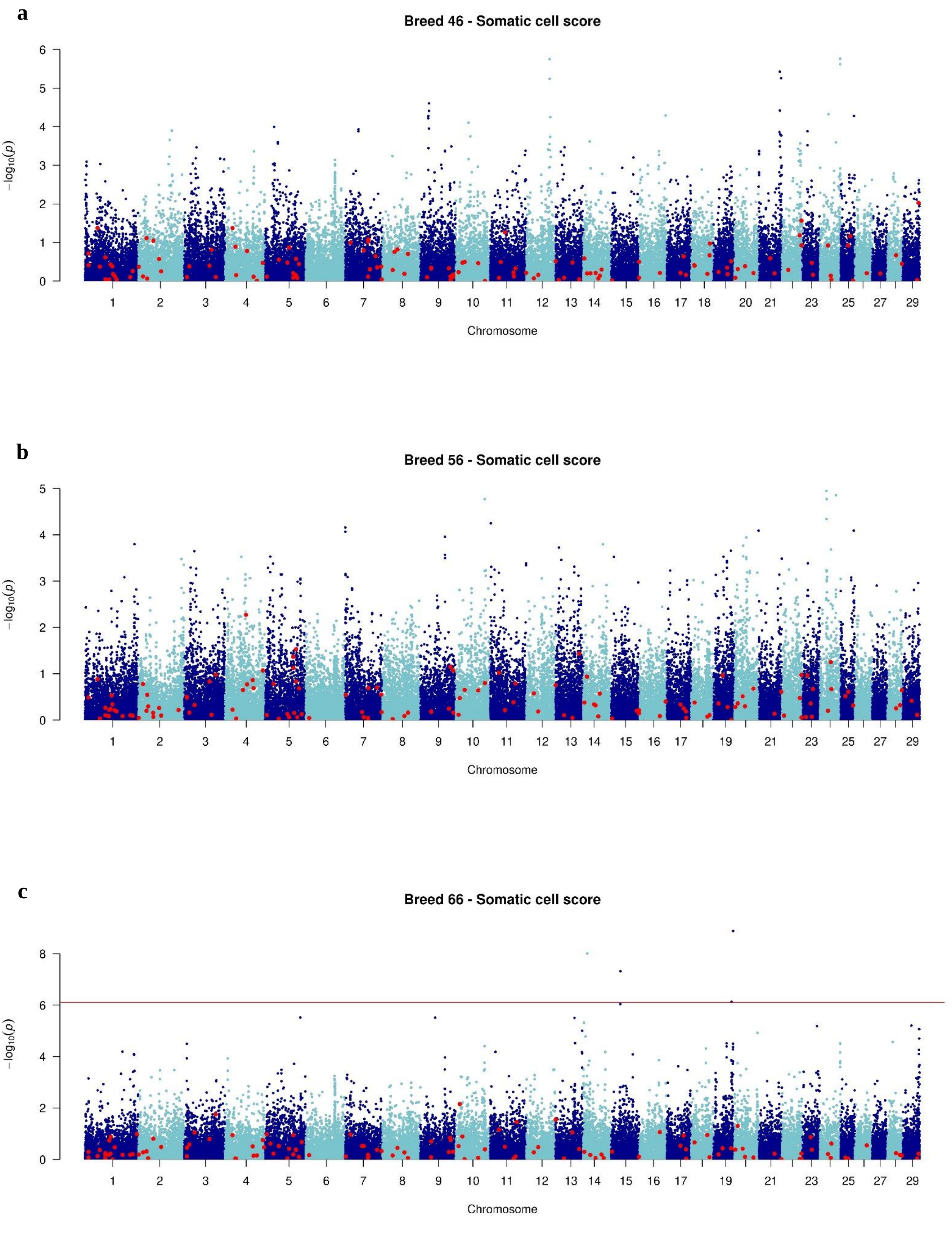

Figure S39 Manhattan plot of GWAS analysis: -log$_{10}$(P) values plotted against the positions of *Bos taurus* autosomes for variants associated with somatic cell score in **a)** Montbéliarde, **b)** Normande, and **c)** Holstein bulls

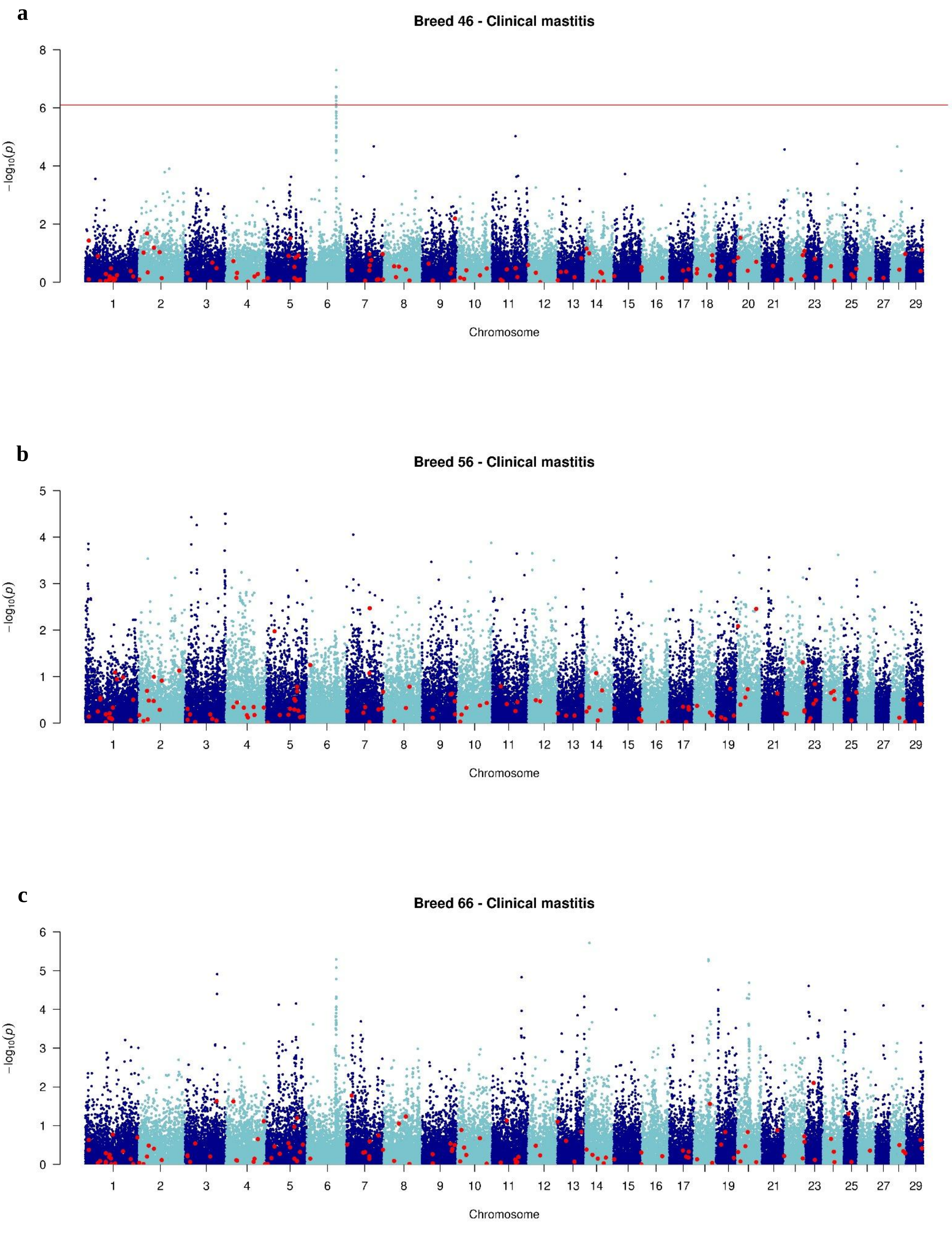

Figure S40 Manhattan plot of GWAS analysis: -$\log_{10}$(P) values plotted against the positions of *Bos taurus* autosomes for variants associated with clinical mastitis in **a)** Montbéliarde, **b)** Normande, and **c)** Holstein bulls

a

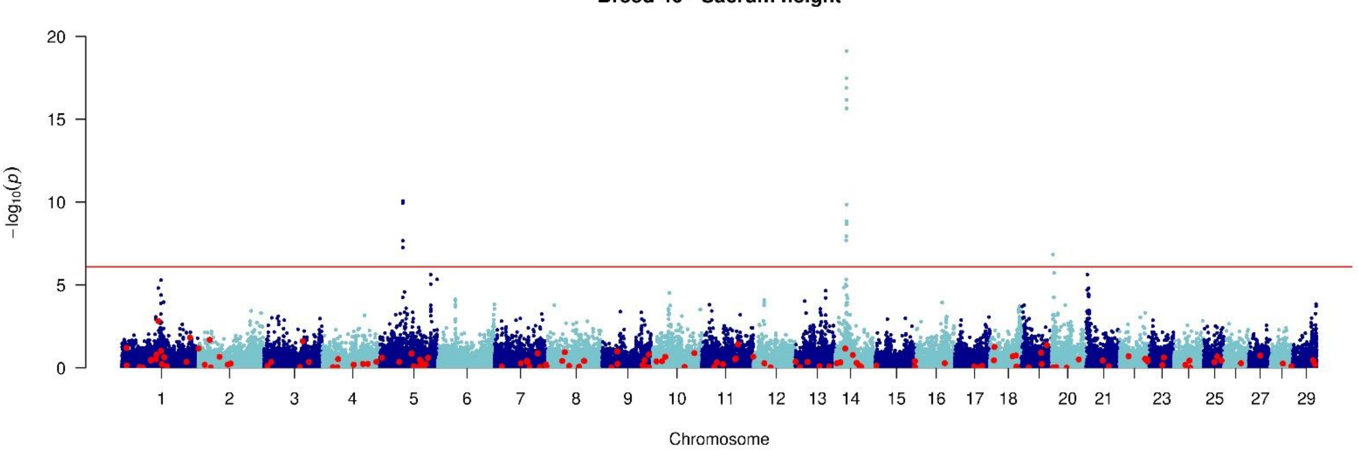

b

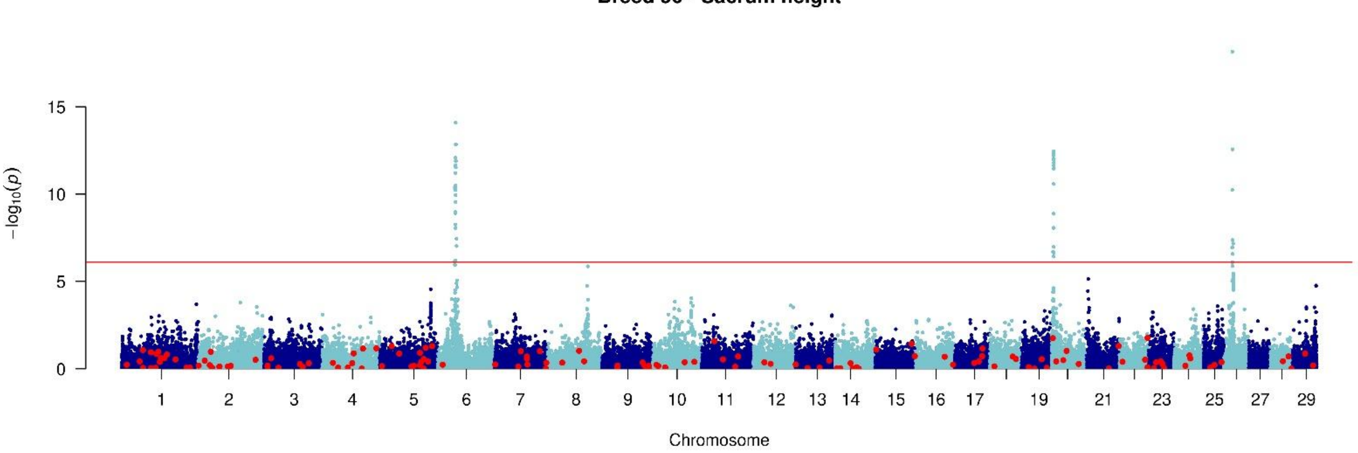

c

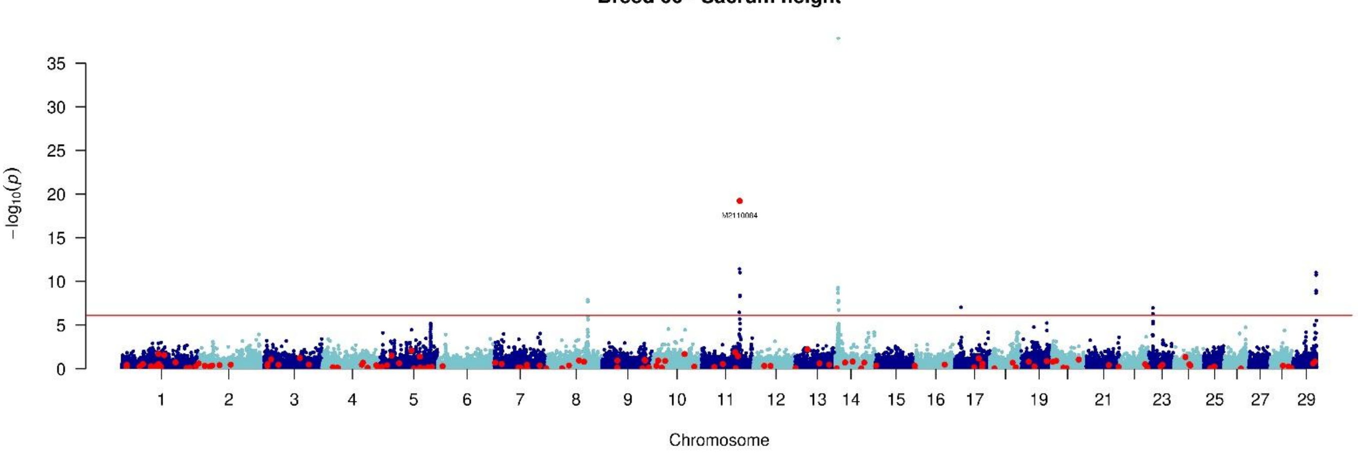

Figure S41 Manhattan plot of GWAS analysis: -$\log_{10}$(P) values plotted against the positions of *Bos taurus* autosomes for variants associated with sacrum height in **a)** Montbéliarde, **b)** Normande, and **c)** Holstein bulls
